# Human Follicular Mites: Ectoparasites Becoming Symbionts

**DOI:** 10.1093/molbev/msac125

**Published:** 2022-06-21

**Authors:** Gilbert Smith, Alejandro Manzano-Marín, Mariana Reyes-Prieto, Cátia Sofia Ribeiro Antunes, Victoria Ashworth, Obed Nanjul Goselle, Abdulhalem Abdulsamad A Jan, Andrés Moya, Amparo Latorre, M Alejandra Perotti, Henk R Braig

**Affiliations:** School of Natural Sciences, Bangor University, Bangor, Wales, United Kingdom; Centre for Microbiology and Environmental Systems Science (CMESS), University of Vienna, Vienna, Austria; Institute of Integrative Systems Biology (I2Sysbio), Universitat de València and Spanish Research Council (CSIC), València, Spain; Foundation for the Promotion of Health and Biomedical Research of the Valencian Community (FISABIO), València, Spain; School of Natural Sciences, Bangor University, Bangor, Wales, United Kingdom; School of Natural Sciences, Bangor University, Bangor, Wales, United Kingdom; School of Natural Sciences, Bangor University, Bangor, Wales, United Kingdom; School of Natural Sciences, Bangor University, Bangor, Wales, United Kingdom; Institute of Integrative Systems Biology (I2Sysbio), Universitat de València and Spanish Research Council (CSIC), València, Spain; Foundation for the Promotion of Health and Biomedical Research of the Valencian Community (FISABIO), València, Spain; Center for Networked Biomedical Research in Epidemiology and Public Health (CIBEResp), Madrid, Spain; Institute of Integrative Systems Biology (I2Sysbio), Universitat de València and Spanish Research Council (CSIC), València, Spain; Foundation for the Promotion of Health and Biomedical Research of the Valencian Community (FISABIO), València, Spain; Center for Networked Biomedical Research in Epidemiology and Public Health (CIBEResp), Madrid, Spain; School of Biological Sciences, University of Reading, Reading, United Kingdom; School of Natural Sciences, Bangor University, Bangor, Wales, United Kingdom; Institute and Museum of Natural Sciences, National University of San Juan, San Juan, Argentina

**Keywords:** Hox genes, extinction, human microbiome, genome erosion, photoreceptor, circadian rhythm

## Abstract

Most humans carry mites in the hair follicles of their skin for their entire lives. Follicular mites are the only metazoans that continuously live on humans. We propose that *Demodex folliculorum* (Acari) represents a transitional stage from a host-injuring obligate parasite to an obligate symbiont. Here, we describe the profound impact of this transition on the genome and physiology of the mite. Genome sequencing revealed that the permanent host association of *D. folliculorum* led to an extensive genome reduction through relaxed selection and genetic drift, resulting in the smallest number of protein-coding genes yet identified among panarthropods. Confocal microscopy revealed that this gene loss coincided with an extreme reduction in the number of cells. Single uninucleate muscle cells are sufficient to operate each of the three segments that form each walking leg. While it has been assumed that the reduction of the cell number in parasites starts early in development, we identified a greater total number of cells in the last developmental stage (nymph) than in the terminal adult stage, suggesting that reduction starts at the adult or ultimate stage of development. This is the first evolutionary step in an arthropod species adopting a reductive, parasitic, or endosymbiotic lifestyle. Somatic nuclei show under-replication at the diploid stage. Novel eye structures or photoreceptors as well as a unique human host melatonin-guided day/night rhythm are proposed for the first time. The loss of DNA repair genes coupled with extreme endogamy might have set this mite species on an evolutionary dead-end trajectory.

## Introduction

Over a hundred different species of follicular mites have been morphologically described from a wide variety of animals, ranging from marsupials to placentals such as armadillos, bats, pigs, dogs, rodents, and primates. In most wild animals, the mites do not cause any pathology; however, in domestic animals, particularly in dogs and cats, demodetic mange can be deadly despite the fact that the mites are also present in most healthy dogs (Ravera et al. 2013). Humans can carry two follicular mite species: *Demodex folliculorum*, which aggregates in groups in the infundibular portion of hair follicles, and *Demodex brevis*, which is a solitary species inhabiting the sebaceous glands of the skin. The mites are most numerous in the wings of the nose, on the forehead, in the ear canal, and on the nipples. Their prevalence in humans is likely above 90%, where greater numbers and, thus, easier detection of mites are associated with an older host age and larger host pores; however, the density of mites in humans peaks with sebum production between 20 and 30 years of age (Foley et al. 2021). In most humans, *D. folliculorum* (hereafter Demodex) is completely harmless, although clinical disease associated with this mite can manifest in some people (Thoemmes et al. 2014; Pormann et al. 2021). The reason why certain people show pathology in the presence of Demodex has now been unraveled in one case. Chronic demodicosis can be linked to a gain-of-function mutation in the immune response of humans against Demodex (Martinot et al. 2021; Shamriz et al. 2021).

Our hypothesis is that human-hosted Demodex is currently evolving from a parasite to an obligate ectosymbiont or obligate biotroph. The aim of this study is to show that gene loss occurred early in the evolution of Demodex toward becoming an obligate ectosymbiont or obligate biotroph. There are precedents for this, for example, the human fungus *Pneumocystis jirovecii* acquired obligate biotrophy through gene loss (Cisse et al. 2014). The hypothesized adaptation to a new life history is reflected in the transmission and infectivity of Demodex, reductions in its molecular and cellular complexity, and its morphological and physiological modifications allowing it to live on human skin.

## Results and Discussion

### Demodex is Predominantly Maternally Inherited

It is expected that a parasite is predominantly horizontally transmitted. After sequencing a fragment of the mitochondrial DNA of Demodex, the inheritance of the mites was investigated. Demodex is mainly transmitted from mother to offspring (supplementary fig. S1, Supplementary Material online). Our analysis of Demodex transmission in couples and families (supplementary fig. S1, Supplementary Material online) showed that children and grandchildren clustered only with the female lineage whereas females and males living together carried divergent lineages. The increased temperature and moisture levels at the nipple during breastfeeding facilitate the transfer of the mites from mother to offspring, reminiscent of amphiparatenic transmission. Small numbers of Demodex have also been detected on both the vulvas and penises of humans, but their roles in transmission have yet to be resolved. Birth via Cesarean section and exclusive bottle-feeding likely prevent transmission and may result in human offspring with no mites or carrying the very minimum number of founders. Wet nursing and cross-nursing will likely corrupt any inherited lineages. Our results do not exclude the possibility of occasional horizontal transmission of Demodex between unrelated people but show that transmission is currently mainly vertical. With this vertical transmission in humans, the mites no longer need to be freely contagious. The isolation of Demodex lineages has also been seen on a larger scale across geographical regions and persists even if the human host relocates elsewhere (Palopoli et al. 2015). The life cycle of Demodex from egg to adult is roughly 2 weeks long, and it is assumed that adults live for an additional week or two. Assuming an average life cycle for Demodex of 3 weeks and an average human generation time of 22–33 years translates to 382–574 mite generations before vertical transmission. Given a human life expectancy of 72.6 years, this constitutes 1,262 generations before the mites die with their host. The number of generations provides a rough idea of how much genetic variation in Demodex is to be expected between subsequent human generations and how often Demodex is exposed to bottlenecks of transmission.

Demodex mites are not the only species that are transmitted via the nipple during breastfeeding. Among parasitic animals, at least four species of flukes, a few species of cestodes, and approximately 20 species of nematodes, including roundworms, hookworms, threadworms, and lungworms, have evolved to undergo vertical transmission through breast milk in their vertebrate hosts (Lyons 1994; Shoop 1994; Chermette 2004; Foster et al. 2009; Boehm et al. 2015; Bezerra-Santos et al. 2020). Likewise, most feather lice are vertically transmitted, as are amphibious lice (Brooke 2010; Leonardi et al. 2013; de Moya et al. 2019). The same is true for feather mites (Dona et al. 2017, 2018, 2019; Matthews et al. 2018; Mironov et al. 2020). Demodex species with amphibious hosts, such as seals and sea lions, may have lost the ability to undergo horizontal transmission as well.

The vertical transmission of ectoparasitic feather lice likely started after the Cretaceous–Paleogene mass extinction event, showing that vertical transmission alone is a stable evolutionary trajectory for parasites (de Moya et al. 2019). However, vertical transmission in human-hosted Demodex might not be a stable trajectory. It is proposed that the isolation of Demodex in maternal human lineages has led to the degeneration of the genome of *D. folliculorum* to a point at which the survival of the species over evolutionary time might be in question.

### *Demodex folliculorum* Genome

The nuclear and mitochondrial genomes and transcriptomes of Demodex were sequenced, assembled, and analyzed. The total nuclear assembly length is 51.5 Mbp. It is divided into 241 scaffolds with a scaffold N50 of 488 kb and a scaffold L50 of 31. The GC content is 31.3%, and the genome encoded 9,707 proteins (supplementary tables SI 3 and 4, Supplementary Material online). Estimates of genome completeness [Bencmarking Universal Single Copy Orthologs (BUSCO)] based on arthropods showed the Demodex genome to be comprehensive (97.4% complete), with 985 complete single-copy genes, 18 complete duplicated genes, 15 fragmented genes, and 48 missing genes (supplementary fig. SI 1, Supplementary Material online), strengthening the genome analysis. Among the 9,707 proteins, 8,131 could be assigned to orthogroups. Only six orthogroups representing 46 genes were Demodex specific; see supplementary table SI 7, Supplementary Material online for comparison. A rough estimate placed the number of pseudogenes at just above 100, corresponding to approximately 1.1% of coding genes; see supplementary figure SI 2 and table SI 1, Supplementary Material online. The mean intron length was 514 bp (median 79 bp), the mean intron count per gene was 2.83, the mean exon length was 382 bp (median 180 bp), and the mean distance between coding sequences was 2,429 bp (median 1,293 bp). See supplementary table SI 6, Supplementary Material online for comparison. One hundred and sixty-four (164) repeat families were represented in the genome, accounting for 7.2% of the genome (supplementary table SI 5, Supplementary Material online).

To enable the highest quality of the genome assembly, it is imperative that the Demodex used for sequencing originate form a single host, from a single lineage of mites. No information will be available about differences in the genome between populations like singl-nucleotide variants, indels, and structural variations including copy number variants (gene dosage effects), larger deletions and insertions, duplications, and rearrangements such as inversions, intrachromosomal and interchromosomal translocations. These structural variations have in humans an up to 53 times greater effect on gene expression than single nucleotide variants and indels (Chiang et al. 2017). It is now estimated that each human genome differs by >20,000 structural variations (Ho et al. 2020). Applied to Demodex, this could mean differences by >337 structural variations. Considering that we used some 250 mites for DNA sequencing, around 85,000 structural variants would not have allowed the genome assembly we have achieved. Given a genome size of only 51.5 Mbp, the expected number of structural variations should be much lower due to extensive inbreeding and a reduced number of copy number variants, see also supplementary figure SI 1, Supplementary Material online (BUSCO assessment).

In our work, we argue that relaxed selection and genetic drift resulted in the very small genome of Demodex. By basing our analysis on just one genome, we might misinterpret recent effects of directional selection and adaptation in a particular environment or geographic region as genetic drift (Coop et al. 2009; Hollox et al. 2022; Saitou et al. 2022).

For the human genome it is assumed that a large-scale increase in copy number of receptor genes associated with taste and smell was possible through relaxation from negative selection and ensuing neutral evolution (Nguyen et al. 2008; Hollox et al. 2022). In Demodex, the opposite is the case.

Sequencing the genome of more Demodex lineages might reveal evidence on one side on sexual isolation (Zhang et al. 2021) or, on the other side, on admixture and introgression (Quan et al. 2021).

The Demodex mitochondrial genome is 14,164 bp long with a GC content of 30.0% and encodes 13 proteins, 2 rRNAs, and 22 tRNAs (Palopoli et al. 2014). The nucleotide composition [A 6,209 (43.8%), C 3,072 (21.7%), G 1,178 (8.3%), and T 3,705 (26.2%)] does not follow Chargaff’s parity laws (Fariselli et al. 2021; Forsdyke 2021). We identified three primary polycistronic transcripts that were posteriorly processed; this is the first report of cistrons in Acari (supplementary fig. S3, Supplementary Material online). The existence of three polycistrons is likely the ancestral state for arthropods (Francoso et al. 2020).

### Demodex has the Lowest Number of Protein-Coding Genes Identified in Arthropods

The evolution of the genomic repertoire of free-living species is expected to include cycles of genome expansion due to (partial) genome duplications, with rapidly increasing complexity, followed by longer periods guided by natural selection for efficient resource utilization (streamlining) or random genetic drift resulting from environmental or demographic change, leading to genome reduction (Wolf and Koonin 2013; Fernandez and Gabaldon 2020; Guijarro-Clarke et al. 2020). Increasing parasitic associations further reduce the need for coding genes with at least one exception; the genomes of horizontally transmitted microspordian parasites are smaller than those of a mixed, horizontally and vertically transmitted parasite species in the same host subject to nonadaptive processes (Haag et al. 2020).

A change in the transmission mode from horizontal to vertical might lead to a decrease in the effective population size, which will lead to less effective selection in free-living animals (Yongzhen et al. 2018; Wilkins 2019). Less effective selection will trigger an increase in transposable elements and genome size (Lefebure et al. 2017; Chen et al. 2020; de Albuquerque et al. 2020) until the parasite/symbiont association becomes so close that transposable elements disappear again, and genome size and gene numbers show the greatest decreases.

Demodex exhibits the smallest number of coding genes (9,707) identified to date and the second smallest genome (51.6 Mbp) among panarthropods (supplementary table SI 3, Supplementary Material online). The smallest panarthropod genome of 32.5 Mbp belongs to the tomato russet mite, *Aculops lycopersici* (Greenhalgh et al. 2020), a plant parasite. Demodex and Aculops also present the smallest sequenced genomes among most invertebrate groups outside of the panarthropods. Lineages at the base of the metazoan tree have larger minimal genomes (e.g., Porifera, *Amphimedon queenslandica*, 166.7 Mbp; Placozoa, *Trichoplax* sp. H2, 94.9 Mbp; Ctenophora, *Mnemiopsis leidyi*, sea walnut, 155.9 Mbp; and Xenacoelomorpha, *Hofstenia miamia*, three-banded panther worm, 949.9 Mbp). With only one exception, all genomes smaller than that of Demodex belong to highly parasitic species, among which the smallest belong to the orthonectidian annelid *Intoshia variabili*, a parasite of flatworms (15.3 Mbp); *Intoshia linei*, a parasite of ribbonworms (41.6 Mbp); and the myxozoan cnidarian *Kudoa iwatai*, a parasite of fish (31.2 Mbp). Exceptions are observed in nematodes (e.g., *Rhabditophanes* sp. KR3021 is a free-living nematode with a contig-based genome assembly of 47.3 Mbp), and many other parasitic nematodes have smaller sequenced genomes, among which that of the banana root nematode, *Pratylenchus coffeae*, is the smallest (38.2 Mbp).

While parasitism has been shown to reduce genome size in most animal lineages, the intensity of host–parasite interaction and dependency is decisive. This situation is very pronounced in Acari. Acari species are divided into Acariformes and Parasitiformes. In Parasitiformes, all ticks, Ixodida, are blood-sucking parasites that exhibit the largest genomes of all Acari. While the ticks are parasitic, they often exhibit more than one host species and spend considerable time off of their host. However, Demodex no longer leaves its host. Among mite genome sizes, those of house dust mites stand out; the small genome sizes of these species are proposed to be the result of a bottleneck, as they are reportedly derived from bird-parasitic lineages, representing a rare transition from a parasitic lifestyle back to a free-living lifestyle (Klimov and OConnor 2013; Xu et al. 2016).

### The Demodex Genome is Eroding

Genome size reduction in animal parasites is driven by gene loss, the disappearance of repetitive elements, and reductions in intergenic regions and intron length (supplementary tables S1 and SI 6, Supplementary Material online) (Slyusarev et al. 2020). The last prediction does not hold for the mite *A. lycopersici*, which exhibits the smallest identified genome but a median intron length more than twice that of Demodex or *Sarcoptes scabiei*. We propose that *A. lycopersici* is still undergoing a transition from herbivore to plant parasite (Grbić et al. 2011; Fellous et al. 2014; Martel et al. 2015).

The load of transposable elements correlates with genome size in arthropods (Wu and Lu 2019). In our analysis, the reduced genomes of Acariforme mites show a notable dearth of repetitive elements, presenting the smallest number among arthropods. Both the proportion of repeats in the genome and the diversity of repeats (number of repeat families) are reduced in Acariformes relative to other arachnids and wider outgroups, with total repeats ranging from 7% of the smallest *D. folliculorum* genome to 12% of the *Tetranychus urticae* genome (supplementary table SI 5, Supplementary Material online). Larger genomes tend to harbor more repeats in Parasitiformes; for example, the *Ixodes scapularis* genome contains the most repeat families and shows the highest proportion of repeats (41%) among the surveyed mites and ticks; this is also true for the largest Acariforme genome, belonging to *T. urticae*, which is comparable in repeat content to the genome of the Parasitiforme *Galendromus occidentalis*. Interestingly, the *Drosophila melanogaster* genome shows a higher diversity and density of repeats than those of arachnids with comparable genome sizes. The reduced repeat numbers in smaller Acariforme genomes may be explained by increased mutation rates, genetic drift, and silencing by plant-like short interfering RNAs (Li and Gu 2018). Demodex shows reductions in both coding and noncoding regions. The average distance between annotated coding sequences is shorter in all Acariformes species than in other arachnids and Drosophila, as is the average intron length (supplementary tables SI 5 and 6, Supplementary Material online).

Codon usage bias can indicate the degree to which natural selection has acted on a set of sequences. We found the number of effective codons (Nc) to be smallest in the four Acariformes species examined (supplementary fig. SI 4, Supplementary Material online). After correcting for background mutations, this bias was reduced, and similar levels were observed across all species studied. When codon use was compared against the genome-wide GC content, Nc was found to be positively correlated whereas corrected Nc was not. Thus, the codon usage bias seen here was less likely to be due to codon preference (selection) and more likely to be due to mutational bias leading to a higher frequency of codons containing adenine or thymine at the second and/or third position; an exception was observed in *Dermatophagoides farinae,* which showed strong gene-wide codon bias regardless of background mutation correction.

**Fig. 1.**
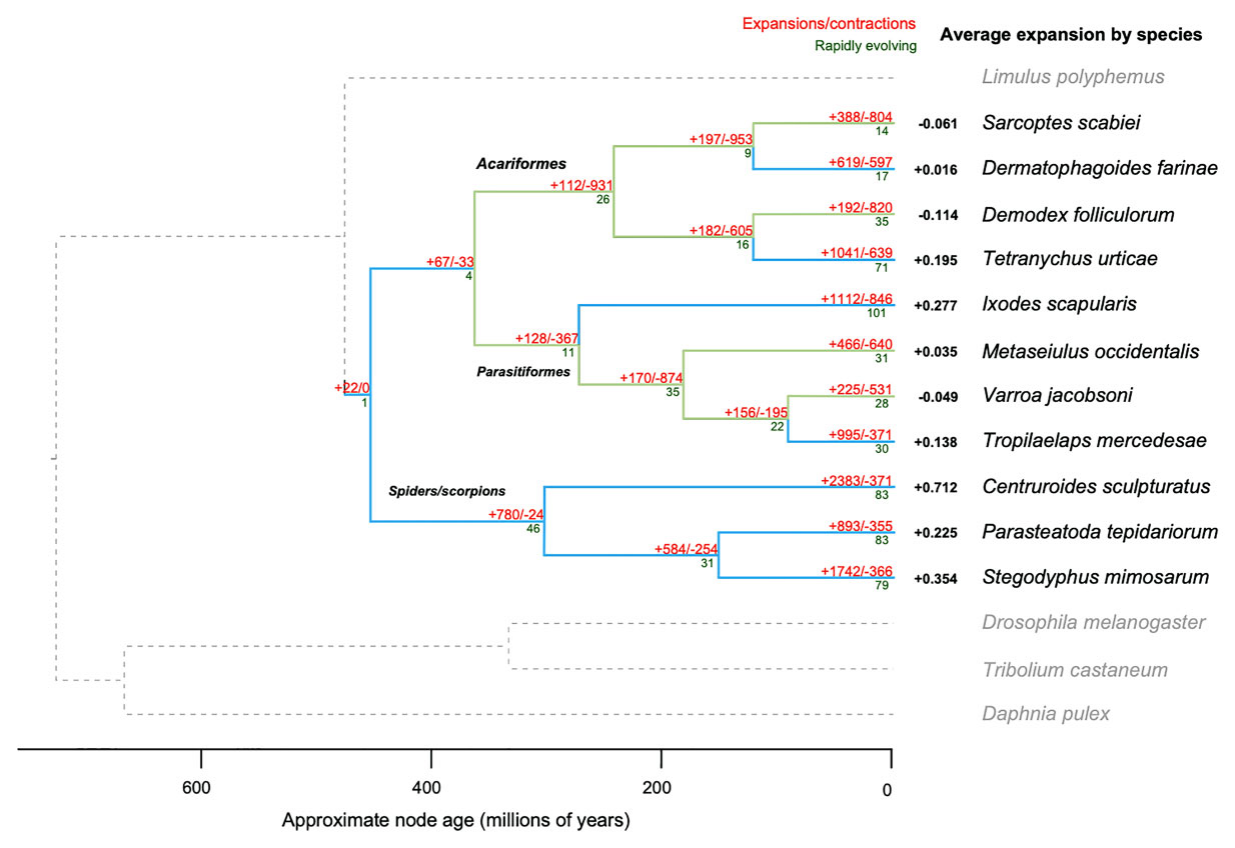
Gene family size evolution in arachnids showing gene loss in Demodex. Numbers show how many gene families have expanded (+) and contracted (–; red), number of rapidly evolving families (dark green; *P* < 0.05), and average expansion by species (black). Branches are highlighted to show those with net contractions (light green) and expansions (light blue). Alternative IQtree topologies have been explored, supplementary figure SI 7, Supplementary Material online. The color indicates the number of genes annotated with each term.

**Fig. 2.**
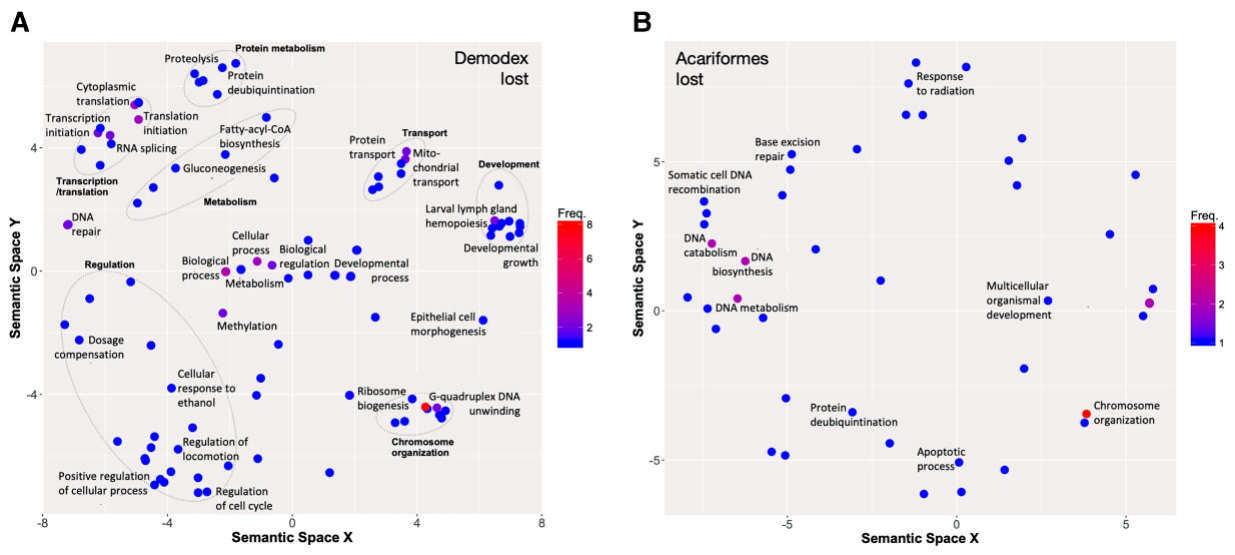
Functions of lost gene families in Demodex (*A*) and Acariformes (*B*). Gene Ontology (GO) terms are presented, residing in semantic space, for genes identified as having enriched functions in Demodex (*FDR* < 0.1), and those annotated with “DNA repair” in Acariformes (enrichment tests did not produce significant terms). GO terms were filtered to remove redundant terms, and grouped by broad similarity (dashed lines, named in bold). Functions of rapidly evolving gene families, contracting and expanding, for Demodex and Acariformes are shown in supplementary figure S2, Supplementary Material online.

To examine the changes in gene family size during the evolution of Demodex, the orthology of genes across 15 species of arachnids and outgroups was determined, and a total of 14,072 orthogroups were identified (supplementary table SI 4, Supplementary Material online). Demodex presented an assignment rate of 84%, with only 1,576 genes not being allocated to any orthogroup. The average expansion of gene families across the tree was only negative in Demodex, *S. scabiei* (scabies mite), and *Varroa jacobonsi* (bee mite) plus *A. lycopersici* (fig. 1). Demodex presented the strongest signal of average reduction in gene family size, along with the lowest number of genes and smallest proteome among the 15 species, including *A. lycopersici*. The greatest gene loss was observed in the same three species, which are intimately associated with a single host throughout their life cycle. The branch leading to Demodex presented gene losses in a total of 820 gene families, with a 4.3-fold more losses than gains, and 28 gene families showed rapid contraction, while seven showed significant expansion. Demodex presented a complete loss of functionality of 236 gene families (no orthologs) relative to the other Acariformes (fig. 2, supplementary table SI 12, Supplementary Material online).

The last common ancestor (LCA) of Acariformes exhibited signals of overall gene loss, suggesting that genome reduction might have occurred early in the group, before the extant species originated. It should be noted that the last common ancestor in our phylogeny may not represent the true LCA of Acariformes, as many species not included in our study likely have larger (and less AT-biased) genomes. However, these data do suggest that substantial gene loss was common early in the evolution of small-genome Acariformes and that subsequent changes in gene family size (whether losses, gains, or little change) occurred later in a more species-specific manner. For example, gene loss continued in *D. folliculorum* living on a human host, whereas losses and gains were more balanced for *D. farinae*.

The assessment of gene family size changes in Parasitiformes also revealed signals of gene loss, followed by species-specific changes. For example, in our analysis, *V. jacobsoni* exhibited overall gene loss, whereas the predatory mite *G. occidentalis* presented more balanced gains and losses. Spider and scorpion species showed large increases in gene family size throughout their history, mirroring previous findings regarding spider genome size evolution, suggesting that ancestral whole-genome duplication events have driven increases in genome size in this group (Schwager et al. 2017). Furthermore, our data suggest that gene family expansions continued in a species-specific manner, in agreement with observations of later lineage-specific tandem duplications in these groups (Schwager et al. 2017). Overall, the greatest gene loss was observed for those species intimately associated with a single host throughout their life cycle (i.e., *D. folliculorum*, *S. scabeii*, and *V. jacobsoni*).

Gene family evolution analysis identified families with significant rapid evolutionary change (*P* value <0.05). In *D. folliculorum,* a total of 28 gene families presented rapid contraction (supplementary fig. S2 and SI 6, Supplementary Material online). Functional enrichment using the consensus functions of gene families indicated four significant slim GO terms: “Reproduction”, “Response to stress”, “Immune system process” and “Lipid metabolic process” (*FDR* < 0.1, supplementary table SI 9, Supplementary Material online). The Kyoto Encyclopedia of Genes and Genomes (KEGG) orthology annotations included six genes associated with the lysosome, an organelle that plays a role in digesting molecules in cells in a range of different biological contexts, including lipid metabolism, reproduction, and the immune response. More specific (non-slim) GO terms included “Multicellular organism reproduction”, broad categories of metabolic classes, and several terms related to responses to stimuli. The gene families annotated with “Reproduction” included three subfamilies of cathepsins (B, L, and K; K01363, K01365, and K01371, respectively), lipases (including lysosomal acid lipase, K01052), acetylcholinesterases, and proteases. Acetylcholinesterases are present in tick saliva, and the encoding gene families are expanded in tick species (Kim, Tirloni, et al. 2016); these enzymes could play a role in host–parasite interactions through the immune system. The functional term “Reproduction” appeared to link several lysosomal genes, suggesting associations between a reduction in lysosomal complexity involved in metabolism and/or immunity and reproductive processes. Interestingly, one significantly contracting group annotated with “Reproduction” was the meiosis arrest female 1 (*MARF1*) group, whose members play a key role in meiosis control and the regulation of transposable elements in the oocyte. The contraction of this gene family may be related to the reduced occurrence of transposable elements in the Demodex genome. Only seven gene families showed significant expansion in Demodex, and thus, no enrichment test was performed (supplementary table SI 2, Supplementary Material online).

The examination of rapidly evolving gene families on Acariformes branches showed gene loss, revealing 49 families with significant contractions and 31 with significant expansions (supplementary fig. S2, supplementary tables SI 10–13, Supplementary Material online). Enrichment tests showed contraction to be associated with two significant GO terms: “Reproduction” and “Locomotion”. More specific GO terms included several developmental genes functioning in the “Determination of adult life span” and “Metamorphosis”, “Digestion”, and “Sperm storage”. The contracting developmental gene families identified in Acariformes included the ANTP class of homeobox genes, a large cluster including the canonical *Hox* genes, *ParaHox* genes, and *SuperHox* genes, among others (Ferrier 2016). Nine and 15 gene families were annotated with “Reproduction” and “Locomotion”, respectively, 6 of which were annotated with both terms. Fecundity and locomotion are often interlinked via metabolism and fitness trade-offs, and these six overlapping genes presented specific functions related to lipid and protein metabolism and development. Rapid expansion was associated with five significant terms: “Cell differentiation”, “Cellular developmental process”, “Anatomical structure development”, “Developmental process”, and “Lipid metabolic process”. The specific terms included metabolic processes related to carbohydrates, steroids, lactate, fatty acids, and amino acids and oxidation–reduction processes.

To examine the loss of function in reduced genomes, the loss of gene families was examined in both Demodex, to identify human follicle mite-specific loss, and Acariformes more generally. In total, 236 gene families showed no orthologs in Demodex but were present in every other evaluated Acariforme species. These genes were enriched for 19 GO slim terms, broadly describing components of the ribosome involved in ribosome biogenesis, gene expression through the translation term, and cell morphogenesis. More specific terms included functions related to transcription and translation, particularly RNA splicing and DNA/protein modifications (e.g., methylation and protein ubiquitination), and DNA repair. The identified developmental terms included “Developmental growth”, “Epithelial cell morphogenesis”, and “Larval lymph gland hemopoiesis”.

Acariformes presented the loss of 271 gene families when relative to every other evaluated arachnid species. Functional enrichment did not identify any significantly overrepresented functions in this set of genes. However, DNA metabolism-related terms were most significant terms in the list (*P* < 0.05); thus, genes with these GO terms were explored to identify more specific functions, including chromosome organization-related GO terms, DNA recombination, and repair.

### Relaxed Selection as a Mechanism of Genome Reduction

**Fig. 3.**
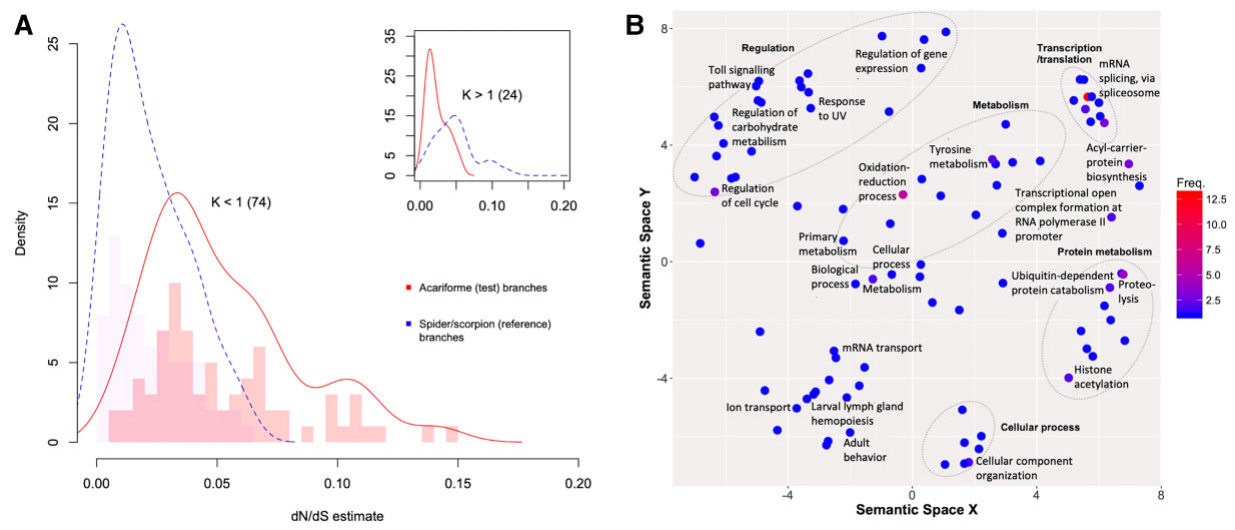
Relaxed selection is more common than intensified (purifying) selection in Acariformes compared with spiders and scorpions. (*A*) Density plot of omega values (nonsynonymous/synonymous substitutions: dN/dS) for genes showing relaxation of selection (*K* < 1; main) and intensified selection (*K* > 1; inset) in Acariformes. Number of genes in brackets. (*B*) Functions of genes under relaxed selection in Acariformes. Details are as per figure 2. For the distribution of the RELAX parameter *K* across significant selection tests (adjusted *P* < 0.1; red bars), and all tests (blue bars, inset), see supplementary figure S4, Supplementary Material online.

Relaxed selection either impels evolutionary innovation or presages a loss of function and lineage extinction (Wertheim et al. 2015). Selection was observed to have more frequently relaxed than intensified during Acariformes evolution (fig. 3). The RELAX K values for significant groups were skewed toward relaxed selection (*K* < 1) relative to all 467 K values, with 75% of genes showing relaxed selection on branches where gene loss had occurred (83 and 74 for *P* value <0.05, and adjusted *P* values <0.1, respectively); supplementary figure S4, supplementary table SI 14, Supplementary Material online. This suggests that selection has more frequently relaxed than intensified during Acariformes evolution relative to species experiencing gene family expansions, possibly playing a role in genome reduction through the loss of gene content.

Biochemical and physiological functions of genes under relaxed selection might be altered or lost. The functional enrichment analysis of the 74 relaxed genes revealed 21 significant GO terms, supplementary tables SI 15 and 16, Supplementary Material online. These included functions related to mRNA processing and protein modification and primary metabolism involving nucleic, amino, and carboxylic acids. The more specific functions of these gene families included alternative splicing via the spliceosome, protein modifications such as ubiquitination and acetylation, and regulatory terms including the regulation of gene expression and the cell cycle. The 24 genes showing a significant intensification of selection (adjusted *P* value <0.1, *K* > 1) were not significantly enriched for any functional term.

Relaxed selection can play a role in gene loss, so we may expect that the functions of genes showing signals of weakened selection, gene families that have undergone rapid contraction, and genes that have been lost will present some degree of functional overlap. The gene ontology terms with significant enrichment in each group showed broad overlap of terms related to metabolism, although the specific class of metabolism varied. Among the contracted gene families, lipid metabolism was more prominent, and among lost genes and those under relaxed selection, the associated metabolic terms were more frequently related to nucleic and amino acids, although the relaxed genes also presented terms related to other organic acids. The functional overlap was greater between lost genes and those under relaxed selection, suggesting a link between the two. In particular, both groups were enriched in terms related to gene expression, notably including ribosome biogenesis (10 lost and 4 relaxed genes), spliceosome structure (4 lost and 11 relaxed), protein modifications, and mRNA processing. This suggests that relaxed selection and genome reduction may involve a reduction in the complexity of the molecular machinery functioning in genome expression (e.g., ribosomal proteins) and a decrease in chemical modifications introduced during transcription and translation. Similar patterns of molecular machinery loss have been revealed in bacterial endosymbionts (e.g., *Serratia symbiotica*), showing convergent evolution in prokaryotic and eukaryotic systems (Manzano-Marín and Latorre 2016). This pathway is an alternative to that identified in free-living species that undergo genome streamlining based on a high growth rate in nutritionally limited environments (Lamichhaney et al. 2021).

Purifying selection contributes to genome reductions in prokaryotic species (Williams and Wernegreen 2012; Albalat and Cañestro 2016; Valadez-Cano et al. 2017); it is observed in endosymbionts that confer an advantage to their host and in organellogenesis (Uthanumallian et al. 2022). No endosymbiotic eukaryotic animals have been identified to date. In parasitic associations, purifying selection (to the extent that it is detectable) is limited to host interaction. In Demodex and socially parasitic ants, the effect of recombination in counteracting Muller’s ratchet is severely reduced due to inbreeding (Schrader et al. 2021).

### AT Bias and Mode of Living

Acariformes and Demodex presented genome-wide patterns of AT bias (supplementary fig. S5, Supplementary Material online). Demodex showed a genome-wide GC content of 31.3%. There are numerous selection pressures that contribute to the evolution of the genome-wide GC content, which varies widely across the domains of life. The GC content within a genome can vary from region to region, between coding and noncoding regions, and even between genes (Wang 2018; Browne et al. 2020; Pracana et al. 2020; Adachi et al. 2021). Generally, Acariformes species show stronger AT bias in GC2 than in GC3 and genome wide relative to other arachnids, suggesting mutational biases toward AT in these species that selection has not corrected (supplementary fig. SI 5, Supplementary Material online).

The examination of a single gene (*Srp54K*) by Klimov and OConnor (2013) suggested that AT bias is common in Acariformes and is correlated with living mode, with host-associated species presenting increased AT bias in the gene. Furthermore, both AT bias and host association appeared to be more derived traits on a tree of wider Acariformes species. Although this analysis involved only a single gene and multiple selection pressures shape GC content, these results suggest dynamic changes in genome size and composition across Acariformes, dependent on the mode of living.

In particular, the loss of mismatch repair genes has been observed to have a large effect on GC content in the genomes of endosymbiotic bacteria (e.g., *MutY*, *vsr*, and *ndk*) (Acosta et al. 2015). Similar to the prokaryotic world, three mismatch repair genes (*MutY*, *SMUG1*, and *TDG*) appear to have been lost in Demodex relative to other arachnid species as have genes known to interact with them. *MutY* is an adenine DNA glycosylase responsible for the detection and removal of adenine mutations, typically resulting from oxidative damage; if such mutations are left unrepaired, they will lead to CG to AT transversions (Parker and Eshleman 2003). *SMUG1* is a single-strand selective monofunctional uracil glycosylase that removes uracil from single- and double-stranded DNA. *TDG* is a G/T mismatch-specific thymine DNA glycosylase that primarily removes G/T mismatches, although it can also remove thymine from C/T and T/T mispairings. In addition to these DNA repair genes, several other genes are thought to play a role by interacting with *SMUG1* in the context of carcinogenesis, including *BRCA1*, *ATM*, and *XRCC1*. Demodex has lost similar genes, including *BARD1* (*BRCA1*-associated gene), *ATM*, and *XRCC3*.

The presence of *MutY*, *SMUG1*, and *TDG* varies across arachnid species; however, neither *S. scabiei* nor Demodex genome appears to harbor these genes, and *T. urticae* and *D. farinae* each harbor only one of these genes (*TDG* and *MutY*, respectively). This suggests that, like many endosymbiotic bacteria, *D. folliculorum* (and other Acariformes) may present an underlying AT-biased mutation pattern across the genome. To test whether such a mutational bias exists in the Demodex genome, the spectrum of mutation types was examined using RNA-sequencing data. By far the most common mutation type observed in *D. folliculorum* was transversions leading to A or T mutations (supplementary figs SI 3 and 5, Supplementary Material online). Transitions were almost equally balanced in frequency, and transversions resulting in a C or G mutation presented a very low frequency, comprising only 8% of mutations. These data strongly suggest that there is an AT mutation bias in Demodex and that this bias is most likely caused by the loss of DNA repair genes that specifically target the removal of adenine and thymine residues. A second mechanism that contributes to AT bias is the frequent methylation of cytosines, which are subsequently replaced with thymine (Hershberg and Petrov 2010; Mathers et al. 2019). With the loss of DNA methylation in Demodex, this mechanism should no longer contribute to increasing AT bias.

### Deviation from the Canonical Order of *Hox* Genes

**Fig. 4.**
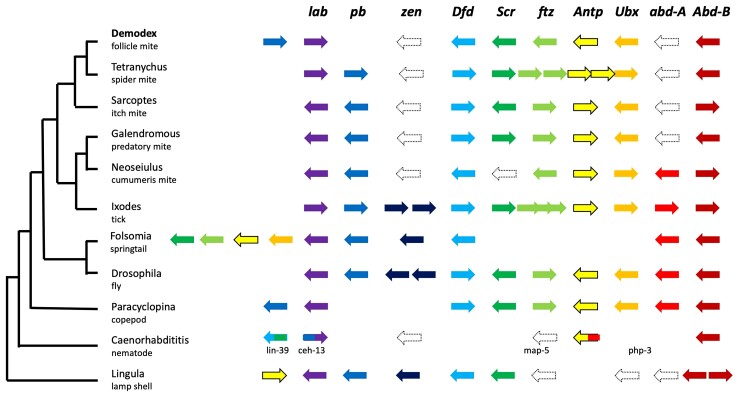
The canonical order of *Hox* genes has only been breached in very few cases for bilateral animals. Demodex, Paracyclopina, and nematodes position *pb* upstream of *lab*. Folsomia and Lingula have central *Hox* genes in front of the anterior *Hox* genes, and a sea urchin has central Hox genes at the most posterior position. Punctuated outlines indicate loss of genes, whereas empty spaces suggest relocation of genes. The mite *Aculops lycopersici* shows the canonical order with no duplications. Abbreviations: *lab*: *labia, Hox1l*; *pb*: *proboscipedia, Hox2*; *zen*: *zerknüllt or zerknüllt-2*, *Hox3*; *Dfd*: *Deformed*, *Hox4*; *Scr*: *Sex combs reduced, Hox5*; *ftz*: *fushi tarazu, Hox6*; *Antp*: *Antennapedia*; *Ubx*: *Ultrabithorax*; *abdA*: *Abdominal-A*; *AbdB*: *Abdominal-B*; *Demodex*: *D. folliculorum* (Acariformes); *Tetranychus*: *T. urticae* (Acariformes); *Galendromus*: *G. occidentalis* (Parasitiformes); *Neoseiulus*: *N. cucumeris* (Parasitiformes); *Ixodes*: *I. scapularis* (Parasitiformes); *Folsomia*: *F. candida* (Entognatha), *Drosophila*: *D. melanogaster* (Insecta); *Paracyclopina*: *P. nana* (Crustacea); *Caenorhabditis*: *C. elegans* (Nematoda); *Lingula*: *L. anatina* (Brachiopoda).

The canonical order of *Hox* genes on a chromosome and their spatial expression along the anteroposterior body axis have rarely been observed to deviate from those in bilateral animals. Even if the *Hox* genes are atomized in individual genomic segments, as observed in the tunicate *Oikopleura dioica* or the predatory mite *Metaseiulus occidentalis*, the order of their expression is maintained (Seo et al. 2004; Hoy et al. 2016; Gaunt 2018; DeBiasse et al. 2020). In vertebrates, no deviation in the *Hox* gene order is known. In Demodex, *Labia* (*Hox2*) has moved upstream of *proboscipedia* (*Hox1*), which is a feature shared with the copepod *Paracyclopina nana* and nematodes. Mites in the Acariformes and most Parasitiformes lineages have lost *zerknüllt* and *Abdominal-A*, and Demodex is the only Acariformes known to have lost *NKX6.1* and *MEOX1*, which participate in body segmentation (Bayrakli et al. 2013; Mohamed et al. 2013). The loss of *MEOX1* alongside *AbdA* may explain the observed lack of segmentation. *T. urticae*, *M. occidentalis,* and *I. scapularis* present the standard orientation of *lab* and *pb*; therefore, no modification of the regulatory transcription of these two genes has been observed. The inverse orientation within Demodex *Hox* genes, resulting in *pb* (second-segment identity) > *lab* (first-segment identity), might have allowed adaptation to the skin follicle. The inverted locations of the two *Hox* genes (fig. 4) cause changes in transcriptional times and not morphology. In Acari, the cheliceral segment is considered homologous to the 1st antennal segment in insects (expressing *lab*), and the pedipalpal is homologous to the second antennal or intercalary segment, expressing *pb* (Telford and Thomas 1998). Follicular mites show an extreme reduction of the chelicerae in combination with more developed (protruding) pedipalps, especially in nymphal stages. Palps are crucial for finding and gathering food. The inversion of transcription times favors faster development of the palps than the chelicerae, thus reducing the time of a fragile developmental stage required to find food.

**Fig. 5.**
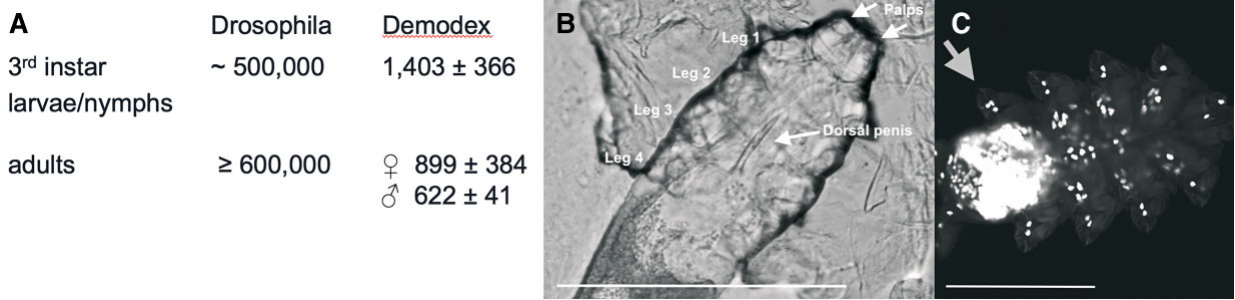
(*A*) Estimates for the total number of cells based on counting nuclei (± standard deviation, *n* = 4) with confocal microscopy. (*B*) Male Demodex dorsal view. Central arrow indicates erect penis pointing forward. Light micrograph, bar 100 µm. (*C*) Demodex, dorsal view, fluorescent confocal micrograph. Nuclei stained with DAPI. Arrow points to three nuclei in Leg 4. Part of the brain is around and below legs 4, see figure 6*A* and *B*. Bar: 50 µm.

The lack of *abd-A* in Acariformes (Tetranychus and Demodex) underlies morphological modifications of the posterior part of the body. In arthropods, the altered regulation of *Abd-B* played a role in the evolution of the abdomen (Akam et al. 1994). During development, the expression of *Abd-B* extends anteriorly (A/P axis) when *abd-A* expression ceases. If *Abd-B* is knocked down, its expression shifts anteriorly; therefore, *Abd-B* regularly inhibits *abd-A* activity in the anterior direction (Aspiras et al. 2011). In Demodex, the presence of *Abd-B* in its correct orientation, concurrent with the absence of *abd-A,* is consistent with a very anterior shift in the position of genitalia (in adults) (fig. 5*B*). The lack of *abd-A* in the presence of *Abd-B* in Demodex might play a role in the dorsal position of the penis on the prosoma, directed anteriorly, which is a unique phenotype of Demodecidae and parasitic Psorergatidae (Ah et al. 1973; Giesen 1990).

### Demodex Shows the Lowest Number of Cells Identified Among Arthropods

When free-living animals become endoparasites, they reduce the number of cells in their body (Neves et al. 2009; Isaeva and Rozhnov 2021). Here, we compare Demodex with a free-living species of the most numerous clades of arthropods, the insects. Using confocal microscopy, we counted the number of nuclei in Demodex and *D. melanogaster*. The number of cells in Demodex is reduced by more than 500-fold relative to that in Drosophila (fig. 5, supplementary table SI 17, Supplementary Material online).

In arthropods, the adult stage, the imago, always presents the largest number of somatic cells. In holometabolous insects such as Drosophila, immature cells are replaced by mature cells during the pupal stage; in other arthropod species, such as Demodex, no replacement takes place. As the first indication of an arthropod species transitioning towards a reductive parasitic lifestyle, Demodex presents a greater number of cells in the last development stage (nymph) than in the adult stage. The evolutionary cell number reduction starts in the final stage, not during early development. While an extreme reduction in the total number of cells has been observed in parasitic animals such as dicyemids, the initial period of this evolutionary path is presented here for the first time.

Demodex shows under-replication in diploid nuclei of the gnathosomal and podosomal regions (supplementary fig. S6, Supplementary Material online), a phenomenon that is only known from true flies (Diptera) (Hjelmen et al. 2020).

Cell size is not uniform in Demodex, and cells in its short legs are particularly large, a phenotype linked to gene loss. A set of 21 genes that have been lost in Demodex were annotated with “Cell morphogenesis”, which is related to the simplicity of cell morphology and leg development in Demodex. “Epithelial cell morphogenesis” was associated with *Enabled*, which influences cell shape and tissue morphogenesis (Gates et al. 2009), and some genes were annotated with neurological developmental and behavioral roles (e.g., *PPT* was associated with roles in grooming behavior and adult locomotion). The extreme reduction in the number of segments (podomeres) in the legs of Acari is unusual and was first observed in Demodex (Mégnin 1877). The walking legs of Demodex adults and nymphs are composed of three mobile segments and one fixed segment, coxa (= epimeral plate), and the palps have two mobile segments and a reduced coxa. Among the other members of Acari, both adults and nymphs have legs with up to seven podomeres, and six different muscles move each podomere (Evans 1992). In contrast, Demodex leg podomeres each have a large, single, uninucleate segmental muscle cell (fig. 5*C*). The reduction of a muscle to a single cell has also been observed in insects (Klowden 2013). The 15-μm-long miniaturized legs powered by three uninucleate muscle cells are able to walk over human skin at an average speed of 12 mm/h (Norn 1970). The three-celled legs of demodecids are the epitome of maximum size in somatic cells that no longer divide.

**Fig. 6.**
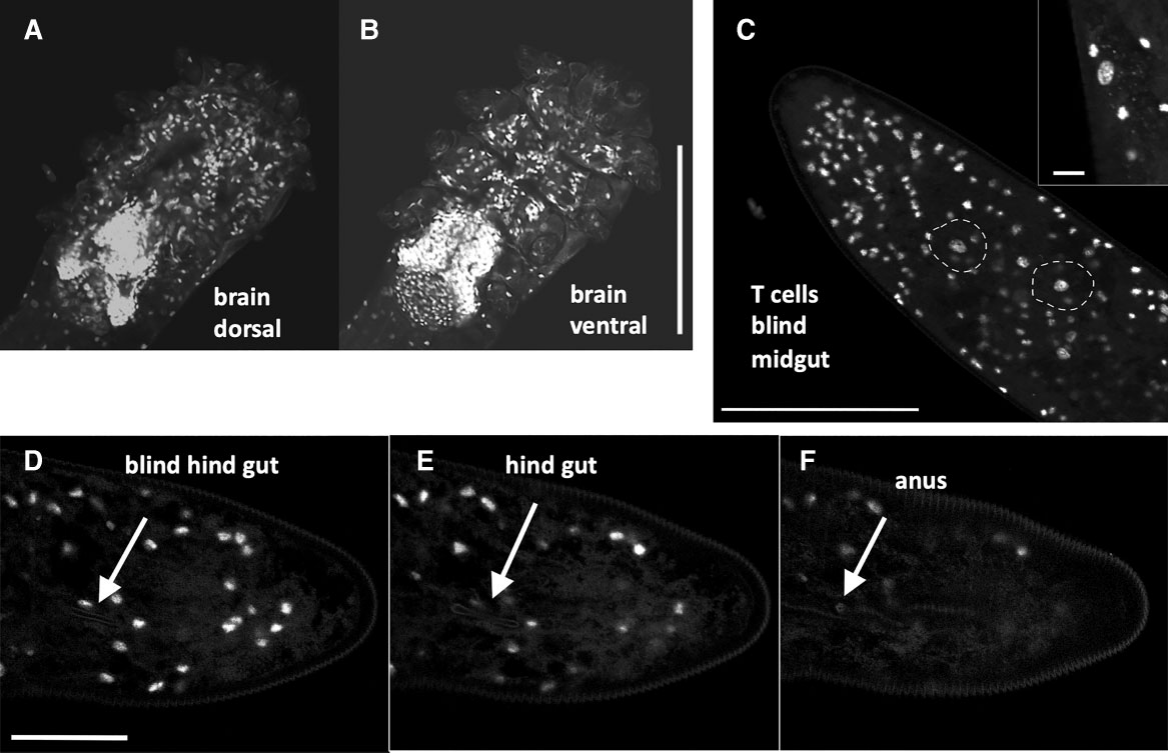
(*A* and *B*) Demodex. Despite an extremely reduced number of cells, see figure 5*B* for three nuclei inside a leg, the number of brain cells is dominant. Bar: 50 µm. (*C*) Two large nuclei of T cells involved in digestion at the end of the blind mid gut are circled. Inset, magnified view of large T-cell nucleus and mitochondrial cell signal. Bar: 50 µm, inset 5 µm. (*D*) Start of the blind hind gut tube just below nucleus. (*E*) Thin hind gut tube below nucleus. (*F*) Hind gut opening in anus. Bar: 25 µm. Fluorescent confocal micrographs, DAPI stain of DNA.

In Demodex, all tissues except for the brain present a reduction in the number of cells. Its brain is the simplest observed in Acari but occupies a large volume in relation to the total body size, indicating a miniaturization process, Haller’s rule, figure 6*A* and *B* (Beutel et al. 2005; Polilov 2015).

Prostigmata mites have an incomplete gut (no connection between the midgut and hindgut), and Demodex mites lack a proper midgut as well. Digestion is performed by disproportionately large, 40 μm uninucleate cells (Type II), carrying mitochondria of up to 3 µm (Desch 1989) (fig. 6*C*). The hindgut has been reduced to a finger-like, minute tube that opens to the outside at approximately one-third of the posterior terminus (Desch et al. 1970) (fig. 6*D*–*F*). There have been several reports that Demodex does not have an anus, and when Demodex dies, the accumulated waste spills into the pores of the skin and leads to inflammation; this is not correct (Lacey et al. 2011, 2016; Cossins 2016; Moran et al. 2017; Pormann et al. 2021).

### Photoreception and Day and Night Rhythm in Demodex

**Fig. 7.**
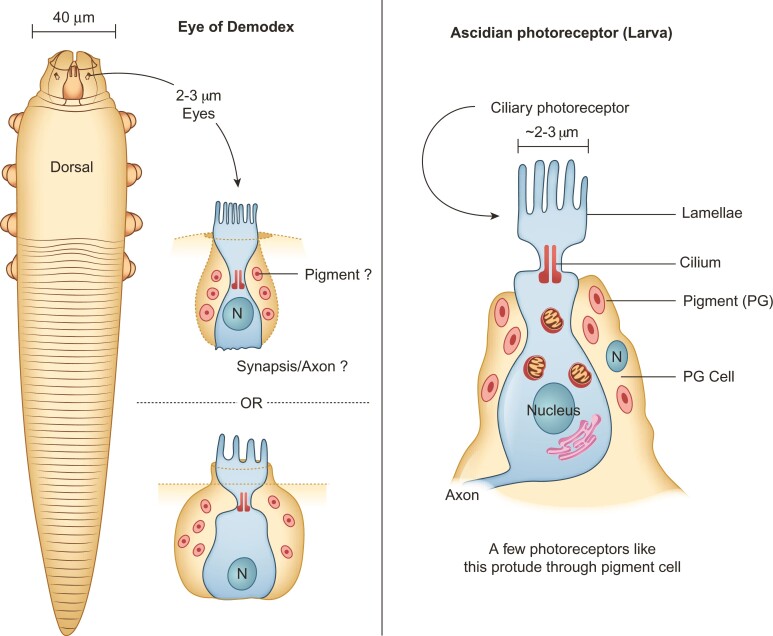
Schemata comparing Demodex eye with the basic photoreceptor of ascidians. Left panel: dorsal localization of the eyes (supracoxal spines) on a mite specimen, at its anterior end, and proposed morphology of the photosensitive organs. Right panel: schema of the ascidian photosensitive organs, adapted from Lamb (2013).

Chelicerates lack compound eyes, but some species have ocelli or eye spots. Demodex has a pair of dorsoanterior photosensitive organs known as “supracoxal spines” (fig. 7). They lack lenses, show light absorbance under phase contrast microscopy, and can only be stained with acid dyes (Desch and Nutting 1978). Similar to Ixodida, Neocarus and Tetranychus mites show no microvillus-forming simple rhabdomeres (Evans 1992). Their absence suggests similarity to the ciliary basic (early) photosensor known only from the tadpole larvae of ascidians (Lamb 2013). Demodex “eyes” contain a handful of ciliary photoreceptors protruding from a large cell containing pigment (fig. 7).

Regarding photopigments, no specific rhodopsin orthologs (*Opn4* and *RRH*) were found in the Demodex, *D. farinae* or *S. scabiei* genomes, but these pigments were identified in the remaining 12 species surveyed. KEGG analysis revealed that all other genes in the Demodex phototransduction pathway are present. These three Acariformes lacking *Opn4* and *RRH* orthologs showed evidence of the presence of numerous other G protein-coupled receptors in their genomes, suggesting the existence of divergent opsin-like genes or pathways, differing from the rhabdomeric insect photoreceptor Gq but consistent with the Gt sequences or transduction pathways of vertebrates and those of other bilaterians (Lamb 2013). A similar opsin-like protein present in Demodex initiates the transduction cascade necessary in the detection of light.

While humans sleep, Demodex actively seeks mates and reproduces. Although these mites possess an almost complete circadian rhythm pathway, TIM (*Timeless*) is absent. Therefore, degradation by CRY, which drives daily resetting by light (Blum et al. 2018), is prevented, and Demodex sleeps during daylight hours. Coincidentally, the Demodex genome lacks arylalkylamine *N-acetyltransferase* (AANAT), a rate-limiting enzyme in melatonin synthesis; however, Demodex might utilize host melatonin, as observed in other parasites (Sack 2009). Interestingly, among the three major receptors of melatonin in animals, MT1, MT2, and MT3 (following mammalian nomenclature), Demodex presents a protein homologous to MT1 and seems to have lost MT2 but still synthesizes MT3; most of the other Acari in which the presence of melatonin receptors was examined showed clear hits against MT1. Melatonin is produced by most human tissues and is present at very high levels at dawn, allowing Demodex to detect it easily. Melatonin induces mobility and reproduction in invertebrates. The utilization of host melatonin by Demodex explains its nocturnal activity pattern and its total synchronization with the human host.

In accord with its nocturnal habits, Demodex lacks genes for UV protection, such as those required for the degradation of histidine (supplementary table SI 18, Supplementary Material online). Histidine ammonia-lyase, encoded by the *hutH* gene, converts histidine into urocanate, which acts as a natural sunscreen in some animals because it absorbs ultraviolet light (Barresi et al. 2011). Degradation to histamine is possible in *T. urticae* and *G. occidentalis* but not in Demodex. We found that the other portion of the histidine metabolism pathway involved in histidine degradation (linking l-histidine to l-glutamate) was present in all arachnid species surveyed as well as other Acariformes (Dong et al. 2018) but not in Demodex, and it was only partially present in *T. urticae* (supplementary table SI 18, Supplementary Material online). This section of the pathway includes the genes *hutH*, *hutU*, and *hutI*, whose products convert l-histidine to N-formimino-l-glutamate, and *ftcD* (glutamate formiminotransferase), whose product converts l-glutamate to N-formimino-l-glutamate.

### Is Demodex on an Evolutionary Dead-End Track?

The loss of DNA repair genes is likely to play a key role in genome degradation (Moya et al. 2008). Twenty-seven (27) genes that have been lost in the Demodex genome, ranging from DNA methyltransferase and mismatch repair genes to genes encoding exonucleases (e.g., *EXD2*), were annotated with the “DNA metabolic process” term.

Demodex is under relaxed selection. It has been shown that relaxed selection leads to an increased mutational load, which limits lifespan (Cui et al. 2019). If this phenomenon limits the lifespan of individuals, might it also eventually limit the lifespan of the species? In addition to being under relaxed selection, Demodex has lost many DNA repair genes. In a lineage of Hanseniaspora yeast, the loss of some DNA repair genes was shown to lead to a burst of accelerated evolution (Steenwyk et al. 2019). The loss of canonical nonhomologous end-joining (cNHEJ) genes to repair double-strand DNA breaks led to the evolution of the alternative end-joining pathway for repairing double-strand breaks in the urochordate *Oikopleura dioica* (Deng et al. 2018; Ferrier and Sogabe 2018). The loss of some DNA repair genes in free-living animals seems to be survivable, as observed in free-living bacteria, but most intracellular symbiotic bacteria that suffer increasing genome degradation likely go extinct due to being replaced by other bacteria or fungi (Latorre and Manzano-Marín 2016; Boscaro et al. 2018; Chong and Moran 2018; Matsuura et al. 2018; Mao and Bennett 2020). To escape this fate, an endosymbiotic bacterium can split into up to 10 different lineages, which then support each other (Campbell et al. 2015). Failing species can also be kept alive through the acquisition of complementary cosymbionts by the host. These compensation mechanisms are not available to animals. A close host association as a symbiont rather than as a pathogen has been shown to be a pathway to inevitable extinction (Santos-Garcia et al. 2017; Husnik and Keeling 2019; McCutcheon et al. 2019). A model of a genomic path to extinction has been established in bacteria (Bohlin et al. 2020), but this is a new concept in eukaryotes and in animals more specifically. The strong population bottleneck experienced by Demodex during vertical transmission to the next host generation and the associated drift will result in the accumulation of deleterious mutations characteristic of Muller’s ratchet (Allen et al. 2009; Melnikov et al. 2018). By the point at which Demodex enters a stable, nonpathological relationship with humans involving vertical transmission, extinction as a mathematical and statistical consequence might have already been determined (Bohlin et al. 2020).

## Materials and Methods

### Collection of *D. folliculorum* and DNA Sequencing

*Demodex folliculorum* were collected repeatedly from the forehead and nasal wings from a single person with the help of a black-head remover. Each collection contained around 40 mites. Voucher specimens are kept at the School of Biological Sciences, University of Reading, in the Acarology collection under the supervision of one of the senior authors. The collection of mites was reviewed by the Research Ethics Committee of The School of Biological Sciences, University of Reading, Approval Number: SBS 11 12 03 and SBS 12 13 16.

Under a high-magnification dissection microscope, specimens of *D. folliculorum* were individually identified at its various stages and morphotypes were separated, where necessary. The other human mite species, *D. brevis* was not identified from the same host, this was confirmed after analysing and identifying over 250 mites (corresponding to different collections/extractions from the same host). After identified as *D. folliculorum,* the mites were individually and manually cleaned using a tungsten tip of 1 µm, while submerged in physiological salt solution, after which were subjected to ultrasound bath for further cleaning in their watch glass, for up to 30 s. The extensive cleaning resulted in losses of up to 50% of the total mites extracted. DNA was extracted with the DNaeasy Blood and Tissue kit (Qiagen).

Number of *D. folliculorum*

**Table:** 

454 sequencing	∼120
HiSeq sequencing	∼50
MiSeq sequencing (mRNA)	∼80
Nanopore sequencing for maternal transmission analyses	∼300
Confocal microscopy	∼300

Six independent whole-genome amplification reactions using the GenomiPhi V2 DNA Amplification Kit (Cytiva) were performed and then mixed together for 454 sequencing and again for Illumina sequencing.

- 454 FLX+, three plates at FISABIO Center (Generalitat Valenciana)
- HiSeq 2000 (2× 100 bp) mate-pair sequencing (insert size 3 kbp) at Magrogen Inc. (Korea)

### Collection of *D. folliculorum* and RNA Sequencing

Samples were divided into males and females based on morphology and cleaned (as above) before RNA extraction. A third sample comprising all individuals including immature stages and eggs was sampled from within a single facial pore. RNA was extracted using a NucleoSpin RNA XS kit (Macherey-Nagel) for small tissue samples. cDNA synthesis was performed with the SMART-Seq v4 Ultra Low Input RNA Kit for Sequencing (Clontech Laboratories), with oligo(dT) priming. Library preparation was performed using the TruSeq DNA Nano kit (Illumina) with 350 bp inserts.

- MiSeq v2, 250 bp paired-end reads, Edinburgh Genomics (UK)

### Mitochondria

Samples for following the inheritance of Demodex were prepared, amplified, and sequenced using Alternate Protocol 1 of Zascavage et al. (2019), following the concept of Palopoli et al. (2015). Fragment 6, 1,442 bp, covering most of ATP 6, CoxIII, TRNG, and ND3, was amplified using primers adapted for *D. folliculorum*, F6Df 5′-CAG AAC TCC AAA CTT ACT TGT A-3′, R6Df 5′-TTT CAT TCT ATA ACT ATA ATT A-3′ (Ramos et al. 2009; Zascavage et al. 2019). Sequencing was performed on a MinION Mk1C instrument from Oxford Nanopore Technologies using the R9.4.1 flow cell. Sequences were aligned within Geneious R10 (Biomatters). Following the conclusions of Abadi et al., Generalized Time-Reversible plus Invariable sites plus Gamma distribution was used as an evolutionary model (Abadi et al. 2019, 2020). Phylogenetic reconstruction was performed with MrBayes (Ronquist et al. 2012).

### DNA and RNA Assembly

454 reads were pre-processed using PyroCleaner v1.3 (Jérôme et al. 2011). Sequences shorter than 100 bp or longer than 1 kb, with no base-calling quality score above 35, and with a “N” content of above 4% were discarded. For the assembly process, the filtered 454 reads were assembled using gsAssembler v2.6 (Roche) with default options. From this assembly, the mitochondrion genome sequence was detected using the *cox1* gene sequence and extracted from the assembly. Using bowtie2 (Langmead and Salzberg 2012), the reads were mapped back to the mitochondrion (reads mapping more than 80% of their sequence) and then separated from the read files. These mitochondrion-free reads were then assembled again using gsAssembler. This assembly resulted in 3,164 contigs with a total span of 52,889,837 bp. These contigs were then taxonomically assigned using PhymmBL v4.0 (Brady and Salzberg 2011) with a database of representative bacteria and archaea (NCBI), representatives from the Insecta class (*Acyrthosiphon pisum*, *Tribolium castaneum*, *Atta cephalotes*, *Drosophila melanogaster*) and Acari, *Tetranychus urticae*, as well as *Homo sapiens* and *Saccharomyces cerevisiae*. All sequences that were assigned to any other taxon other than Arthropoda with a score greater or equal to 0.75 were removed as contaminants.

Illumina HiSeq 2000 reads were pre-processed using a combination of FASTX-toolkit v0.0.14 of the Hannon lab at the Cold Spring Harbor Laboratory PRINSEQ-lite v0.20.4 (Schmieder and Edwards 2011). Sequences shorter than 75 bp, containing undefined nucleotides (“N”), or arising from sequencing artifacts (fastx_artifacts_filter) were discarded. 3,100 contigs along with the Illumina reads that were found to map to them (using bowtie2 with the –very-sensitive algorithm) were used as input for scaffolding (one round, options -k 50 -g 2 -a 0.7 -n 15) and gapFilling (five rounds, options -k 50 -g 2 -a 0.7 -n 50) using SSPACE v2.0 (Boetzer et al. 2011) and GapFiller v1.11 (Boetzer and Pirovano 2012), respectively. This processing resulted in 575 contigs ordered into 306 scaffolds. These 306 scaffolds were then corrected for base calls using the Illumina reads and Polisher v2.0.8 (LaButti et al. 2008) and coverage was assessed by mapping back both 454 and Illumina reads using Bowtie 2. Contigs whose coverage fell under the 1st quartile (145.4×) were dismissed if they were short (<1 kb) and lacked genes, or if they showed signs of belonging to bacterial contamination, having only 242 scaffolds remaining. Additionally, all genes that showed as best hit a bacterial gene from the maker output were checked for possible signs of contamination (no introns or present in contigs made up of exclusively bacterial genes), having one small contig being removed. Information from RNA sequencing allowed to reduce the number of scaffolds to 241.

Raw RNA-Seq reads were assessed for quality using FastQC v0.11.5 (Andrews 2010) and then adapter and quality trimmed with the program Trimmomatic v0.36 (Bolger et al. 2014) to produce trimmed paired reads and those unpaired by the trimming process. Trimming was performed when base quality scores went below a threshold of Q22 when averaged across 4 bp windows (SLIDINGWINDOW: 4:22), retaining trimmed reads more than 40 bp in length. Adapter trimming included removal of the TruSeq as well as SMART-Seq adapters, in palindromic mode. Due to the interaction of TruSeq and SMART-Seq preparation, it was expected that SMART adapter contamination would occur (including polyA/Ts). This includes both 5′ and 3′ adapters, as well as up to 5 bp of non-templated DNA to the 5′ end of the RNA template. To account for the latter contamination, a 5 bp crop was performed on both ends reads, resulting in an average read length of 240 bp. FastQC assessment of read quality and adapter contamination confirmed the efficacy of our trimming protocol.

Trimmed reads were then concatenated (forward and reverse separately), and used to perform de novo transcriptome assembly in Trinity v2.4.0 (Grabherr et al. 2011). First, 7,744,482 paired reads (15,488,964 total) were mapped to our high-quality genome assembly using HISAT2 v2.1.0 (Kim et al. 2015) with default parameters. Mapping resulted in a 92.4% overall alignment rate. These mapped reads were used to perform a genome-guided de novo Trinity assembly, which resulted in 21,426 Trinity “genes” (32,984 total transcripts). Paired reads and 7,391,766 reads unpaired during trimming were then used to perform a de novo Trinity assembly with default parameters. This produced 25,535 Trinity “genes” (41,548 total transcripts). Both assemblies were then used to inform an update in the nuclear genome feature annotations.

Feature annotation update was performed using PASA v2.2.0 (Haas et al. 2003). Briefly, both genome-guided and fully de novo assemblies were aligned to the genome assembly using BLAT v35 (Kent 2002) and GMAP v2017-01-14 (Wu and Watanabe 2005). A database of annotations produced by MAKER2 was created and updated in successive rounds from assembled transcripts. New annotation versions included 5′ and 3′ untranslated regions (UTRs), alternative isoforms of genes, merged or split genes (compared with the original annotation), and novel genes not discovered by *in silico* procedures. Novel genes were included only when a transcript was determined as being full length by TRANSDECODER (Haas et al. 2013), and annotation updates only included transcripts that mapped to the genome assembly. Nine rounds of PASA updates were performed before updating was deemed complete (i.e., updates did not change annotations). In total, 43% of genes showed some form of update, resulting in a set final set of 9,707 genes. This included 99 merged genes from the in silico predictions, 41 split genes, and 28 novel genes. In total, 7,575 5′ UTRs and 6,738 3′ UTRs were added to the final annotation.

Mutation patterns in the RNA-Seq data were examined using Rnaseqmut v0.6 (https://github.com/davidliwei/rnaseqmut) to quantify mutations from quality trimmed reads mapped to the genome assembly using HISAT2 v2.1.0. Mutations were called at positions with >10× coverage, allowing for no more than three mismatches per read.

### Annotation and Enrichment

Ab initio gene annotation was carried using MAKER2 (Holt and Yandell 2011).

RepeatModeler v1.0.11 and RepeatMasker v4.0 (Smit and Hubley 2008; Smit et al. 2013) were used to model and quantify repeats in the genome, including interspersed and short sequence repeats. Metrics of genome feature counts and lengths were generated from existing feature annotations for each genome and focussed only on coding sequence features as most draft genomes do not include features such as UTRs, potentially biasing metrics, particularly intron/exon lengths. Mean and median exon and intron counts and lengths were calculated for one isoform per gene, where multiple isoforms were annotated. Distance between coding gene annotations does not include distances between a coding sequence and the end of a scaffold/contig.

Annotation of *D. folliculorum* genes and orthogroups across all 15 species was carried out using Blast2GO v4.1.9 (Conesa et al. 2005; Götz et al. 2008) and blastKOALA (Kanehisa et al. 2016). Blast2GO was run with an *e*-value cut-off of 1e-5, and remaining parameters as default, to obtain Gene Ontology (GO) terms for Biological Processes (BP), Cellular Component (CC), and Molecular Function (MF). BlastKOALA was run using species specific NCBI taxonomy identifiers. Both programs were used to obtain GO terms and KEGG Orthology (KO) groups for *D. folliculorum*, *T. urticae*, and *Galendromus* (*Metaseiulus*) *occidentalis*. GO terms for *D. melanogaster* genes were obtained from Ensembl Metazoa using BioMart (Kinsella et al. 2011). Annotations for each species were then used to form consensus annotations for ortholog groups from Orthofinder by merging annotation terms from each species, resulting in a set of consensus GO terms and KO functions. Because KEGG orthology groups map to orthologs in the KEGG database (Kanehisa and Goto 2000; Kanehisa 2019; Kanehisa et al. 2019), Orthofinder results could be verified, for the subset of orthogroups to which KOs were assigned. In total, 5,255 orthogroups had KO annotations, with 3,991 coming from at least two species (i.e., were consensus). Of these, 3,671 showed agreement in KO assignment, demonstrating a 92% agreement rate between Orthofinder and blastKOALA.

Functional enrichment of slim GO terms assigned to gene family test sets were carried out in the R package “GOfuncR” (Prüfer et al. 2007; Grote 2018). Slim GO terms are a subset of the full gene ontologies and provide a broad overview of gene functions. GO slim terms for each orthogroup were obtained by converting specific terms assigned to each orthogroup into Biological Process GO slims using the R package “GSEABase” (Morgan et al. 2018), using the “Generic GO subset” and OBO_edit (Day-Richter et al. 2007) from the Gene Ontology Consortium (Ashburner et al. 2000; Gene Ontology Consortium 2019). GO slims were used to perform functional enrichment tests using defined test and background sets. The hypergeometric test from GOfuncR was used to determine overrepresented terms and is equivalent to a one-sided Fisher’s exact test. To account for performing multiple tests, and thus encountering significant degrees of false discoveries, both the default GOfuncR FWER (family-wise error rate, with 1,000 permutations), and *q*-values (R package “qvalue”) (Storey and Tibshirani 2003; Bass et al. 2018) were calculated for each test. Significance was considered based on either the *q*-value or FWER, at a 10% threshold of acceptance. For most tests, all orthogroups with assigned GO terms were used as the background set. For relaxed selection test enrichment, the full set of input groups to the selection test (467 orthogroups) was used as the background because this set is already a subset of all orthogroups. Full GO terms in orthogroups assigned significantly enriched GO slims were used to make functional figures in REVIGO (Supek et al. 2011). REVIGO takes long lists of GO terms and shrinks them down to their least dispensable terms. Figures were made in R using the package “ggplot2” (Wickham 2016), and included the frequency of terms within each list, and labeled with prominent terms.

BUSCO was run for both eukaryote and arthropod sets of single copy orthologs, on released curated protein sets for each genome, filtered for longest isoform per gene where isoforms were annotated (Waterhouse et al. 2018). BUSCO v2.0 was used to assess the completeness of gene annotations by comparing gene annotations to a set of universal single copy genes found in 1) eukaryotes and 2) arthropods. BUSCO was run in protein mode using the longest isoform of a gene for each species, for those species where alternative transcripts have been annotated.

ENCprime was used to calculate the number of effective codons across coding sequence sets (Nc) as well as Nc′, a measure of codon use corrected by the background mutation pattern (Novembre 2002). Corrections were performed using the nucleotide frequency of the third codon position for each respective gene under study. Nc and Nc′ vary from 0 to 61 but are designed to scale broadly to mirror the number of codons used.

### Gene Family Analyses

To examine gene family size changes during the evolution of *D. folliculorum*, orthology of genes across 15 species of arachnids and outgroups was determined using Orthofinder (Emms and Kelly 2015), and used to model expansions and contractions over a species tree inferred using IQ-TREE (Nguyen et al. 2015; Chernomor et al. 2016; Hoang and Chernomor 2017; Kalyaanamoorthy et al. 2017; Wang et al. 2018; Crotty et al. 2020). Constraint and unconstrained trees were used to examine gene family evolution with the program CAFE (Computational Analysis of Gene Family Evolution) v4.0 (De Bie et al. 2006).

Orthofinder v2.2.3 was used to determine orthologous genes across a test set of 15 species, chosen for their high-quality gene annotations. The set of 15 species included representatives from the Acariformes (4), Parasitiformes (4), spiders (2), and scorpions (1), as well as outgroup species (4). Although additional mite and tick genomes exist, most do not have annotated gene models, or these gene models are not very complete.

The expansion and contraction of gene families (birth and death of genes) was modeled using CAFE. CAFE uses a rooted and ultrametric tree to model the evolution of gene counts within gene families across a phylogeny. Two trees were analyzed in CAFE: 1) constrained topology with monophyletic Acariformes and Parasitiformes, and 2) unconstrained tree topology. Trees from IQTREE were rooted and converted to time trees using the R package APE v5 (Paradis and Schliep 2019). Rooting was performed at the Chelicerata/outgroup branch and calibrated to time using the “chronopl” function. Divergence can be calibrated based on ranges of divergence times, which were obtained from a mixture of fossil record dates and previously inferred splits from Jeyaprakash and Hoy (2009) and Pisani et al. (2004). For topology 1) these were as follows: Chelicerata/outgroup divergence min = 679 Mya, max = 771 Mya; Insecta/Crustacea divergence min = 608 Mya, max = 724 Mya; *Limulus*/Arachnida divergence min = 422 Mya, max = 528 Mya. For topology 2) these were as follows: Chelicerata/outgroup divergence min = 679 Mya, max = 771 Mya; Insecta/Crustacea divergence min = 608 Mya, max = 724 Mya. Recent phylogenomic work using multiple crustacean and chelicerate species approximated their divergence around 630 Mya, slightly shallower than our calibration range (Schwentner et al. 2017). Although calibrating deep splits can be challenging, we found our divergence time estimates to mirror Pisani et al. (2004), and internal nodes not specifically calibrated were consistent with previously published results (e.g., *Drosophila*/*Tribolium* split of ∼330 Mya).

Orthogroup data from Orthofinder were used to count the number of genes in each group by species which was then filtered for input into CAFE. Filtering included removing the largest gene clusters, orthogroups were removed if any one species had >100 genes. This set of “large” orthogroups were analyzed separately later using parameters estimated by the filtered dataset. CAFE requires at least two genes within lineages in order to perform ancestral state reconstruction at the LCA of a clade. Thus, filtering was performed to retain as many groups as possible but provide informative data on the clades of interest (arachnids). Orthogroups were retained that had at least two species represented within mites and ticks, and at least two species within the spiders/scorpion/*Limulus* grouping, ensuring the retention of groups containing mites and ticks, but also ensuring retention of information on the LCA of the arachnids. In total, Orthofinder produced 14,072 orthogroups. After filtering, 7,058 were retained for analysis, and 32 were considered “large” orthogroups containing >100 genes in any one species. These groups contained 7,268 *Demodex* genes, or 74.5% of the predicted proteome.

CAFE analysis can be carried out on a number of different models of gene family evolution, and modeling can also include an error rate caused by sources such as genome assembly or annotation errors. The simplest model is one where CAFE estimates a global lambda, a single and combined probability that a gene will be lost or gained. The “lambdamu” command allows for separate birth and death rates to be estimated across the tree. “Multi” models of either the combined lambda or separate lambdamu estimates allow these parameters to be estimated separately for user defined subsections of the input phylogenetic tree. We examined several different models using error rates estimated from the data. Error rate estimation was performed using the supplied Python script “caferror.py” with default parameters. This script runs CAFE multiple times using different error rates and optimizes the error rate each time based on the likelihood score, producing an error rate model. CAFE runs for this error rate estimation were performed using the filtered data for each alternative tree topology and CAFE was subsequently run using the resulting error models. The optimized global error rate was 1.22 × 10^−5^, for both tree topologies.

CAFE was run separately on each tree topology, running four different models: 1) single lambda run, 2) single lambdamu, 3) multiple lambdamu with separate probabilities for the Acariforme branches, and 4) multiple lambdamu with separate probabilities for both the Acariformes and Parasitiforme branches. Five runs of each model were performed to check convergence in parameter values and log-likelihoods. Within each tree topology, likelihoods were ranked for comparison. Likelihood improvement between single lambda estimates and multiple lambda models was compared using the CAFE “lhtest” method where CAFE uses the input data to simulate a null distribution of single and multiple lambda likelihood ratio differences between models (“genfamily” function, 100 simulations) which can be used to test if the estimated likelihood ratio between these models differ significantly from this null. The difference in single lambda was test against multiple lambdas where Acariformes, Parasitiformes, and the remaining branches were allowed separate probabilities.

### Species Tree Inference

Species tree inference was carried out using protein alignments generated by Orthofinder. In total, Orthofinder discovered 1,316 orthogroups containing at least 87.5% of genes from all species for species tree reconstruction. This dataset was concatenated but unpartitioned. A high stringency partitioned dataset was also used to explore the species tree, consisting of only single copy orthogroups containing sequences from all 15 species. This smaller set of gene families contained 356 orthogroups. The known issues surrounding the reconstruction of arachnid phylogenomic relationships mean that it is prudent to filter rapidly evolving genes or alignment sites, in order to account for saturation effects in the Maximum Likelihood (ML) analysis. Sharma et al. (2014) examined both the 200 and 500 of the slowest evolving genes in their dataset, in order to reduce saturation and Long Branch Attraction (LBA) caused by heterotachy (rate variation across tree branches). Tree distance can become saturated at higher pairwise divergence but removing sites with higher evolutionary rates can reduce this effect. Following Sharma et al. (2014), we removed the most rapidly evolving sites or genes from our analyses.

The “unpartitioned” data were filtered to remove rapidly evolving sites based on rate categories determined by IQTREE (Chernomor et al. 2016; Kalyaanamoorthy et al. 2017; Wang et al. 2018; Crotty et al. 2020). Sites categorized as being in the two highest rate categories under the LG + H4 model were removed resulting in an alignment containing 229,939 sites (76,844 parsimony informative, 55,029 singletons, and 98,066 invariant sites). Protein sequences in the 356 gene “partitioned” data set were aligned using MAFFT v7.397 (Katoh and Standley 2013) and ML pairwise distance between sequences were obtained using IQTREE. Tree searches were performed initially using ModelFinder (Kalyaanamoorthy et al. 2017) searching for the top evolutionary model based on BIC, which chose some form of the base model LG as the top model for ∼80% of alignments. After removing the most rapidly evolving genes, the remaining set of gene families consisted of the 170 gene alignments for phylogenomic analysis. Each dataset (unpartitioned and partitioned) was processed by trimAl (Capella-Gutiérrez et al. 2009), using the “-automated1” preset, to remove extensive gaps in the data. Alignment concatenation and partition file generation was performed by TriFusion (Fischer et al. 2011).

Unpartitioned data were used to run mixture models in IQTREE (CAT models), which can be used to account for rate heterogeneity across sites (Wang et al. 2018). Further, the IQTREE heterotachy (“H”) models were used in order to attempt to account for branch-specific rates of evolution (Crotty et al. 2020). Model searches for the unpartitioned data were performed using the “-madd” parameter to include CAT models (C10, C30, C50, C60), and heterotachy models (H4, H8). Combinations of CAT and heterotachy models were also examined. The CAT model with 60 mixture rates consistently came out as the top model, alone or in combination with heterotachy models. Tree searches were performed with C60 and heterotachy models of different rates and included 1,000 rapid bootstraps (“-bb” flag). The same tree topology was found for each search, regardless of the model used, and was always extremely well supported, with most nodes consistently supported by 100% of bootstrap trees. Increased mixture rates and heterotachy models had only a minimal effect of reducing branch lengths, and effectively reduced the arachnid diversification to a three-way polytomy between spiders/scorpion branches, Acariformes, and Parasitiformes.

Long branch attraction can occur when the high evolutionary rate of some lineages in a tree pulls those lineages together and, generally, towards the root. Phylogenetic reconstruction of some Acariformes and Parasitiformes trees has resulted in such long branch lengths and suspect topologies, presumably driven by heterotachy (Jeyaprakash and Hoy 2009; Sharma et al. 2014). Additionally, the diversification of the arachnids appears to be ancient and rapid so that saturation of sites is likely to make recovery of the true topology and timing of nodes difficult to recover. The top evolutionary models containing high rates of change were consistently chosen by ModelFinder for both datasets, suggesting extensive saturation. The presence of lineage-specific and extreme rates of evolution within a tree containing only 15 species mean that nodes of the recovered topology are likely to be suffer from LBA, and be ordered by the branch lengths of the recovered groups. This appears to be the case here where the longest branches are seen in Acariformes, then Parasitiformes, and lastly spiders/scorpions, with the order of splits following branch length from outgroups towards the tips. This topology and LBA issue was also seen by Sharma et al. (2014) using similar data. Sharma et al. (2014) concluded that the topologies they recovered likely suffered from LBA.

To test the likelihood of alternative tree topologies in our data, the partitioned data of 170 slowest evolving genes was assessed using both unconstrained and constrained trees in IQTREE. This partitioned dataset included 75,300 sites: 38,713 parsimony informative, 11,918 singletons, and 24,699 invariant sites. Four alternative topologies were tested, with two basic assumptions for each constrained tree: 1) the most likely recovered topology might be the result of long branch attraction, as proposed above and in Sharma et al. (2014) and 2) that *Limulus polyphemus* is outgroup to arachnids; for a comprehensive reference list, please see Extended Data supplementary table S8, Supplementary Material online. Constraint trees were 1) “mite/tick monophylly”, groups: outgroups incl. *L. polyphemus*, Acariformes with Parasitiformes grouped, and scorpions/spiders; 2) “spiders/scorpions and Parasitiforme monophylly”, groups: outgroups including *L. polyphemus*, spiders/scorpions with Parasitiformes, and Acariformes; 3) “spiders/scorpions and Acariforme monophylly”, groups: outgroups incl. *L. polyphemus*, spiders/scorpions with Acariformes, and Parasitiformes; and 4) “*L. polypheus* as outgroup” constraint only on *L. Polyphemus* as outgroup to arachnids, topology within arachnids unconstrained. Each tree was run using the “-sp” option and with the top model from ModelFinder, searching each partition for protein models that included FreeRates models, and merging partitions based on the top 1% of models (the latter because exhaustive searches are too computationally intensive). The final trees for each model were then concatenated and the likelihood of each compared in IQTREE with *P*-values for differences generated by the “AU” method (“-au”) and 1,000 resamplings.

The next best topology compared with an unconstrained tree, based on likelihood, was one where Acariformes and Parasitiformes were grouped and spiders/scorpions were basal to arachnids. This topology was recovered in both the tree where Acariformes and Parasitiformes were constrained together [tree (1)], and where the topology was unconstrained except for the specification that *L. polyphemus* is outgroup to arachnids [tree (4)]). Gene family evolution analyses focussed on this constraint tree but was conducted using both this and the unconstrained tree for comparison.

The current models for the evolution of Acari lineages and the position of Xiphosura (horseshoe crabs) and the support for these models has been reviewed in supplementary table S8, Supplementary Material online of the Extended Data. More and more, horseshoe crabs are seen as derived from terrestrial ancestors that have returned to the sea, no longer occupying a basal position the chelicerates (Ballesteros and Sharma 2019; Noah et al. 2020).

### Natural Selection

Genome reduction in small genome mites may have involved the relaxation of natural selection, leading to gene loss over time. To test the hypothesis that a general relaxation of selection played a role in the reduction of the number of genes in the genome, we used the program RELAX (Wertheim et al. 2015). RELAX tests a user defined set of “Test” and “Reference” branches for changes in the intensity of natural selection (either intensification or relaxation of selection) based on differences in the ratio of nonsynonymous to synonymous mutations (dN/dS or Ω). To examine the role of relaxation in gene loss in Acariforme mites, selection was tested for all Acariforme branches with signals of overall gene loss and compared with the spider and scorpion branches showing a consistent pattern of gene expansion, allowing the comparison of the mechanisms by which gene sets within arachnid species may expand or contract.

The POTION pipeline v1.1.2 (Hongo et al. 2015) was used to align and filter orthologs across species, using the Orthofinder ortholog groups to extract single copy genes for testing. Input data were checked to ensure correct translation frame, then cleaned, aligned using PRANK v170427 (Löytynoja 2014), and filtered by POTION, checking that each sequence was a multiple of three and did not contain ambiguity characters. Additionally, sequences were removed if they fell below a minimum length of 150 bp, and if pairwise amino acid distance for any one sequence fell below 15%. Paralogs were removed from an orthogroup, if they existed. To obtain robust measures of dN/dS, orthogroups were removed from analysis if they contained less than 11 species, or were missing any arachnid species, except for a single Parasitiforme (to maximize the data set but retain balance in the number of branches across arachnids). An edited species tree for each orthogroup with corresponding absence of species was used in order to test the same corresponding branches in each group. These branches were: five Acariformes branches demonstrating gene family contraction, and five spider/scorpion branches demonstrating gene family expansions. RELAX outputs include the “*K*” parameter, which measures degree of difference in selection strength between the Test and Reference branches (*K* < 1 indicates relaxation, *K* > 1 indicates intensification of selection). RELAX also estimates the Test and Reference dN/dS, and performs a likelihood ratio test between a null model with *K* equal to 1 (no change in omega between branches) and the alternative where *K* is not equal to 1. *P*-values from the likelihood ratio test were adjusted to account for the effects of false discoveries when performing multiple tests using the R package “SGoF” v2.3 (Carvajal-Rodríguez et al. 2009; Castro-Conde and de Uña-Álvarez 2015), with a significance threshold of adjusted *P* less than 10%.

### *Hox* Genes

Species used in Hox gene analyses: *D. folliculorum* (Acariformes) this work; *Teranychus urticae* (Acariformes) (Grbić et al. 2011), the orientation of *Dfd* and *ftz* in *Tribolium castaneum* should have the opposite orientation, the orientation of the *Hox* cluster of *Drosophila melanogaster* should be inversed in relation to *Tribolium castaneum* (Zhang et al. 2019); *Sarcoptes scabiei* (Acariformes) (Rider et al. 2015; Mofiz et al. 2016); *Galendromus* (*Metaseiulus*) *occidentalis* (Parasitiformes) (Hoy et al. 2016); *Ixodes scapularis* (Parasitiformes) (Gulia-Nuss et al. 2015); *Folsomia candida* (Entognatha) (Faddeeva-Vakhrusheva et al. 2017); *Drosophila melanogaster* (Insecta) (Negre and Ruiz 2007); *Caenorhabditis elegans* (Nematoda) (Aboobaker and Blaxter 2003); *Paracyclopina nana* (Crustacea) (Kim, Kim, et al. 2016); *Lingula anatina* (Brachiopoda) (Luo et al. 2015); *Strongylocentrotus purpuratus* (Echinodermata) (Cameron et al. 2006).

### Confocal Microscopy

Demodex were collected repeatedly and prepared as for DNA and RNA sequencing. The confocal laser scanning microscope LSM 510, Carl Zeiss, at the School of Natural Sciences, Bangor University, was used.

To estimate the number of cells for immature stages of *Drosophila melanogaster*, nuclei were stained with SYTOX green and propidium iodide. Half of the body was divided into 6 × 4 sections and counted. For *D. folliculorum*, the body was divided into three parts: gnathosoma and podosoma, ganglia and upper opistosoma, and into lower opistosoma.

For estimating nuclear ploidy levels in *D. folliculorum* females, each of the 1,400 nuclei were quantified. Ploidy levels were assigned based on the result of the fluorescent intensity obtained from the estimation of the genome size. The assumption is that the lowest fluorescent intensity is 2C or diploid in all female specimens. Every value obtained from the fluorescence intensity of a nucleus that is greater than the 2C value is assigned to the next higher ploidy level. Measurements were made of all nuclei. Results were then read from the histogram section of the CLSM. The procedure ZAMITI (Z-Stack, Area of Histogram, Mean intensity, Intensity of cell and Total intensity) was adopted (Shimizu et al. 2008).

Microphotographs of whole specimens stained with 4′,6-diamidino-2-phenylindol (DAPI) were used to localize tissue/organs and nuclei. They were stained after fixing in a light Carnoy solution (v/v, 60% ethanol/40% glacial acetic acid) which helps preserving structures and provides a background for localization, visible in the same UV channel (∼300 nm) or combining with another channel at 560 nm (wavelengths for excitation).

## Supplementary Material

msac125_Supplementary_Data

## Data Availability

Raw sequencing reads and genome assembly files have been uploaded to GenBank project accession: PRJEB13411, DMDXMAP.
