# Supplementary material for: Human Follicular Mites: Ectoparasites Becoming Symbionts
Source: Mol Biol Evol. 2022 Jun 21;39(6):msac125. doi: 10.1093/molbev/msac125 (PMC9218549; doi:10.1093/molbev/msac125)
Supplement: msac125_Supplementary_Data [file msac125_supplementary_data.pdf]

Supplementary Information

Human follicular mites: Ectoparasites become symbionts

Gilbert Smith, Alejandro Manzano Marín, Mariana Reyes-Prieto, Cátia Sofia Ribeiro Antunes, Obed Nanjul Goselle, Victoria Ashworth, Abdulhalem Abdulsamad A. Jan, Andrés Moya, Amparo Latorre, M. Alejandra Perotti, Henk R Braig

Contents

1 Supplementary Figures and Table 3

Fig. S1 Demodex is maternally inherited 3
Fig. S2 Functions of rapidly evolving gene families of Demodex and Acariformes 4
Fig. S3 Mitochondrial genome has polycistrons 5
Fig. S4 Distribution of the RELAX parameter K across significant selection tests 6
Fig. S5 Host association determines extend of AT-bias of invertebrate and vertebrate animals 7
Tab. S1 Intergenic distances and introns 8
Fig. S6 Endopolyploidy in Demodex 9

2 Supplementary Items (SI) 10

Fig. SI 1 Benchmarking Universal Single Copy Orthologs 10
Fig. SI 2 Length distribution of proteins in parasitic/endosymbiotic Demodex and free-living/plant-parasitic Tetranychus 11
Tab. SI 1 Probably pseudogenes of Demodex arranged according to Demodex AA length 12
Tab. SI 2 Orthogroups of Demodex rapidly expanding 14
Tab. SI 3 Minimal genome sizes and number of coding genes per taxonomic clade in the Pan-Arthropoda 15
Tab. SI 4 Species used in genome analysis 18
Tab. SI 5 Repeat content of mite genomes 20
Tab. SI 6 Intergenic distances and introns 21
Fig. SI 3 Host association determines extend of AT-bias of Acariformes 23
Fig. SI 4 Genome-wide codon usage bias in Acariformes 25
Fig. SI 5 Mutation spectrum for Demodex demonstrates an AT-mutational bias 27
Tab. SI 7 Orthogroups (OGs) of arthropod species 28
Tab. SI 8 Computational analysis of gene family evolution model parameters 30
Fig. SI 6 Computational Analysis of Gene Family Evolution lhtest histogram 32
Fig. SI 7 Alternative IQtree topologies 33
Tab. SI 9 Gene families showing rapid contraction in Demodex 34
Tab. SI 10 Gene families showing rapid contraction in the Acariformes 38
Tab. SI 11 Gene families showing rapid expansion in the Acariformes 42

Tab. SI 12	Gene family loss in Demodex	46
Tab. SI 13	Gene family loss in the Acariformes	50
Tab. SI 14	RELAX test significant orthogroups	54
Tab. SI 15	Relaxed selection enrichment	58
Tab. SI 16	Intensified selection enrichment	62
Tab. SI 17	Counting of nuclei in *Drosophila melanogaster*	66
Tab. SI 18	Histidine pathway genes	67
	References	68

1 Supplementary Figures and Table

Demodex is maternally inherited

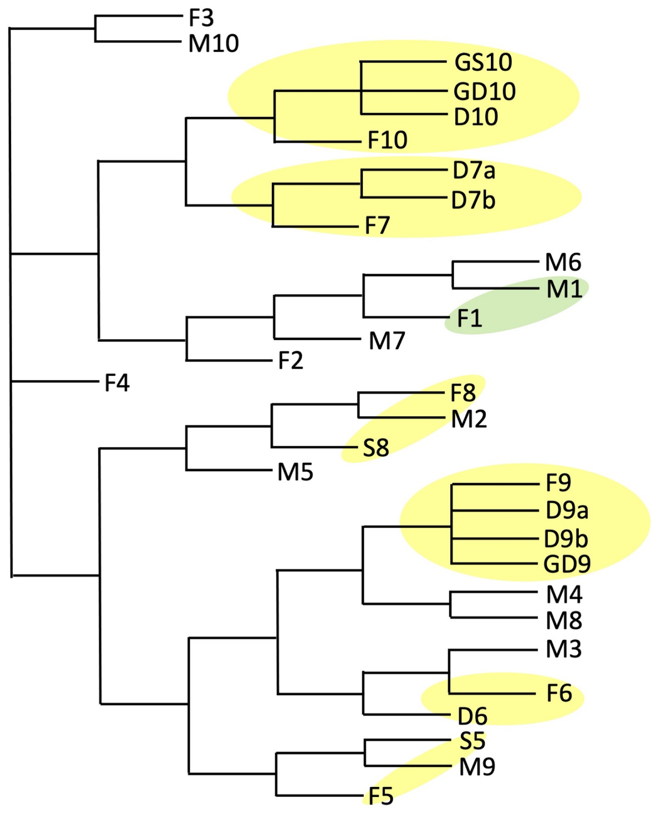

Figure S1. Demodex is predominantly maternally inherited. Cladogram of partial mtDNA of *D. folliculorum.* Six maternal relationships highlighted in yellow. Relationships: F: female, M: male, D: daughter, S: son, GD: granddaughter, GS: grandson; female and male couples: F1, M1: female 1 with male 1, GD9a is the daughter of D9a who is the daughter of F9. For horizontal transmission, it is expected that couples carry similar lineages of mites. Female and male couples do not carry similar mite lineages with the exception of F1 (green background). For vertical transmission, it is expected that offspring clusters with maternal lineage (yellow background0. Without exception, this is the case for all six families analysed. F10 (mother) clusters daughter D10 and granddaughter GD10 and grandson GS10. F7 (mother) clusters with her two daughters D7a and D7b. F8 (mother) clusters with her son S8 as is the case for F5 (mother) and son S5; mother F6 (mother) and her daughter D6; and F9 (mother) and her two daughters D9a and D9b and her granddaughter D9.

Functions of rapidly evolving gene families in Demodex and Acariformes

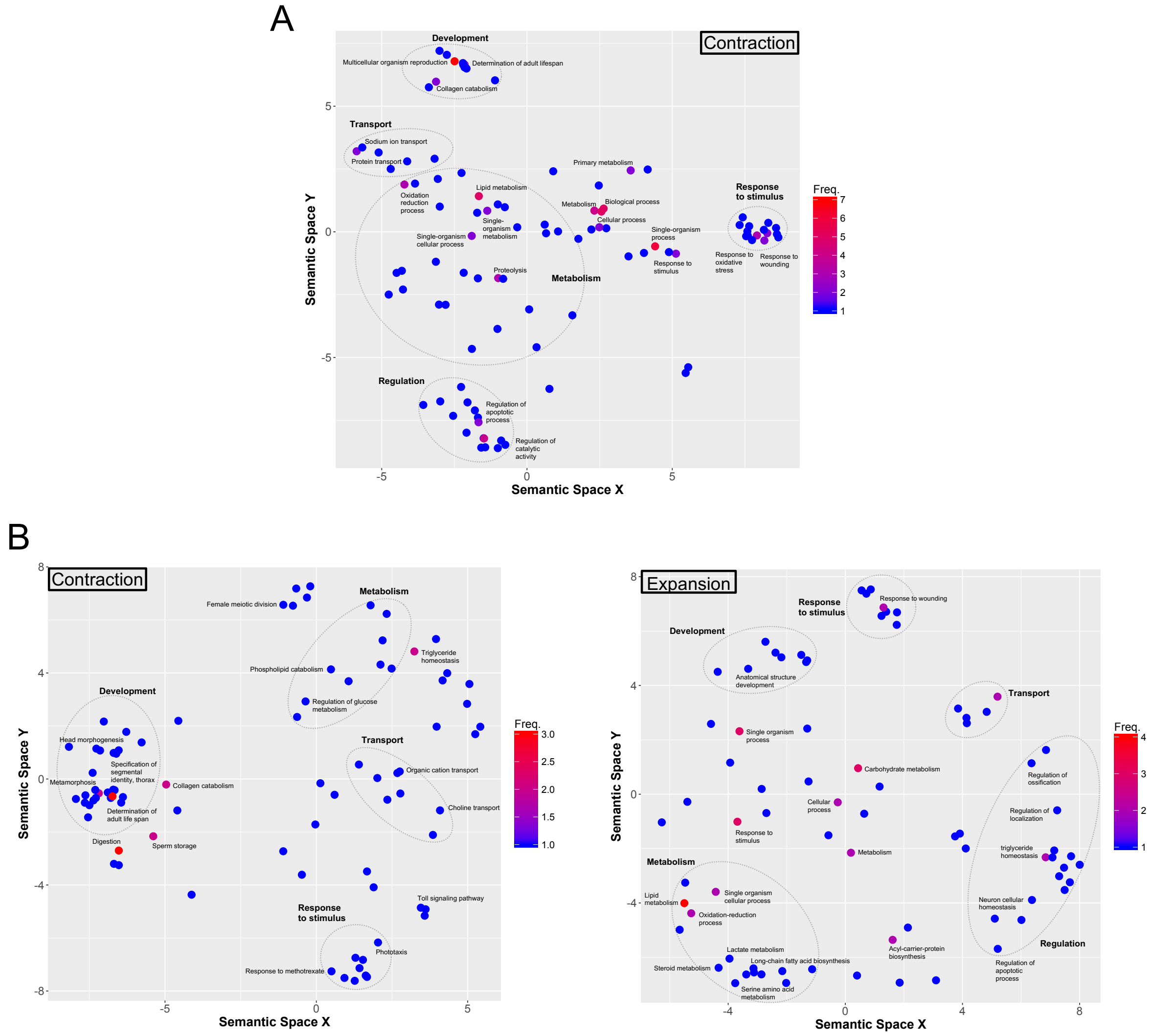

Figure S2. Functions of rapidly evolving gene families. **A** Contracting gene families in Demodex. **B** Contracting gene families Acariformes. C Expanding gene families in Acariformes. Eight cryptic orthogroups expanding in Demodex are listed Tab. SI 2.

Mitochondrial genome has polycistrons

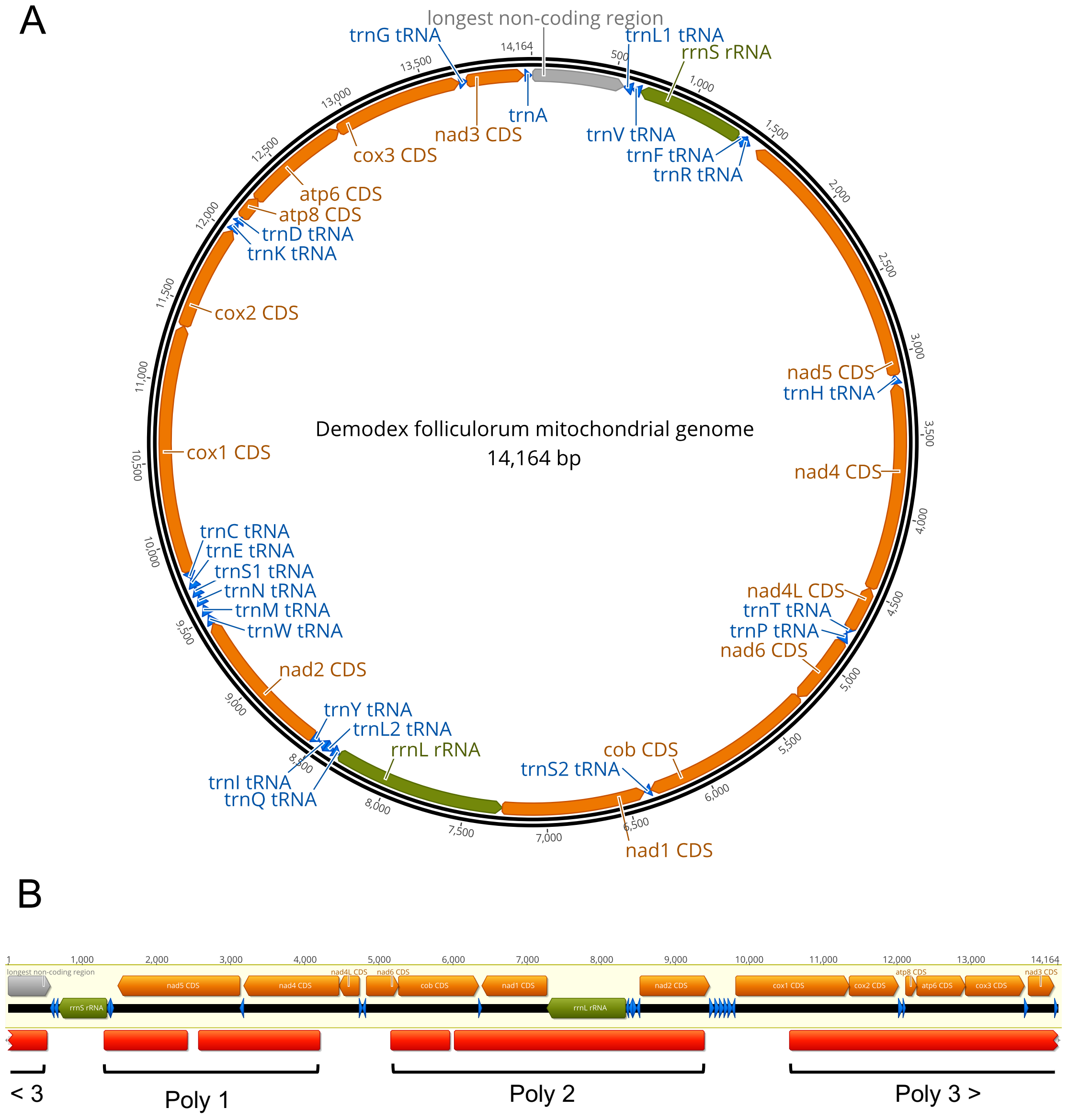

Figure S3. Polycistrons in the mitochondrial genome of the human follicle mite, *D. folliculorum*. **A**: Mitochondrial genome annotated with protein coding (orange) and non-coding (green) genes. **B**: Linear view of the mitochondrial genome with transcript-based evidence for polycistronic expression (red: labelled "Poly" for each polycistronic unit). Transcripts were assembled from RNA-sequencing data and mapped to the mitochondrial reference.

Distribution of the RELAX parameter K across significant selection tests

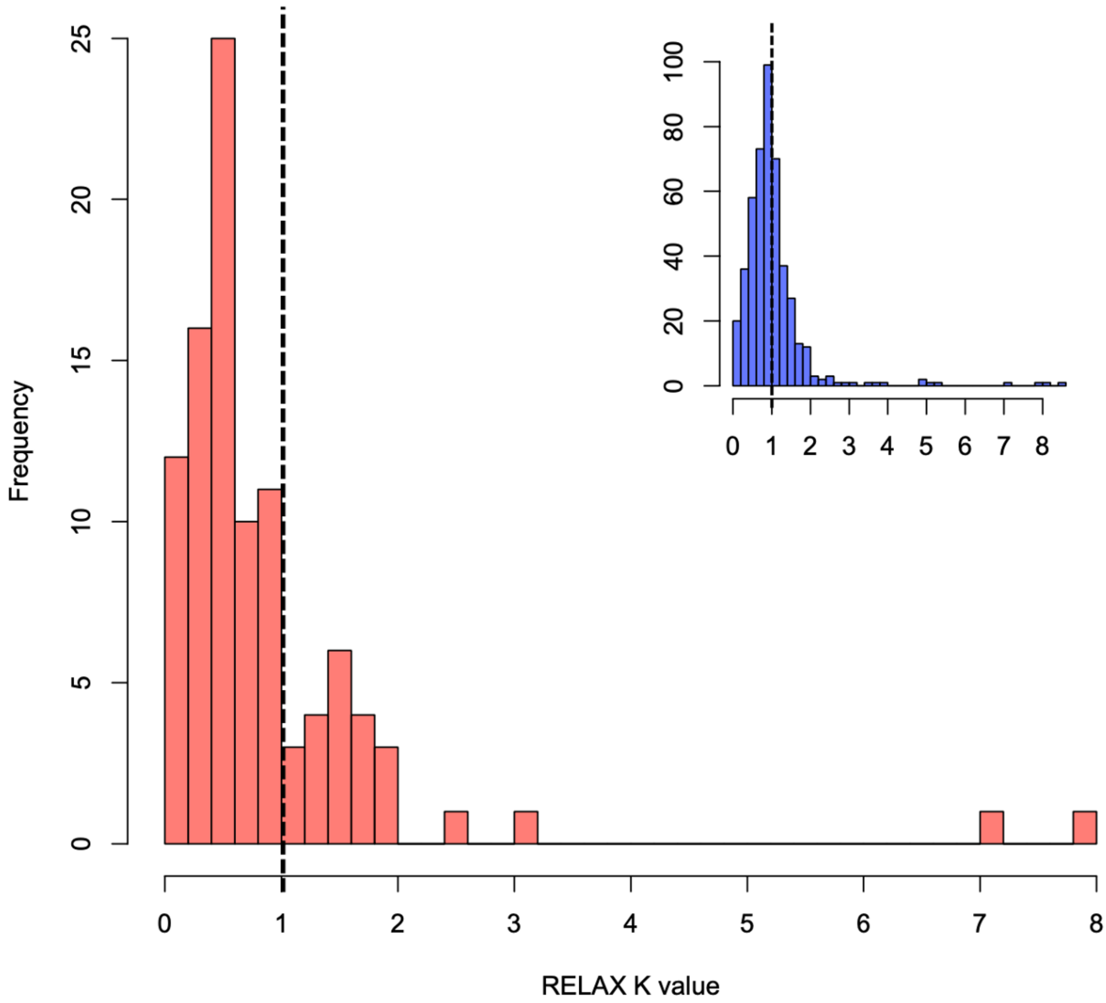

Figure S4. Relaxed selection is more common than intensified (purifying) selection in Acariformes compared to spiders and scorpions. Distribution of the RELAX parameter K across significant selection tests (adjusted $p < 0.1$; red bars), and all tests (blue bars, inset). The density plot of omega values (non-synonymous/synonymous substitutions: dN/dS) for genes showing relaxation of selection (K < 1; main) and intensified selection (K > 1; inset) in Acariformes and Functions of genes under relaxed selection in Acariformes are depicted in Fig. 2 of the main paper.

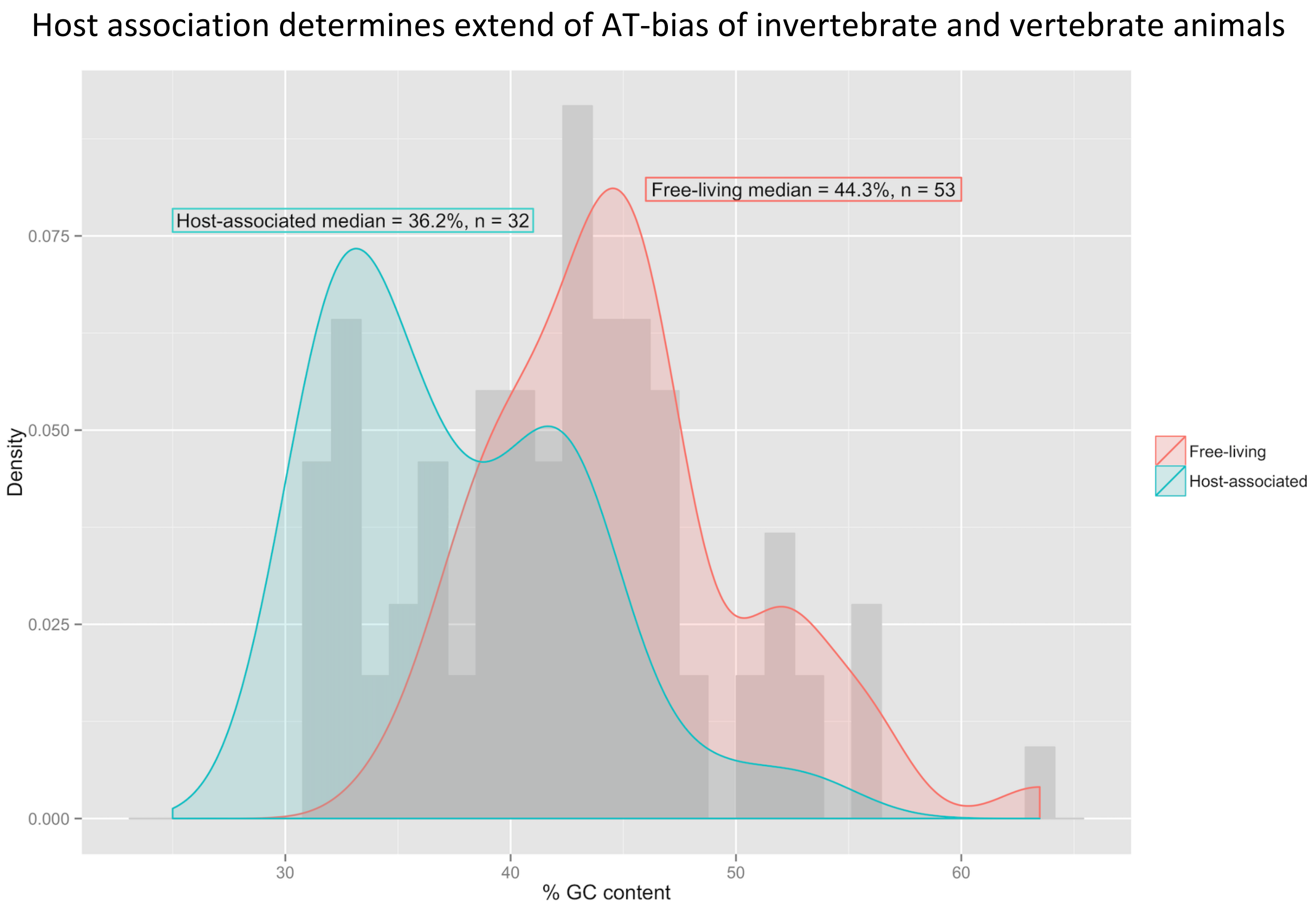

Fig. S5. A Host association determines the extend of AT-bias in 85 species of invertebrates and vertebrate animals based on the *Srp54K* gene, green host-associated, red free-living. **B**. Same analysis detailed for acariformes. **C.** Genome-wide codon usage bias seen in acariformes is more likely due to mutational bias leading to a higher frequency of codons containing adenine or thymine in the second and/or third position. ENCprime was used to calculate NC, the number of effective codons across coding sequence sets, as well as Ncp (Nc prime or Nc'), a measure of codon use corrected by the background mutation pattern. Corrections were performed using the nucleotide frequency of the third codon position for each respective gene under study. **D.** Mutation spectrum for Demodex demonstrates an AT-mutational bias. Frequency is for all mutation types discovered in RNA-sequencing data.

Intergenic distances and introns

	Acari							Araneae	Insecta
	Acariformes					Parsitiformes			Diptera
**Species***	***Demodex folliculorum***	*Aculops lycopersici*	*Tetranychus urticae*	*Dermatophagoides farinae*	*Sarcoptes scabiei*	*Ixodes scapularis*	*Galendromus occidentalis*	*Parasteatoda tepidariorum*	*Drosophila melanogaster*
mean intron length (bp)	**514**		502	693	147	2,920	625	4,324	1,067
median intron length (bp)	**79**	170	94	95	69	1,602	133	1,850	69
mean intron count per gene	**2.83**	0.30	2.35	1.93	2.03	3.33	4.61	6.2	2.94
mean exon length (bp)	**383**		319	456	367	197	266	198	400
median exon length (bp)	**180**		156	279	203	143	171	129	215
mean distance between CDS (bp) *	**2,429**		2,630	1,850	1,526	2,6615	8,028	44,743	5,668
median distance between CDS (bp) §	**1,293**	538	1,313	630	749	9,236	2,890	14,217	1,197

*Data is for coding sequences only and is for one CDS per gene. No filtering on raw numbers has been performed, so annotation quality may play a role in differences.
§ Distance between CDS annotations does not include distance between last CDS and scaffold ends.

Table S6. Acariformes have reduced their intergenic and intronic content compared to most arachnids, and a little more than Drosophila too. Intron numbers are lower in Acariformes, except for Demodex. Intron length is shorter on average in Acariformes as well, even when compared to *Galendromus occidentalis* and Drosophila. Intergenic distance is also lower in Acariformes than all else, though melanogaster is close. *D. melanogaster* sometimes is close to Acariformes based on median, but not mean, suggesting wider variation in metrics in *D. melanogaster*.

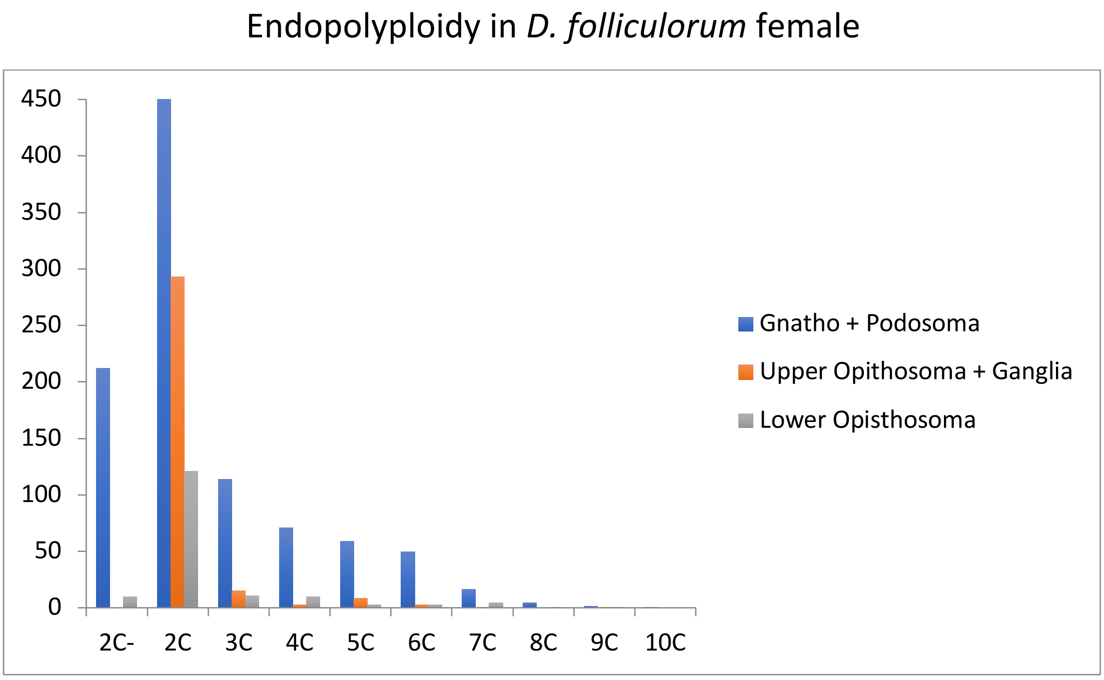

Figure S7. Ploidy distribution of nuclei in different body regions of *D. folliculorum* females. Abscissa: ploidy of nuclei, ordinate: number of nuclei per body region. 2C means diploid, 2C– indicates underreplication. The highest level of polyploidy observed was decaploid. The levels of polyploidy deviate from a geometric progression.

2C– presents underreplication where a premature stall takes place with less than one complete cycle of replication, unlike underreplication in nuclei of follicle cells and cells of salivary glands. In *D. folliculorum,* around 20 % of the diploid nuclei (some 222 nuclei) of adult females showed an 11 % deficiency in fluorescent intensity or nuclear content; these nuclei are called diploid *infer*.

The higher ploidy levels of *D. folliculorum* are mostly restricted to the gnathosomal and podosomal regions, with very little in the opisthosoma. These polyploid cells are mainly located in the internal organs as compared to the peripheral nuclei and the integument. Within the gnathosomal and podosomal regions, lower level polyploid nuclei are more prominent in the internal structures of the legs whereas the higher levels of polyploid nuclei such as hexaploid are standing out in the internal organs of the main body of the gnathosoma and podosoma.

2 Supplementary Items (SI)

Benchmarking Universal Single Copy Orthologs

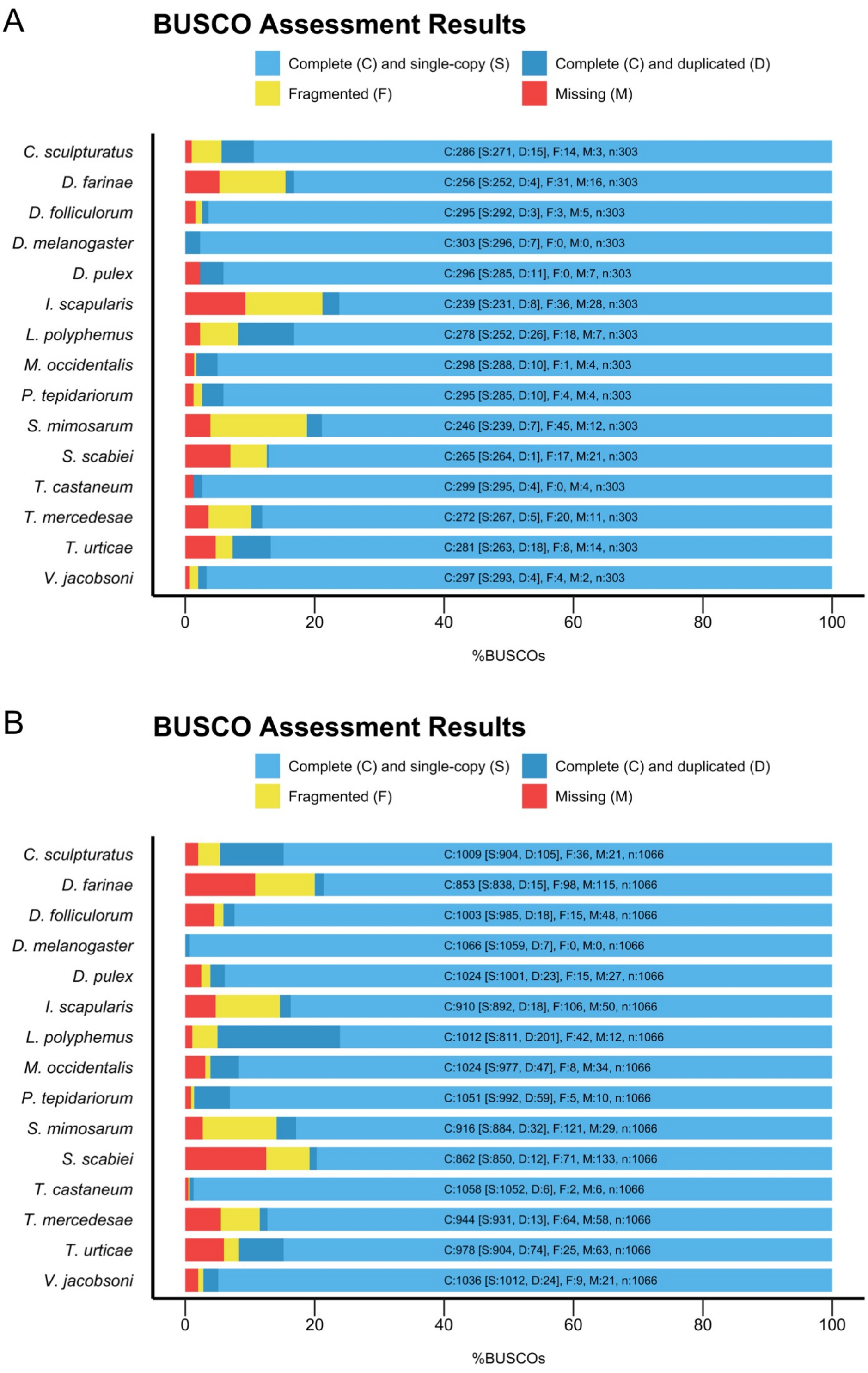

Figure SI 1. The *Demodex* genome is quite complete and well annotated. BUSCO results for genomes compared in this study. BUSCO was run for both eukaryote (**A**) and arthropod (**B**) sets of single copy orthologs, on released curated protein sets for each genome, filtered for longest isoform per gene where isoforms were annotated.

Length distribution of proteins in parasitic/endosymbiotic Demodex and free-living/plant-parasitic Tetranychus

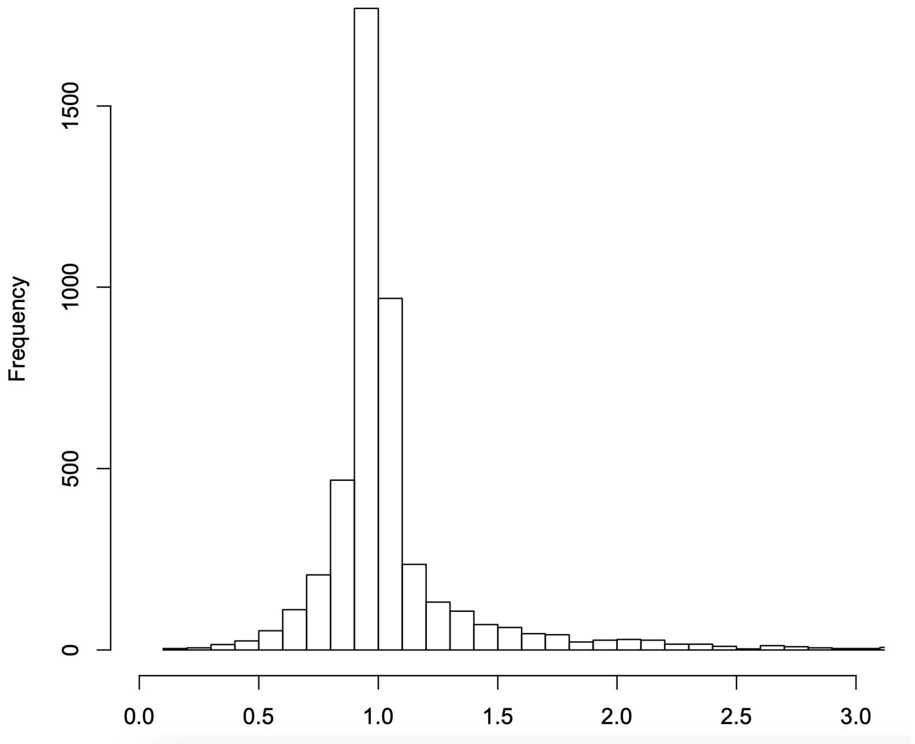

Figure SI 2. Zoomed-in length distribution of proteins in parasitic/endosymbiotic Demodex and free-living/plant-parasitic Tetranychus. The amino acid length of 4,582 one-to-one orthologues of Demodex was divided by the amino acid length of Tetranychus. All values on the abscissa below 1 represent proteins that are shorter in Demodex compared to Tetranychus and vice versa. The biggest problem here is that in fact two parasitic species are compared with each other. Sequences of closely related free-living species are still missing.

Assuming in a first approximation that pseudogenes will have lost 50 % or more of their protein length, only 50 Demodex proteins (1.1 %) meet this criterium, whereas 214 Tetranychus proteins (4.7 %) would qualify as pseudogenes.

The Asian long-horn beetle, *Anoplophora glabripennis*, has a large genome with 22,253 protein-coding genes and 66 pseudogenes, which represent 0.3 % (McKenna et al. 2016; Li et al. 2019).

Table SI 1

Probably pseudogenes of Demodex arranged according to Demodex AA length

ID	Dem. AA	Tet. AA	Function
DMDXFOLL_001444	110	988	ataxin-2 homolog
DMDXFOLL_008670	253	1,456	matricellular osteonectin sparc bm-40
DMDXFOLL_000412	134	735	glycerol-3-phosphate mitochondrial
DMDXFOLL_006536	72	361	large proline-rich BAG6
DMDXFOLL_002943	285	1,301	endothelial PAS domain-containing 1-like
DMDXFOLL_001752	208	912	WD repeat-containing 43-like
DMDXFOLL_003669	244	1,029	mediator of RNA polymerase II transcription subunit 8 isoform X2
DMDXFOLL_009322	121	497	GTP-binding RAD-like
DMDXFOLL_005420	559	2,239	multiple PDZ domain
DMDXFOLL_006675	391	1,469	arrestin domain-containing 3-like
DMDXFOLL_005513	147	475	spaetzle 4
DMDXFOLL_005099	125	395	steroid receptor seven- isoforms b c
DMDXFOLL_002884	203	616	5-hydroxytryptamine receptor 2A isoform X1
DMDXFOLL_006689	124	365	thromboxane-A synthase-like
DMDXFOLL_001855	149	438	targeting for Xklp2-B-like
DMDXFOLL_008375	146	429	tachykinin-like peptides receptor 99D
DMDXFOLL_006396	248	723	ataxin-3-like isoform X2
DMDXFOLL_005292	407	1,145	TBC1 domain family member 1 isoform X9
DMDXFOLL_010085	370	1,014	glutamate receptor delta-1-like
DMDXFOLL_004532	294	770	DNA helicase MCM8-like
DMDXFOLL_000519	130	339	RNA-binding motif X-linked 2
DMDXFOLL_004346	258	670	suppressor of lurcher 1
DMDXFOLL_001802	463	1,185	von Willebrand factor type EGF and pentraxin domain-containing 1
DMDXFOLL_005387	154	391	CCAAT enhancer-binding gamma-like
DMDXFOLL_002434	149	378	iron-sulfur cluster assembly 2 mitochondrial
DMDXFOLL_000594	242	593	alkylated DNA repair alkB homolog 8
DMDXFOLL_005803	178	436	tachykinin-like peptides receptor 99D
DMDXFOLL_010004	657	1,601	hepatocyte nuclear factor 6 isoform X3
DMDXFOLL_002872	321	779	sarcalumenin-like isoform X2
DMDXFOLL_007387	194	457	class A basic helix-loop-helix 15-like
DMDXFOLL_005646	127	293	NA
DMDXFOLL_006282	98	226	zinc finger CCCH domain-containing 41-like
DMDXFOLL_009539	188	422	potassium voltage-gated channel subfamily KQT member 2 isoform X1
DMDXFOLL_007218	775	1,739	low-density lipo
DMDXFOLL_010102	280	624	neuronal acetylcholine receptor subunit alpha-7 isoform X1
DMDXFOLL_009219	332	730	C-ets-1-like isoform X1
DMDXFOLL_004796	509	1,109	Shroom2 isoform X2
DMDXFOLL_008193	147	320	uncharacterized serine-rich -like
DMDXFOLL_006964	497	1,080	homeobox ceh-9
DMDXFOLL_000555	96	205	NA
DMDXFOLL_003743	229	488	lin-28 homolog

DMDXFOLL_008558	370	787	histone acetyltransferase KAT6A-like
DMDXFOLL_009331	230	481	forkhead box F1
DMDXFOLL_003624	107	222	serum response factor homolog B-like
DMDXFOLL_006709	217	450	von Willebrand factor C and EGF domain-containing
DMDXFOLL_002777	590	1,220	tRNA-dihydrouridine(47) synthase [NAD(P)(+)]-like
DMDXFOLL_008265	277	565	galactosamine (N-acetyl)-6-sulfate sulfatase
DMDXFOLL_003162	219	446	serine threonine-phosphatase 2A 55 kDa regulatory subunit B delta isoform isoform X3
DMDXFOLL_007010	411	837	homeobox HMX3

Tab. SI 2 Orthogroups of Demodex rapidly expanding

Orthogroup ID	Consensus KEGG K number	KEGG K title
OG0000063	NA	NA
OG0000064	NA	NA
OG0000121	NA	NA
OG0000129	NA	NA
OG0000132	NA	NA
OG0000256	K08592	SENP1; sentrin-specific protease 1 [EC:3.4.22.68]
OG0000283	K06704	ADAM10, CD156c; disintegrin and metalloproteinase domain-containing protein 10 [EC:3.4.24.81]
OG0000428	K15695	RNF103, KF1; E3 ubiquitin-protein ligase RNF103 [EC:2.3.2.27]

KEGG analysis of orthogroups expanding in Demodex compared to Acari.

Table SI 3

Minimal genome sizes and number of coding genes per taxonomic clade in the Pan-Arthropoda

			genome size	coding genes
Arthropoda				
Chelicerata				
Arachnida				
Acari				
Acariformes				
Sarcoptiformes				
Astigmata				
Sarcoptes scabiei	scabies mite	GCA_000828355.1	56.3	10,473
		GenBank median		9,830
Oribatida				
Steganacarus magnus	predatory mite		426.5	13,305
Endeostigmata				
Eriophycoidea				
Aculops lycopersici	tomato russet mite	GCA_015350385.1	32.5	10,263
Trombidiformes				
Prostigmata				
Brevipalpus yothersi	false spider mite	GCA_003956705.1	71.2	~ 16,000
Demodex folliculorum	**human follicle mite**	**awaiting assignment**	**51.6**	**9,707**
Parasitiformes				
Ixodida, Ixodoidea				
Ixodes scapularis	black-legged tick	Randall et al. (2018)	1,760.0	20,467
Mesostigmata, Monogynaspida				
Galendromus occidentalis	western predatory mite	GCA_000255335.1	151.7	11,944

Araneae				
Latrodectus hesperus	western black widow	GCA_000697925.2	1,234.0	?
Pseudoscorpiones				
Cordylochernes scorpioides	pseudoscorpion	QEEW00000000.1	2,807.1	?
Scorpiones				
Centruroides sculpturatus	bark scorpion	GCA_000671375.2	925.5	35,529
Merostomata				
Carcinoscorpius rotundicauda	mangrove horseshoe crab	GCA_011833715.1	1,669.0	25,985
Mandibulata				
Myriapoda				
Strigamia maritima	centipede	GCA_000239455.1	176.2	13,233
Pancrustacea				
Crustacea				
Branchiopoda				
Lepidurus arcticus	tadpole shrimp	GCA_003724045.1	73.1	10,718
Multicrustacea				
Oithona nana	copepod	GCA_900157175.1	85.0	?
Hexapoda				
Collembola				
Pseudachorutes palmiensis	springtail	VNWY00000000.1	77.1	?
Diplura				
Catajapyx aquilonaris	earwig-like entroph	JYFJ00000000.2	285.6	?
Insecta				
Clunio marinus	marine midge	GCA_900005825.1	85.5	22,620
Onychophora				
Euperipatoides rowelli	velvet worm	PXIH000000000.1	1,745.2	?
Tartigrada				

Ramazzottius varieornatus	water bear	GCA_001949185.1	55.8	22,994

Smallest genome size per taxonomic clade in Mbp based on assembly length, not on genome size estimates; vernacular name, and number of coding genes, where known; GenBank or INSDC code as of May 2021. The placement of the Eriophycoidea follows hypothesis 4 of Klimov et al. (2018) and is supported by Arribas et al. (2020) and Greenhalgh et al. (2020).

Table SI 4

Species used in genome analysis

Species	*Vernacular name*	*Accession/genome version)*	*Total assembly length (Mb)*
Demodex folliculorum	Human follicle mite	D_folliculorum_v1.1assembly_v02_annotations	51.5
Sarcoptes scabiei	Itch mite	GCA_000828355.1_SarSca1.0	56.3
Tetranychus urticae	Two spotted spider mite	20160622	90.8
Ixodes scapularis	Black-legged tick	IscaW1.4	1,765
Galendromus occidentalis	Western predatory mite	GCF_000255335.1_Mocc_1.0	152
Dermatophagoides farinae	American house dust mite	Dfarinae1.0	53.5
Parasteatoda tepidariorum	Common house spider	GCF_000365465.2_v2.0	1,445.4
Tropilaelaps mercedesae	Asian bee mite	T._mercedesae_v01	352.5
Stegodyphus mimosarum	Spider	GCA_000611955.2	2,738.7
Centruroides sculpturatus	Bark scorpion	GCA_000671375.2_Cexi_2.0	925.5
Varroa jacobsoni	Bee mite	GCA_002532875.1_vjacob_1.0	365.6
Daphnia pulex	Water flea	GCA_000187875.1_V1.0	197.2
Tribolium castaneum	Red flour beetle	GCF_000002335.3_Tcas5.2	165.9
Drosophila melanogaster	Fruit fly, vinegar fly	GCF_000001215.4_Release_6	143.7
Limulus polyphemus	Atlantic horseshoe crab	GCF_000517525.1	1,828.3

Species	No. scaffolds	No. proteins (longest isoform)	Scaffold N50 (Kb)	Scaffold L50	GC content	Data source
Demodex folliculorum	241	9,707	488	31	31.3	Current study
Sarcoptes scabiei	18,861	10,473	11.6	972	33.3	NCBI
Tetranychus urticae	640	19,104	2,993	10	32.3	bioinformatics.psb.ugent.be /gdb/tetranychus/
Ixodes scapularis	369,492	20,486	76.2	3,623	45.2	Vectorbase
Galendromus occidentalis	2,211	11,710	896.8	52	51.6	NCBI
Dermatophagoides farinae	515	16,376	186.3	85	30.6	Personal communication
Parasteatoda tepidariorum	16,533	18,601	4,055.4	94	32.9	NCBI
Tropilaelaps mercedesae	33,764	14,303	28.9	3,629	40.9	NCBI
Stegodyphus mimosarum	68,653	27,135	480.6	1,734	33.8	NCBI
Centruroides sculpturatus	8,338	24,591	537.5	523	31.4	NCBI
Varroa jacobsoni	4,881	10,739	233.8	482	40.9	NCBI
Daphnia pulex	5,186	30,590	642.1	75	42.4	NCBI
Tribolium castaneum	2,149	12,873	4.5	12	35.2	NCBI
Drosophila melanogaster	1,870	13,929	25.3	3	42.1	NCBI
Limulus polyphemus	286,793	22,873	254.1	1,712	34.5	NCBI

Table SI 5

Repeat content of mite genomes

	No. RepeatModeler families	Percent total repeats	Taxonomy
Aculops lycopersici	13	2.1 %	Acariforme
Demodex folliculorum	**164**	**7.2 %**	**Acariforme**
Sarcoptes scabiei	49	9.3 %	Acariforme
Tetranychus urticae	484	12.5 %	Acariforme
Dermatophagoides farinae	40	11.0 %	Acariforme
Metaseiulus occidentalis	593	9.3 %	Parasitiforme
Ixodes scapularis	3,497	41.7 %	Parasitiforme
Drosophila melanogaster	808	22.6 %	Insect

Table SI 6

Intergenic distances and introns

	Acari							Araneae	Insecta
	Acariformes					Parsitiformes			Diptera
**Species***	***Demodex folliculorum***	*Aculops lycopersici*	*Tetranychus urticae*	*Dermatophagoides farinae*	*Sarcoptes scabiei*	*Ixodes scapularis*	*Galendromus occidentalis*	*Parasteatoda tepidariorum*	*Drosophila melanogaster*
mean intron length (bp)	**514**		502	693	147	2,920	625	4,324	1,067
median intron length (bp)	**79**	170	94	95	69	1,602	133	1,850	69
mean intron count per gene	**2.83**	0.30	2.35	1.93	2.03	3.33	4.61	6.2	2.94
mean exon length (bp)	**383**		319	456	367	197	266	198	400
median exon length (bp)	**180**		156	279	203	143	171	129	215
mean distance between CDS (bp) *	**2,429**		2,630	1,850	1,526	2,6615	8,028	44,743	5,668
median distance between CDS (bp) §	**1,293**	538	1,313	630	749	9,236	2,890	14,217	1,197

*Data is for coding sequences only and is for one CDS per gene. No filtering on raw numbers has been performed, so annotation quality may play a role in differences.
§ Distance between CDS annotations does not include distance between last CDS and scaffold ends.

Acariformes have reduced their intergenic and intronic content compared to most arachnids, and a little more than Drosophila too. Intron numbers are lower in Acariformes, except for Demodex. Intron length is shorter on average in Acariformes as well even when compared to *Galendromus occidentalis* and Drosophila. Intergenic distance is also lower in Acariformes than all else, though Melanogaster is close. D. melanogaster sometimes is close to Acariformes based on median, but not mean, suggesting wider variation in metrics in *D. melanogaster*.

Within Acariformes, there is phylogenetic divergence: the Sarcoptiformes, *Sarcoptes scabiei,* have much shorter distances between CDSs; and slightly longer exons.

Host association determines extend of AT-bias of Acariformes

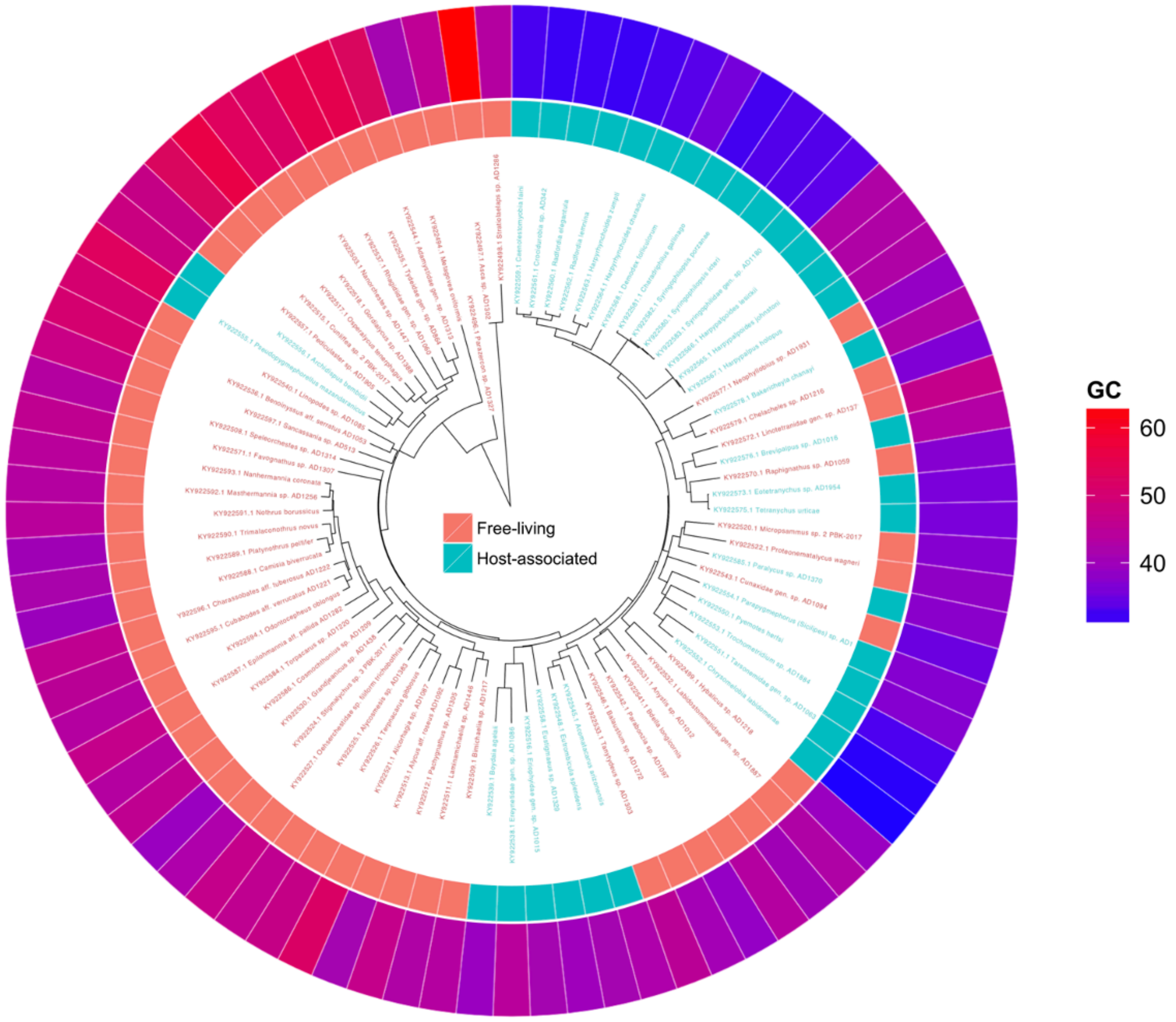

Fig. SI 3. Host association determines the extend of AT-bias in Acariformes based on the *Srp54K* gene, green host-associated, red free-living. Demodex is at a derived position at around 1 o'clock.

Genome-wide codon usage bias in Acariformes

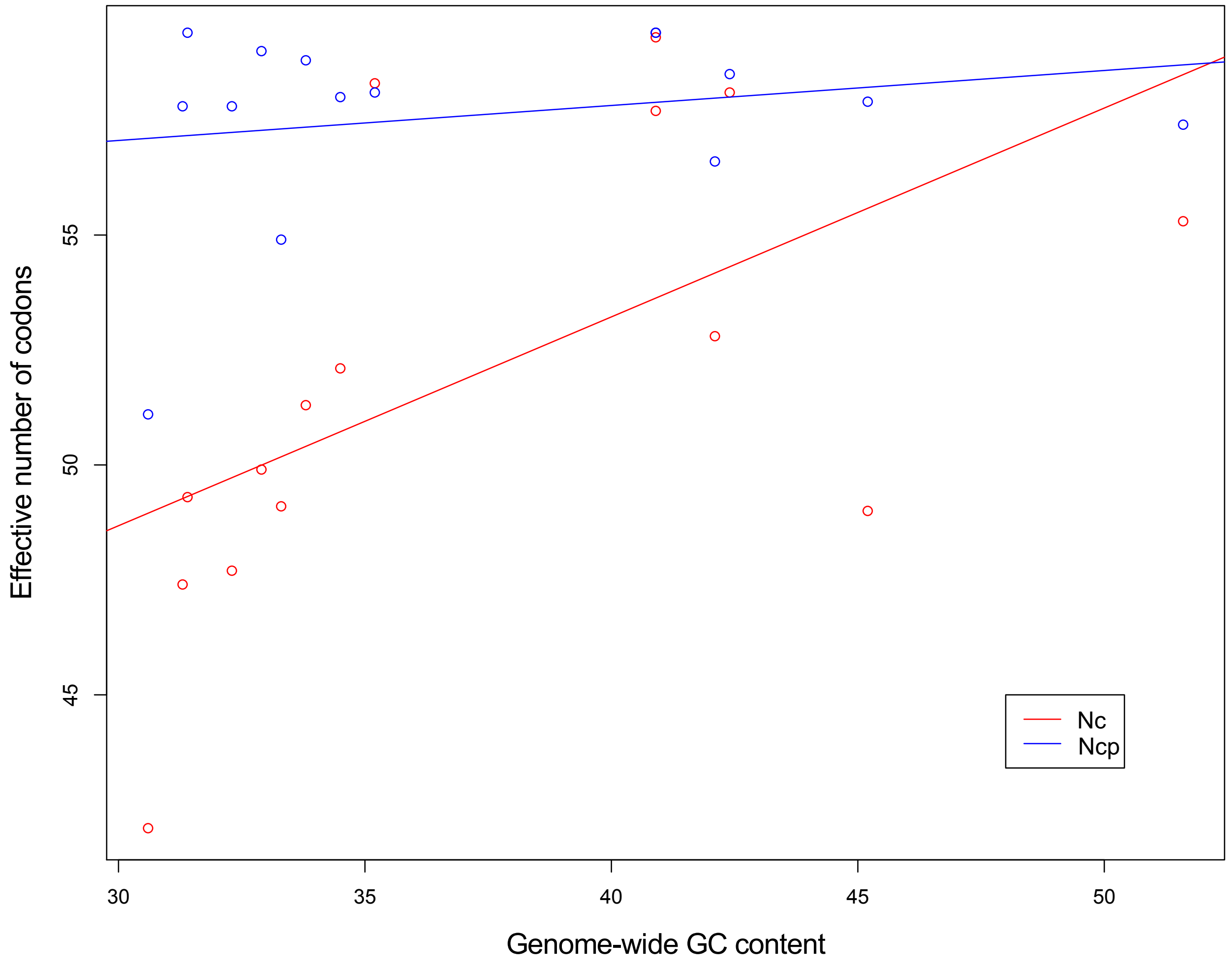

Fig. SI 4. Genome-wide codon usage bias seen in acariformes is more likely due to mutational bias leading to a higher frequency of codons containing adenine or thymine in the second and/or third position. ENCprime was used to calculate NC, the number of effective codons across coding sequence sets, as well as Ncp (Nc prime or Nc'), a measure of codon use corrected by the background mutation pattern. Corrections were performed using the nucleotide frequency of the third codon position for each respective gene under study.

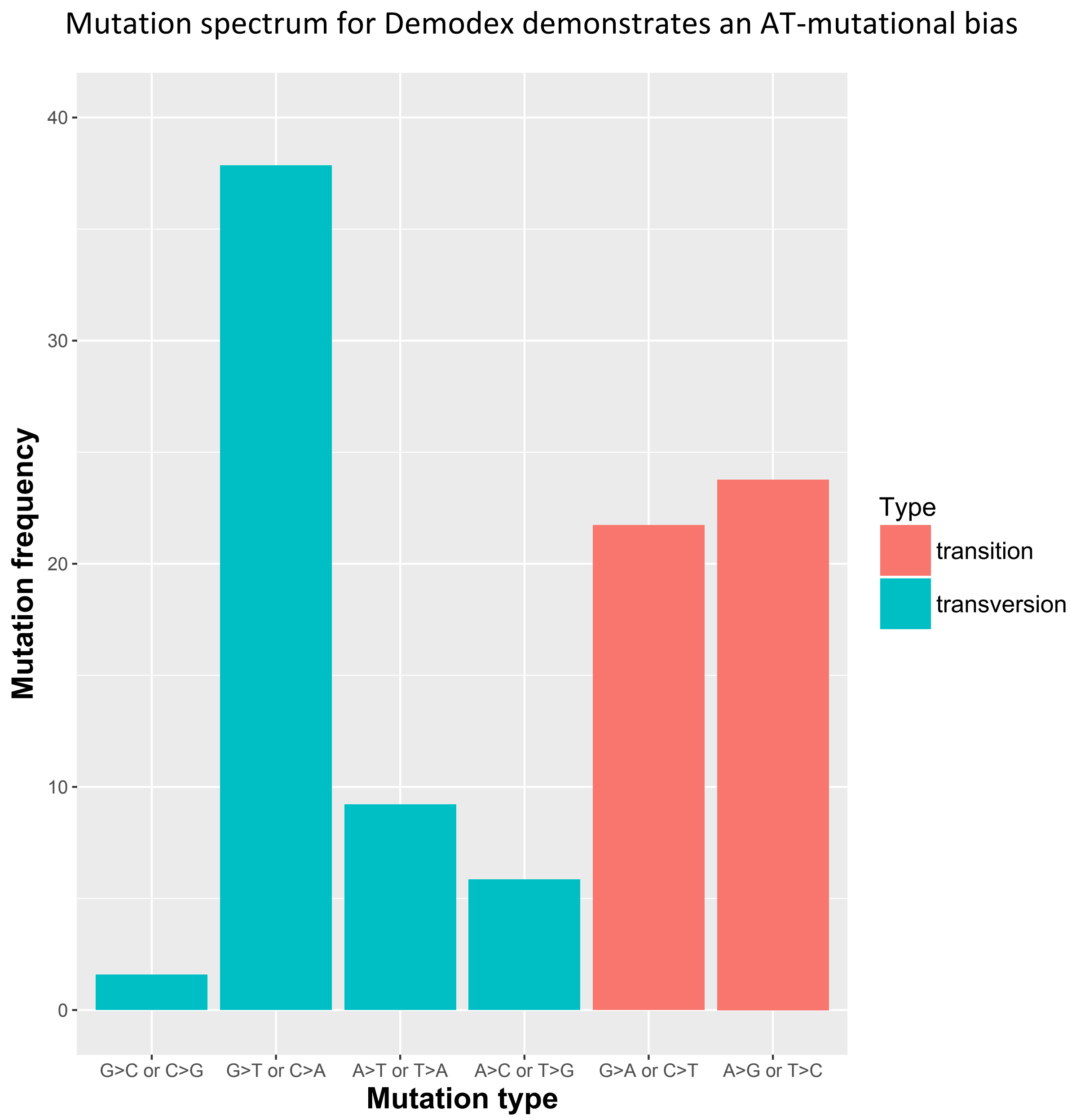

Fig. SI 5. Mutation spectrum for Demodex demonstrates an AT-mutational bias. Frequency is for all mutation types discovered in RNA-sequencing data.

Table SI 7

Orthogroups (OGs) of arthropod species

Species	# genes	# genes in OGs	# unassigned genes	% genes in OGs	% unassigned genes	# OGs containing species	% OGs containing species	# species-specific OG	# genes in species-specific OGs	% genes in species-specific OGs
Demodex folliculorum	**9,707**	**8,131**	**1,576**	**83.8**	**16.2**	**6,237**	**44.3**	**6**	**46**	**0.5**
Sarcoptes scabiei	10,473	8,440	2,033	80.6	19.4	6,279	44.6	7	24	0.2
Tetranychus urticae	19,104	11,939	7,165	62.5	37.5	6,254	44.4	55	1,111	5.8
Ixodes scapularis	20,486	14,054	6,432	68.6	31.4	7,730	54.9	19	95	0.5
Galendromus occidentalis	11,710	10,851	859	92.7	7.3	6,975	49.6	6	27	0.2
Dermatophagoides farinae	16,376	9,413	6,963	57.5	42.5	6,547	46.5	19	66	0.4
Parasteatoda tepidariorum	18,601	17,049	1,552	91.7	8.3	8,396	59.7	17	82	0.4
Tropilaelaps mercedesae	14,303	11,695	2,608	81.8	18.2	7,602	54	5	12	0.1
Stegodyphus mimosarum	27,135	20,921	6,214	77.1	22.9	8,842	62.8	13	61	0.2
Centruroides sculpturatus	24,591	21,700	2,891	88.2	11.8	8,498	60.4	13	102	0.4
Varroa jacobsoni	10,739	9,899	840	92.2	7.8	7,484	53.2	2	4	0
Daphnia pulex	30,590	17,221	13,369	56.3	43.7	7,335	52.1	120	972	3.2
Tribolium castaneum	12,873	11,182	1,691	86.9	13.1	7,313	52	27	177	1.4
Drosophila melanogaster	13,929	10,729	3,200	77.0	23.0	6,957	49.4	29	179	1.3
Limulus polyphemus	22,873	21,165	1,708	92.5	7.5	8,500	60.4	12	47	0.2

	Cscu	**Demo2**	Derm	Dmel2	Dpul2	IxSc2	Lpol2	Gale2	Ptep2	Sarc2
# genes	24591	**9707**	16376	13929	30590	20486	22873	11710	18601	10473
# genes in orthogroups	21700	**8131**	9413	10729	17221	14054	21165	10851	17049	8440
# unassigned genes	2891	**1576**	6963	3200	13369	6432	1708	859	1552	2033
% genes in orthogroups	88.2	**83.8**	57.5	77	56.3	68.6	92.5	92.7	91.7	80.6
% unassigned genes	11.8	**16.2**	42.5	23	43.7	31.4	7.5	7.3	8.3	19.4
# orthogroups containing species	8498	**6237**	6547	6957	7335	7730	8500	6975	8396	6279
% orthogroups containing species	60.4	**44.3**	46.5	49.4	52.1	54.9	60.4	49.6	59.7	44.6
# species-specific orthogroups	13	**6**	19	29	120	19	12	6	17	7
# in species-specific orthogroups	102	**46**	66	179	972	95	47	27	82	24
% genes in species-specific orthogroups	0.4	**0.5**	0.4	1.3	3.2	0.5	0.2	0.2	0.4	0.2

	Smim2	Tcas2	Tetr2	Tmer2	Vjac
# genes	27135	12873	19104	14303	10739
# genes in orthogroups	20921	11182	11939	11695	9899
# unassigned genes	6214	1691	7165	2608	840
% genes in orthogroups	77.1	86.9	62.5	81.8	92.2
% unassigned genes	22.9	13.1	37.5	18.2	7.8
# orthogroups containing species	8842	7313	6254	7602	7484
% orthogroups containing species	62.8	52	44.4	54	53.2
# species-specific orthogroups	13	27	55	5	2
# in species-specific orthogroups	61	177	1111	12	4
% genes in species-specific orthogroups	0.2	1.4	5.8	0.1	0

Table SI 8

Computational analysis of gene family evolution model parameters

Replicate	Topology type	Run parameters	Lambda estimate (All, Acariformes, Parasitiformes)	Mu estimate (All, Acariformes, Parasitiformes)	Log-Likelihood
1	unconstrained	Multi-lamdamu All/Acariformes/Parasitiformes	0.0009987,0.0005362,0.0006846	0.0003055,0.0006493,0.0005634	-129584
2	unconstrained	Multi-lamdamu All/Acariformes/Parasitiformes	0.0009987,0.0005362,0.0006846	0.0003055,0.0006493,0.0005634	-129584
3	unconstrained	Multi-lamdamu All/Acariformes/Parasitiformes	0.0009987,0.0005362,0.0006846	0.0003055,0.0006493,0.0005634	-129584
4	unconstrained	Multi-lamdamu All/Acariformes/Parasitiformes	0.0009987,0.0005362,0.0006846	0.0003055,0.0006493,0.0005634	-129584
5	unconstrained	Multi-lamdamu All/Acariformes/Parasitiformes	0.0009987,0.0005362,0.0006846	0.0003055,0.0006493,0.0005634	-129584
**1**	unconstrained	Multi-lamdamu All/Acariformes	0.0009329,0.0005435	0.0003671,0.0006394	-130372
2	unconstrained	Multi-lamdamu All/Acariformes	0.0009329,0.0005434	0.0003671,0.0006394	-130372
3	unconstrained	Multi-lamdamu All/Acariformes	0.0009329,0.0005434	0.0003671,0.0006394	-130372
4	unconstrained	Multi-lamdamu All/Acariformes	0.0009329,0.0005435	0.0003671,0.0006394	-130372
5	unconstrained	Multi-lamdamu All/Acariformes	0.0009329,0.0005435	0.0003671,0.0006394	-130372
**1**	unconstrained	Single lambdamu	0.0008591	0.0004289	-131605
2	unconstrained	Single lambdamu	0.000859	0.0004289	-131605
3	unconstrained	Single lambdamu	0.0008591	0.0004289	-131605
4	unconstrained	Single lambdamu	0.000859	0.0004289	-131605
5	unconstrained	Single lambdamu	0.0008636	0.0004273	-131606
**1**	unconstrained	Single lambda	0.0006764	NA	-133563
2	unconstrained	Single lambda	0.0006764	NA	-133563
3	unconstrained	Single lambda	0.0006764	NA	-133563
4	unconstrained	Single lambda	0.0006764	NA	-133563
5	unconstrained	Single lambda	0.0006764	NA	-133563
1	constrained	Multi-lamdamu All/Acariformes/Parasitiformes	0.0009399,0.0008018,0.0008803	0.0002468,0.0013128,0.0008238	-129336

2	constrained	Multi-lamdamu All/Acariformes/Parasitiformes	0.0009399,0.0008018,0.0008803	0.0002468,0.0013128,0.0008238	-129336
3	constrained	Multi-lamdamu All/Acariformes/Parasitiformes	0.0009399,0.0008018,0.0008802	0.0002468,0.0013127,0.0008238	-129336
4	constrained	Multi-lamdamu All/Acariformes/Parasitiformes	0.0009398,0.0008018,0.0008802	0.0002469,0.0013128,0.0008238	-129336
5	constrained	Multi-lamdamu All/Acariformes/Parasitiformes	0.0009399,0.0008018,0.0008802	0.0002468,0.0013127,0.0008238	-129336
**1**	constrained	Multi-lamdamu All/Acariformes	0.0009604,0.0008559	0.0003547,0.001197	-130603
2	constrained	Multi-lamdamu All/Acariformes	0.0009604,0.000856	0.0003547,0.001197	-130603
3	constrained	Multi-lamdamu All/Acariformes	0.0009604,0.000856	0.0003547,0.001197	-130603
4	constrained	Multi-lamdamu All/Acariformes	0.0009604,0.0008559	0.0003547,0.001197	-130603
5	constrained	Multi-lamdamu All/Acariformes	0.0009604,0.000856	0.0003547,0.001197	-130603
**1**	constrained	Single lambdamu	0.0009746	0.0004751	-132684
2	constrained	Single lambdamu	0.0009746	0.0004751	-132684
3	constrained	Single lambdamu	0.0009746	0.0004751	-132684
4	constrained	Single lambdamu	0.0009746	0.0004751	-132684
5	constrained	Single lambdamu	0.0009746	0.0004751	-132684
**1**	constrained	Single lambda	0.000761	NA	-134832
2	constrained	Single lambda	0.000761	NA	-134832
3	constrained	Single lambda	0.000761	NA	-134832
4	constrained	Single lambda	0.000761	NA	-134832
5	constrained	Single lambda	0.000761	NA	-134832

Constraint and unconstrained trees were used to examine gene family evolution with the program CAFE (Computational Analysis of Gene Family Evolution) v4.0 (De Bie et al. 2006). Several models of gene family evolution, testing different parameter combinations for the birth (Lambda) and death (Mu) probabilities of genes, were investigated. Each model run demonstrated consistent parameter estimates and log-likelihood values across replicates, suggesting the models successfully converged.

Computational Analysis of Gene Family Evolution lhtest histogram

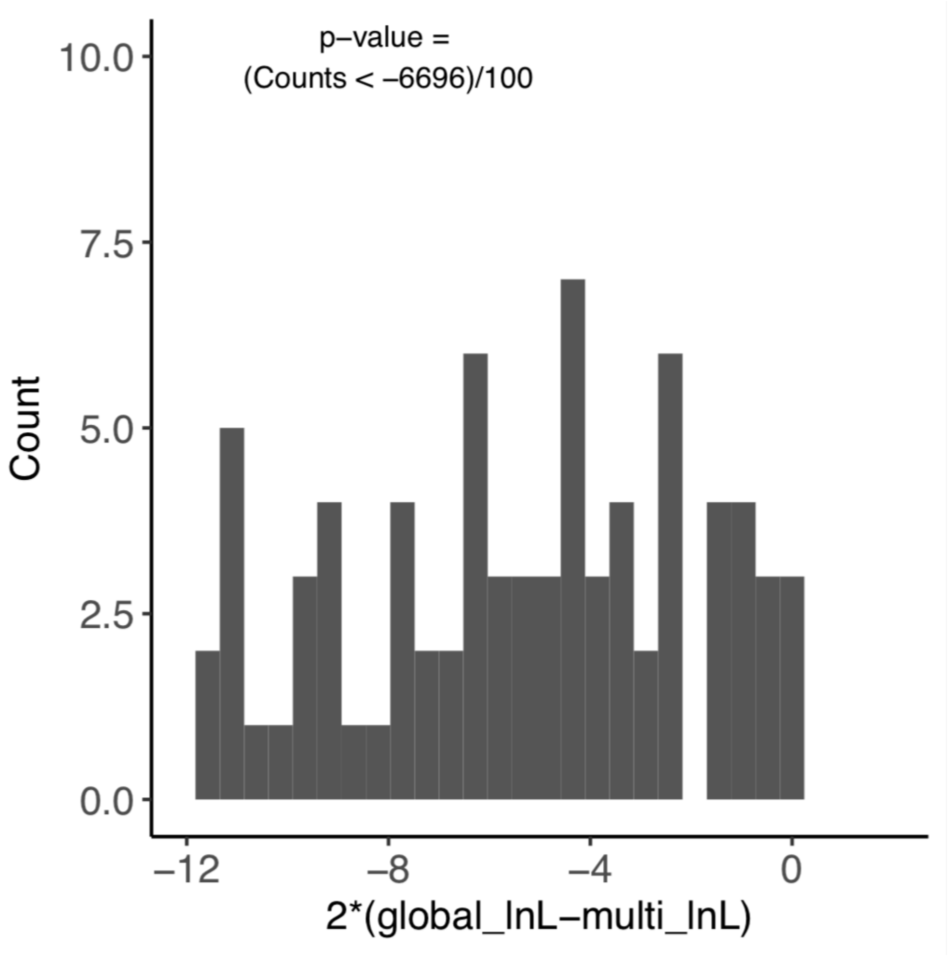

Figure SI 6. Selection was more frequently relaxed than intensified during Acariformes evolution. Constraint and unconstrained trees were used to examine gene family evolution with the program CAFE v4.0. A likelihood ratio comparison of a global-lambda model versus multi-lambda model (different birth and death parameters across the tree) demonstrated significant improvement in likelihood when the Acariforme and Parasitiforme branches were allowed separate parameter values (the test likelihood ratio fell in the far tail of the null distribution. For the computational analysis of gene family evolution model parameters, see Tab. SI 8.

Alternative IQtree topologies

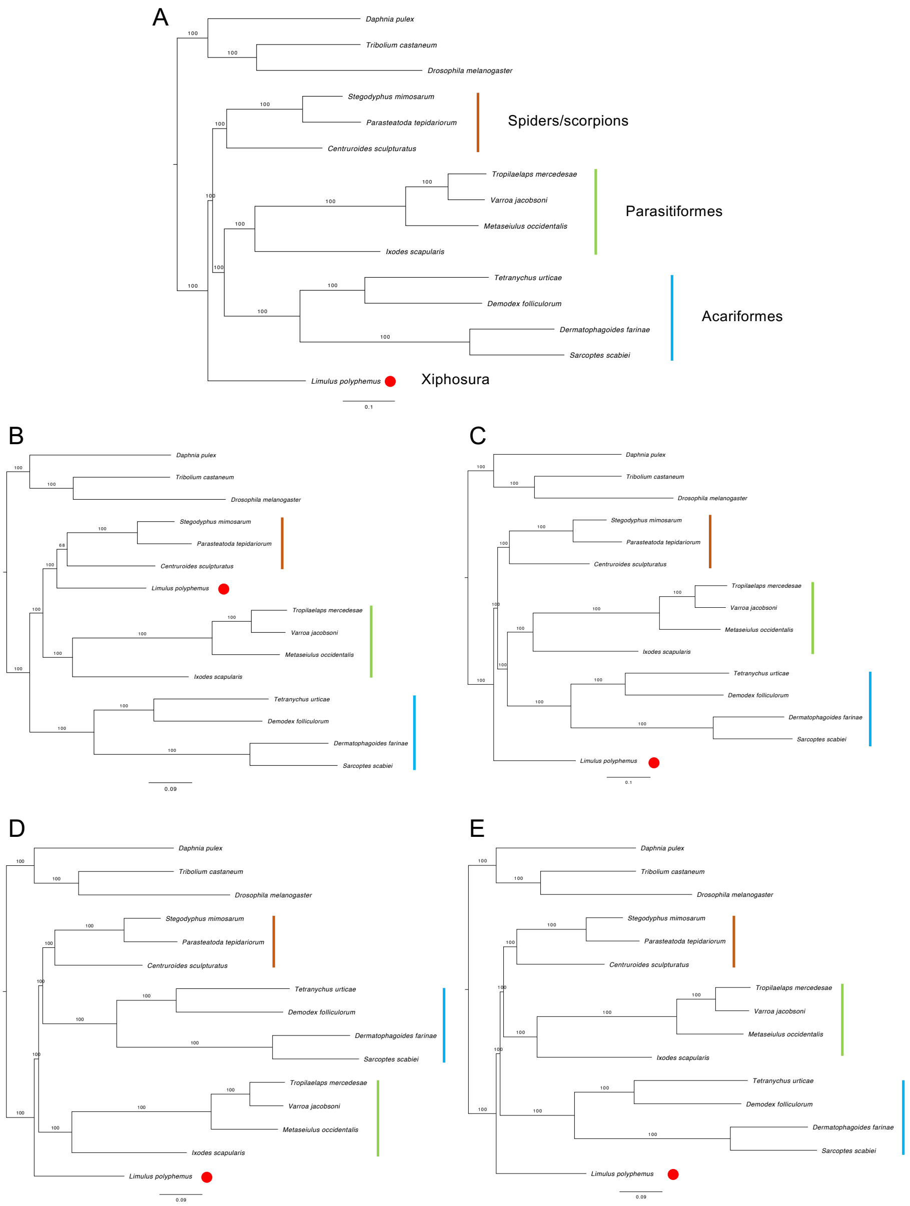

Figure SI 7. Alternative IQtree topologies: **A**: unconstrained, **B**: Outgroup constrained, **C**: Acariformes and Parasitiformes monophyletic, **D**: Spiders/scorpions and Acariformes monophyletic, and **E**: Spiders/scorpions and Parasitiformes monophyletic. Phylogenetic reconstruction using several different data sets of concatenated, unpartitioned loci, and partitioned single copy orthologs, including the slowest evolving sites and genes, respectively. However, like Sharma et al., every species tree recovered had the same topology, with Acariformes located at the base of arachnids, and with the horse shoe crab (*Limulus polyphemus*) grouped with spiders and scorpions (Sharma et al. 2014). Several different constrained topologies were tested against our data, with the most likely constraint tree, where *L. polyphemus* was constrained as outgroup to arachnids, resulting in a topology where Acariformes and Parasitiformes were monophyletic, and spiders and scorpions basal. Alternative topologies were significantly less likely compared to this one (p << 0.05; see below). Both the constraint tree and unconstrained tree were used to examine gene family evolution with the program CAFE. For values of individual trees, see corresponding table in Supplementary file.

Table SI 9

Gene families showing rapid contraction in Demodex

ontology	node_id	node_name	raw_p_overrep	FWER_ overrep	qvalue
biological_process	GO:0000003	**reproduction**	0.000245058	0.003	0.02725491
biological_process	GO:0006950	**response to stress**	0.000332377	0.003	0.02725491
biological_process	GO:0002376	**immune system process**	0.00066175	0.012	0.03124041
biological_process	GO:0006629	**lipid metabolic process**	0.000761961	0.021	0.03124041
biological_process	GO:0009056	catabolic process	0.006247036	0.167	0.20393705
biological_process	GO:0006790	sulfur compound metabolic process	0.007679403	0.19	0.20393705
biological_process	GO:0007568	aging	0.009022401	0.242	0.20393705
biological_process	GO:0048856	anatomical structure development	0.010106617	0.263	0.20393705
biological_process	GO:0042592	homeostatic process	0.012847617	0.29	0.20393705
biological_process	GO:0065008	regulation of biological quality	0.012847617	0.29	0.20393705
biological_process	GO:0032502	developmental process	0.015683056	0.33	0.20393705
biological_process	GO:0051179	localization	0.016923338	0.362	0.20393705
biological_process	GO:0019748	secondary metabolic process	0.017470317	0.382	0.20393705
biological_process	GO:0055085	transmembrane transport	0.019008746	0.417	0.20393705
biological_process	GO:0040011	locomotion	0.020443037	0.443	0.20393705
biological_process	GO:0006810	transport	0.022870499	0.47	0.20393705
biological_process	GO:0051234	establishment of localization	0.022870499	0.47	0.20393705
biological_process	GO:0044403	symbiont process	0.024870372	0.51	0.20393705
biological_process	GO:0044419	interspecies interaction between organisms	0.024870372	0.51	0.20393705
biological_process	GO:0051704	multi-organism process	0.024870372	0.51	0.20393705
biological_process	GO:0050896	response to stimulus	0.027503143	0.535	0.21478645
biological_process	GO:0044281	small molecule metabolic process	0.047013759	0.681	0.3504662
biological_process	GO:0071554	cell wall organization or biogenesis	0.06273701	0.789	0.44734216
biological_process	GO:0006928	movement of cell or subcellular component	0.073181626	0.85	0.46160718
biological_process	GO:0048870	cell motility	0.073181626	0.85	0.46160718
biological_process	GO:0051674	localization of cell	0.073181626	0.85	0.46160718
biological_process	GO:0008152	metabolic process	0.078813623	0.864	0.47871978
biological_process	GO:0030154	cell differentiation	0.093264571	0.874	0.54626392
biological_process	GO:0048869	cellular developmental process	0.102771437	0.889	0.56260801
biological_process	GO:0016192	vesicle-mediated transport	0.1029161	0.897	0.56260801
biological_process	GO:0007155	cell adhesion	0.117409573	0.917	0.60172406
biological_process	GO:0022610	biological adhesion	0.117409573	0.917	0.60172406
biological_process	GO:0007005	mitochondrion organization	0.127290235	0.93	0.6325939
biological_process	GO:0034330	cell junction organization	0.199451925	0.985	0.90304936
biological_process	GO:0065007	biological regulation	0.200081845	0.985	0.90304936
biological_process	GO:0022607	cellular component assembly	0.202729973	0.986	0.90304936
biological_process	GO:0051186	cofactor metabolic process	0.203736745	0.987	0.90304936
biological_process	GO:0051604	protein maturation	0.217343088	0.993	0.93800701
biological_process	GO:0034641	cellular nitrogen compound metabolic process	0.262660078	0.996	1
biological_process	GO:0006913	nucleocytoplasmic transport	0.302944918	0.999	1
biological_process	GO:0051169	nuclear transport	0.302944918	0.999	1

ontology	node_id	node_name	raw_p_overrep	FWER_ overrep	qvalue
biological_process	GO:0044238	primary metabolic process	0.309201542	0.999	1
biological_process	GO:0071704	organic substance metabolic process	0.309201542	0.999	1
biological_process	GO:0005975	carbohydrate metabolic process	0.318049044	0.999	1
biological_process	GO:0008283	cell proliferation	0.331043814	0.999	1
biological_process	GO:0007154	cell communication	0.342388669	0.999	1
biological_process	GO:0023052	signaling	0.342388669	0.999	1
biological_process	GO:0044085	cellular component biogenesis	0.351129691	0.999	1
biological_process	GO:0006259	DNA metabolic process	0.371485078	1	1
biological_process	GO:0008219	cell death	0.380568189	1	1
biological_process	GO:0006914	autophagy	0.422077586	1	1
biological_process	GO:0061919	process utilizing autophagic mechanism	0.422077586	1	1
biological_process	GO:0016043	cellular component organization	0.434496725	1	1
biological_process	GO:0090304	nucleic acid metabolic process	0.443986963	1	1
biological_process	GO:0048646	anatomical structure formation involved in morphogenesis	0.445251545	1	1
biological_process	GO:0021700	developmental maturation	0.465933427	1	1
biological_process	GO:0044237	cellular metabolic process	0.480261342	1	1
biological_process	GO:0000902	cell morphogenesis	0.501535874	1	1
biological_process	GO:0032989	cellular component morphogenesis	0.501535874	1	1
biological_process	GO:0061024	membrane organization	0.506579824	1	1
biological_process	GO:0006091	generation of precursor metabolites and energy	0.512850603	1	1
biological_process	GO:0009653	anatomical structure morphogenesis	0.513956066	1	1
biological_process	GO:0009987	cellular process	0.523977308	1	1
biological_process	GO:0007267	cell-cell signaling	0.548995919	1	1
biological_process	GO:0006139	nucleobase-containing compound metabolic process	0.55642788	1	1
biological_process	GO:0006725	cellular aromatic compound metabolic process	0.55642788	1	1
biological_process	GO:0046483	heterocycle metabolic process	0.55642788	1	1
biological_process	GO:1901360	organic cyclic compound metabolic process	0.55642788	1	1
biological_process	GO:0071840	cellular component organization or biogenesis	0.557358456	1	1
biological_process	GO:0007165	signal transduction	0.566280129	1	1
biological_process	GO:0050789	regulation of biological process	0.566280129	1	1
biological_process	GO:0050794	regulation of cellular process	0.566280129	1	1
biological_process	GO:0051716	cellular response to stimulus	0.566280129	1	1
biological_process	GO:0006396	RNA processing	0.569277826	1	1
biological_process	GO:0006397	mRNA processing	0.569277826	1	1
biological_process	GO:0016071	mRNA metabolic process	0.569277826	1	1
biological_process	GO:0006807	nitrogen compound metabolic process	0.575412062	1	1
biological_process	GO:0046907	intracellular transport	0.606402652	1	1
biological_process	GO:0051641	cellular localization	0.606402652	1	1
biological_process	GO:0051649	establishment of localization in cell	0.606402652	1	1
biological_process	GO:0006996	organelle organization	0.703884896	1	1
biological_process	GO:0044248	cellular catabolic process	0.708703423	1	1
biological_process	GO:0006082	organic acid metabolic process	0.713230583	1	1

ontology	node_id	node_name	raw_p_overrep	FWER_ overrep	qvalue
biological_process	GO:0006520	cellular amino acid metabolic process	0.713230583	1	1
biological_process	GO:0019752	carboxylic acid metabolic process	0.713230583	1	1
biological_process	GO:0043436	oxoacid metabolic process	0.713230583	1	1
biological_process	GO:0016070	RNA metabolic process	0.716211023	1	1
biological_process	GO:0050877	nervous system process	0.783465346	1	1
biological_process	GO:0007010	cytoskeleton organization	0.795695486	1	1
biological_process	GO:0003008	system process	0.813299838	1	1
biological_process	GO:0043933	protein-containing complex subunit organization	0.814288624	1	1
biological_process	GO:0065003	protein-containing complex assembly	0.814288624	1	1
biological_process	GO:0007275	multicellular organism development	0.817709874	1	1
biological_process	GO:0009790	embryo development	0.817709874	1	1
biological_process	GO:0032501	multicellular organismal process	0.818071026	1	1
biological_process	GO:0009058	biosynthetic process	0.830778115	1	1
biological_process	GO:0010467	gene expression	0.906486839	1	1
biological_process	GO:0007049	cell cycle	0.90925124	1	1
biological_process	GO:0043170	macromolecule metabolic process	0.948710932	1	1
biological_process	GO:1901564	organonitrogen compound metabolic process	0.951827599	1	1
biological_process	GO:0044260	cellular macromolecule metabolic process	0.963070644	1	1
biological_process	GO:0019538	protein metabolic process	0.980979132	1	1
biological_process	GO:0006464	cellular protein modification process	0.987889042	1	1
biological_process	GO:0036211	protein modification process	0.987889042	1	1
biological_process	GO:0043412	macromolecule modification	0.987889042	1	1
biological_process	GO:0044267	cellular protein metabolic process	0.99702158	1	1
biological_process	GO:0008150	biological_process	1	1	1
biological_process	GO:0071941	nitrogen cycle metabolic process	1	1	1
biological_process	GO:0032196	transposition	1	1	1
biological_process	GO:0007009	plasma membrane organization	1	1	1
biological_process	GO:0010256	endomembrane system organization	1	1	1
biological_process	GO:0007034	vacuolar transport	1	1	1
biological_process	GO:0030198	extracellular matrix organization	1	1	1
biological_process	GO:0043062	extracellular structure organization	1	1	1
biological_process	GO:0003013	circulatory system process	1	1	1
biological_process	GO:0030705	cytoskeleton-dependent intracellular transport	1	1	1
biological_process	GO:0043473	pigmentation	1	1	1
biological_process	GO:0006457	protein folding	1	1	1
biological_process	GO:0006605	protein targeting	1	1	1
biological_process	GO:0006886	intracellular protein transport	1	1	1
biological_process	GO:0008104	protein localization	1	1	1
biological_process	GO:0015031	protein transport	1	1	1
biological_process	GO:0015833	peptide transport	1	1	1
biological_process	GO:0033036	macromolecule localization	1	1	1
biological_process	GO:0034613	cellular protein localization	1	1	1
biological_process	GO:0042886	amide transport	1	1	1

ontology	node_id	node_name	raw_p_overrep	FWER_ overrep	qvalue
biological_process	GO:0045184	establishment of protein localization	1	1	1
biological_process	GO:0070727	cellular macromolecule localization	1	1	1
biological_process	GO:0071702	organic substance transport	1	1	1
biological_process	GO:0071705	nitrogen compound transport	1	1	1
biological_process	GO:0022618	ribonucleoprotein complex assembly	1	1	1
biological_process	GO:0034622	cellular protein-containing complex assembly	1	1	1
biological_process	GO:0071826	ribonucleoprotein complex subunit organization	1	1	1
biological_process	GO:0006399	tRNA metabolic process	1	1	1
biological_process	GO:0034660	ncRNA metabolic process	1	1	1
biological_process	GO:0000280	nuclear division	1	1	1
biological_process	GO:0022402	cell cycle process	1	1	1
biological_process	GO:0048285	organelle fission	1	1	1
biological_process	GO:0140014	mitotic nuclear division	1	1	1
biological_process	GO:1903047	mitotic cell cycle process	1	1	1
biological_process	GO:0007059	chromosome segregation	1	1	1
biological_process	GO:0019439	aromatic compound catabolic process	1	1	1
biological_process	GO:0034655	nucleobase-containing compound catabolic process	1	1	1
biological_process	GO:0044270	cellular nitrogen compound catabolic process	1	1	1
biological_process	GO:0046700	heterocycle catabolic process	1	1	1
biological_process	GO:1901361	organic cyclic compound catabolic process	1	1	1
biological_process	GO:1901575	organic substance catabolic process	1	1	1
biological_process	GO:0051301	cell division	1	1	1
biological_process	GO:0042254	ribosome biogenesis	1	1	1
biological_process	GO:0022613	ribonucleoprotein complex biogenesis	1	1	1
biological_process	GO:0006412	translation	1	1	1
biological_process	GO:0006518	peptide metabolic process	1	1	1
biological_process	GO:0009059	macromolecule biosynthetic process	1	1	1
biological_process	GO:0034645	cellular macromolecule biosynthetic process	1	1	1
biological_process	GO:0043043	peptide biosynthetic process	1	1	1
biological_process	GO:0043603	cellular amide metabolic process	1	1	1
biological_process	GO:0043604	amide biosynthetic process	1	1	1
biological_process	GO:0044249	cellular biosynthetic process	1	1	1
biological_process	GO:0044271	cellular nitrogen compound biosynthetic process	1	1	1
biological_process	GO:1901566	organonitrogen compound biosynthetic process	1	1	1
biological_process	GO:1901576	organic substance biosynthetic process	1	1	1
biological_process	GO:0040007	growth	1	1	1
biological_process	GO:0000278	mitotic cell cycle	1	1	1
biological_process	GO:0051276	chromosome organization	1	1	1

Table SI 10

Gene families showing rapid contraction in the Acariformes

ontology	node_id	node_name	raw_p_overrep	FWER_overrep	qvalue
biological_process	GO:0000003	**reproduction**	0.00075246	0.026	0.110618425
biological_process	GO:0040011	**locomotion**	0.002161383	0.068	0.110618425
biological_process	GO:0051179	localization	0.003618482	0.114	0.110618425
biological_process	GO:0032502	developmental process	0.005302367	0.164	0.110618425
biological_process	GO:0006790	sulfur compound metabolic process	0.006286359	0.206	0.110618425
biological_process	GO:0002376	immune system process	0.006397885	0.208	0.110618425
biological_process	GO:0048856	anatomical structure development	0.006549997	0.212	0.110618425
biological_process	GO:0055085	transmembrane transport	0.007086009	0.224	0.110618425
biological_process	GO:0007568	aging	0.007715849	0.245	0.110618425
biological_process	GO:0006928	movement of cell or subcellular component	0.008094031	0.266	0.110618425
biological_process	GO:0048870	cell motility	0.008094031	0.266	0.110618425
biological_process	GO:0051674	localization of cell	0.008094031	0.266	0.110618425
biological_process	GO:0006950	response to stress	0.008844222	0.293	0.111573268
biological_process	GO:0006810	transport	0.016701402	0.384	0.182601993
biological_process	GO:0051234	establishment of localization	0.016701402	0.384	0.182601993
biological_process	GO:0030154	cell differentiation	0.019485944	0.429	0.199730922
biological_process	GO:0048869	cellular developmental process	0.02328447	0.504	0.216975628
biological_process	GO:0006629	lipid metabolic process	0.023814398	0.511	0.216975628
biological_process	GO:0007267	cell-cell signaling	0.042436087	0.73	0.34443338
biological_process	GO:0042592	homeostatic process	0.044104274	0.738	0.34443338
biological_process	GO:0065008	regulation of biological quality	0.044104274	0.738	0.34443338
biological_process	GO:0019748	secondary metabolic process	0.052304225	0.788	0.38990422
biological_process	GO:0051604	protein maturation	0.071581325	0.867	0.459538925
biological_process	GO:0044403	symbiont process	0.072853732	0.874	0.459538925
biological_process	GO:0044419	interspecies interaction between organisms	0.072853732	0.874	0.459538925
biological_process	GO:0051704	multi-organism process	0.072853732	0.874	0.459538925
biological_process	GO:0008219	cell death	0.086779075	0.913	0.514498092
biological_process	GO:0007155	cell adhesion	0.090978321	0.919	0.514498092
biological_process	GO:0022610	biological adhesion	0.090978321	0.919	0.514498092
biological_process	GO:0032196	transposition	0.102562725	0.939	0.556515136
biological_process	GO:0050896	response to stimulus	0.105194934	0.941	0.556515136
biological_process	GO:0071554	cell wall organization or biogenesis	0.110629151	0.953	0.566974397
biological_process	GO:0048646	anatomical structure formation involved in morphogenesis	0.130916311	0.97	0.650614396
biological_process	GO:0009056	catabolic process	0.154669718	0.979	0.746053932
biological_process	GO:0006259	DNA metabolic process	0.202384132	0.991	0.946668035
biological_process	GO:0009653	anatomical structure morphogenesis	0.209021269	0.991	0.946668035
biological_process	GO:0007154	cell communication	0.219349911	0.993	0.946668035
biological_process	GO:0023052	signaling	0.219349911	0.993	0.946668035
biological_process	GO:0016192	vesicle-mediated transport	0.226951806	0.997	0.954361442
biological_process	GO:0008152	metabolic process	0.264042327	0.998	1

ontology	node_id	node_name	raw_p_overrep	FWER_overrep	qvalue
biological_process	GO:0044281	small molecule metabolic process	0.276655906	0.998	1
biological_process	GO:0050877	nervous system process	0.279767473	0.998	1
biological_process	GO:0021700	developmental maturation	0.308489507	1	1
biological_process	GO:0007005	mitochondrion organization	0.310156903	1	1
biological_process	GO:0034330	cell junction organization	0.33138287	1	1
biological_process	GO:0003008	system process	0.337449441	1	1
biological_process	GO:0065007	biological regulation	0.339045724	1	1
biological_process	GO:0005975	carbohydrate metabolic process	0.341866703	1	1
biological_process	GO:0022607	cellular component assembly	0.345528291	1	1
biological_process	GO:0000902	cell morphogenesis	0.353074755	1	1
biological_process	GO:0032989	cellular component morphogenesis	0.353074755	1	1
biological_process	GO:0008283	cell proliferation	0.359300459	1	1
biological_process	GO:0034641	cellular nitrogen compound metabolic process	0.373643909	1	1
biological_process	GO:0043473	pigmentation	0.447726676	1	1
biological_process	GO:0051186	cofactor metabolic process	0.450150205	1	1
biological_process	GO:0090304	nucleic acid metabolic process	0.451635461	1	1
biological_process	GO:0016043	cellular component organization	0.45512102	1	1
biological_process	GO:0006913	nucleocytoplasmic transport	0.479539532	1	1
biological_process	GO:0051169	nuclear transport	0.479539532	1	1
biological_process	GO:0044085	cellular component biogenesis	0.573067885	1	1
biological_process	GO:0007275	multicellular organism development	0.578845652	1	1
biological_process	GO:0009790	embryo development	0.578845652	1	1
biological_process	GO:0006139	nucleobase-containing compound metabolic process	0.600457204	1	1
biological_process	GO:0006725	cellular aromatic compound metabolic process	0.600457204	1	1
biological_process	GO:0046483	heterocycle metabolic process	0.600457204	1	1
biological_process	GO:1901360	organic cyclic compound metabolic process	0.600457204	1	1
biological_process	GO:0071840	cellular component organization or biogenesis	0.617964777	1	1
biological_process	GO:0044237	cellular metabolic process	0.624727844	1	1
biological_process	GO:0006914	autophagy	0.629238041	1	1
biological_process	GO:0061919	process utilizing autophagic mechanism	0.629238041	1	1
biological_process	GO:0032501	multicellular organismal process	0.642699034	1	1
biological_process	GO:0007059	chromosome segregation	0.64915975	1	1
biological_process	GO:0044238	primary metabolic process	0.654166095	1	1
biological_process	GO:0071704	organic substance metabolic process	0.654166095	1	1
biological_process	GO:0006807	nitrogen compound metabolic process	0.657432121	1	1
biological_process	GO:0040007	growth	0.692047958	1	1
biological_process	GO:0007165	signal transduction	0.692122475	1	1
biological_process	GO:0050789	regulation of biological process	0.692122475	1	1
biological_process	GO:0050794	regulation of cellular process	0.692122475	1	1
biological_process	GO:0051716	cellular response to stimulus	0.692122475	1	1
biological_process	GO:0061024	membrane organization	0.721474951	1	1
biological_process	GO:0006091	generation of precursor metabolites and energy	0.727847321	1	1

ontology	node_id	node_name	raw_p_overrep	FWER_overrep	qvalue
biological_process	GO:0007010	cytoskeleton organization	0.775744055	1	1
biological_process	GO:0009987	cellular process	0.779030697	1	1
biological_process	GO:0006996	organelle organization	0.781866266	1	1
biological_process	GO:0006396	RNA processing	0.782196866	1	1
biological_process	GO:0006397	mRNA processing	0.782196866	1	1
biological_process	GO:0016071	mRNA metabolic process	0.782196866	1	1
biological_process	GO:0009058	biosynthetic process	0.795244716	1	1
biological_process	GO:0007049	cell cycle	0.79549112	1	1
biological_process	GO:0043933	protein-containing complex subunit organization	0.802546635	1	1
biological_process	GO:0065003	protein-containing complex assembly	0.802546635	1	1
biological_process	GO:0046907	intracellular transport	0.814975973	1	1
biological_process	GO:0051641	cellular localization	0.814975973	1	1
biological_process	GO:0051649	establishment of localization in cell	0.814975973	1	1
biological_process	GO:0044248	cellular catabolic process	0.892679117	1	1
biological_process	GO:0006082	organic acid metabolic process	0.895678378	1	1
biological_process	GO:0006520	cellular amino acid metabolic process	0.895678378	1	1
biological_process	GO:0019752	carboxylic acid metabolic process	0.895678378	1	1
biological_process	GO:0043436	oxoacid metabolic process	0.895678378	1	1
biological_process	GO:0016070	RNA metabolic process	0.897632146	1	1
biological_process	GO:0010467	gene expression	0.924471813	1	1
biological_process	GO:0051276	chromosome organization	0.958266433	1	1
biological_process	GO:0043170	macromolecule metabolic process	0.962591317	1	1
biological_process	GO:0044260	cellular macromolecule metabolic process	0.978828947	1	1
biological_process	GO:1901564	organonitrogen compound metabolic process	0.98873187	1	1
biological_process	GO:0019538	protein metabolic process	0.992942032	1	1
biological_process	GO:0006464	cellular protein modification process	0.996694521	1	1
biological_process	GO:0036211	protein modification process	0.996694521	1	1
biological_process	GO:0043412	macromolecule modification	0.996694521	1	1
biological_process	GO:0044267	cellular protein metabolic process	0.999655784	1	1
biological_process	GO:0008150	biological_process	1	1	1
biological_process	GO:0071941	nitrogen cycle metabolic process	1	1	1
biological_process	GO:0007009	plasma membrane organization	1	1	1
biological_process	GO:0010256	endomembrane system organization	1	1	1
biological_process	GO:0007034	vacuolar transport	1	1	1
biological_process	GO:0030198	extracellular matrix organization	1	1	1
biological_process	GO:0043062	extracellular structure organization	1	1	1
biological_process	GO:0003013	circulatory system process	1	1	1
biological_process	GO:0030705	cytoskeleton-dependent intracellular transport	1	1	1
biological_process	GO:0006457	protein folding	1	1	1
biological_process	GO:0006605	protein targeting	1	1	1
biological_process	GO:0006886	intracellular protein transport	1	1	1
biological_process	GO:0008104	protein localization	1	1	1

ontology	node_id	node_name	raw_p_overrep	FWER_overrep	qvalue
biological_process	GO:0015031	protein transport	1	1	1
biological_process	GO:0015833	peptide transport	1	1	1
biological_process	GO:0033036	macromolecule localization	1	1	1
biological_process	GO:0034613	cellular protein localization	1	1	1
biological_process	GO:0042886	amide transport	1	1	1
biological_process	GO:0045184	establishment of protein localization	1	1	1
biological_process	GO:0070727	cellular macromolecule localization	1	1	1
biological_process	GO:0071702	organic substance transport	1	1	1
biological_process	GO:0071705	nitrogen compound transport	1	1	1
biological_process	GO:0022618	ribonucleoprotein complex assembly	1	1	1
biological_process	GO:0034622	cellular protein-containing complex assembly	1	1	1
biological_process	GO:0071826	ribonucleoprotein complex subunit organization	1	1	1
biological_process	GO:0006399	tRNA metabolic process	1	1	1
biological_process	GO:0034660	ncRNA metabolic process	1	1	1
biological_process	GO:0000280	nuclear division	1	1	1
biological_process	GO:0022402	cell cycle process	1	1	1
biological_process	GO:0048285	organelle fission	1	1	1
biological_process	GO:0140014	mitotic nuclear division	1	1	1
biological_process	GO:1903047	mitotic cell cycle process	1	1	1
biological_process	GO:0019439	aromatic compound catabolic process	1	1	1
biological_process	GO:0034655	nucleobase-containing compound catabolic process	1	1	1
biological_process	GO:0044270	cellular nitrogen compound catabolic process	1	1	1
biological_process	GO:0046700	heterocycle catabolic process	1	1	1
biological_process	GO:1901361	organic cyclic compound catabolic process	1	1	1
biological_process	GO:1901575	organic substance catabolic process	1	1	1
biological_process	GO:0051301	cell division	1	1	1
biological_process	GO:0042254	ribosome biogenesis	1	1	1
biological_process	GO:0022613	ribonucleoprotein complex biogenesis	1	1	1
biological_process	GO:0006412	translation	1	1	1
biological_process	GO:0006518	peptide metabolic process	1	1	1
biological_process	GO:0009059	macromolecule biosynthetic process	1	1	1
biological_process	GO:0034645	cellular macromolecule biosynthetic process	1	1	1
biological_process	GO:0043043	peptide biosynthetic process	1	1	1
biological_process	GO:0043603	cellular amide metabolic process	1	1	1
biological_process	GO:0043604	amide biosynthetic process	1	1	1
biological_process	GO:0044249	cellular biosynthetic process	1	1	1
biological_process	GO:0044271	cellular nitrogen compound biosynthetic process	1	1	1
biological_process	GO:1901566	organonitrogen compound biosynthetic process	1	1	1
biological_process	GO:1901576	organic substance biosynthetic process	1	1	1
biological_process	GO:0000278	mitotic cell cycle	1	1	1

Table SI 11

Gene families showing rapid expansion in the Acariformes

ontology	node_id	node_name	raw_p_overrep	FWER_overrep	qvalue
biological_process	GO:0030154	**cell differentiation**	0.000758826	0.026	0.075680876
biological_process	GO:0048869	**cellular developmental process**	0.000922938	0.028	0.075680876
biological_process	GO:0048856	**anatomical structure development**	0.001987254	0.067	0.108636548
biological_process	GO:0032502	**developmental process**	0.003344292	0.092	0.112633924
biological_process	GO:0006629	**lipid metabolic process**	0.003438321	0.093	0.112633924
biological_process	GO:0005975	carbohydrate metabolic process	0.004120753	0.121	0.112633924
biological_process	GO:0006259	DNA metabolic process	0.006809496	0.179	0.159536763
biological_process	GO:0044238	primary metabolic process	0.011757261	0.268	0.214243415
biological_process	GO:0071704	organic substance metabolic process	0.011757261	0.268	0.214243415
biological_process	GO:0009987	cellular process	0.024478493	0.487	0.401447277
biological_process	GO:0000003	reproduction	0.039277058	0.629	0.58558523
biological_process	GO:0002376	immune system process	0.059950675	0.785	0.711049681
biological_process	GO:0071554	cell wall organization or biogenesis	0.060395654	0.8	0.711049681
biological_process	GO:0007568	aging	0.06495895	0.832	0.711049681
biological_process	GO:0007009	plasma membrane organization	0.069370701	0.843	0.711049681
biological_process	GO:0010256	endomembrane system organization	0.069370701	0.843	0.711049681
biological_process	GO:0090304	nucleic acid metabolic process	0.078010571	0.86	0.728374061
biological_process	GO:0008152	metabolic process	0.108006837	0.897	0.728374061
biological_process	GO:0050896	response to stimulus	0.108619193	0.901	0.728374061
biological_process	GO:0009653	anatomical structure morphogenesis	0.109559465	0.903	0.728374061
biological_process	GO:0007155	cell adhesion	0.109959986	0.909	0.728374061
biological_process	GO:0022610	biological adhesion	0.109959986	0.909	0.728374061
biological_process	GO:0044260	cellular macromolecule metabolic process	0.111350058	0.911	0.728374061
biological_process	GO:0006950	response to stress	0.117627012	0.918	0.728374061
biological_process	GO:0021700	developmental maturation	0.118511666	0.922	0.728374061
biological_process	GO:0006139	nucleobase-containing compound metabolic process	0.132667593	0.946	0.728374061
biological_process	GO:0006725	cellular aromatic compound metabolic process	0.132667593	0.946	0.728374061
biological_process	GO:0046483	heterocycle metabolic process	0.132667593	0.946	0.728374061
biological_process	GO:1901360	organic cyclic compound metabolic process	0.132667593	0.946	0.728374061
biological_process	GO:0009056	catabolic process	0.144100466	0.96	0.728374061
biological_process	GO:0006091	generation of precursor metabolites and energy	0.147603293	0.965	0.728374061
biological_process	GO:0042592	homeostatic process	0.158670502	0.97	0.728374061
biological_process	GO:0065008	regulation of biological quality	0.158670502	0.97	0.728374061
biological_process	GO:0007154	cell communication	0.165799716	0.972	0.728374061
biological_process	GO:0023052	signaling	0.165799716	0.972	0.728374061
biological_process	GO:0065007	biological regulation	0.169451082	0.975	0.728374061
biological_process	GO:0007165	signal transduction	0.182041445	0.986	0.728374061
biological_process	GO:0050789	regulation of biological process	0.182041445	0.986	0.728374061
biological_process	GO:0050794	regulation of cellular process	0.182041445	0.986	0.728374061
biological_process	GO:0051716	cellular response to stimulus	0.182041445	0.986	0.728374061

ontology	node_id	node_name	raw_p_overrep	FWER_overrep	qvalue
biological_process	GO:0034330	cell junction organization	0.192564658	0.994	0.728374061
biological_process	GO:0040011	locomotion	0.20200589	0.995	0.728374061
biological_process	GO:0044403	symbiont process	0.21182876	0.997	0.728374061
biological_process	GO:0044419	interspecies interaction between organisms	0.21182876	0.997	0.728374061
biological_process	GO:0051704	multi-organism process	0.21182876	0.997	0.728374061
biological_process	GO:0000902	cell morphogenesis	0.212033514	0.997	0.728374061
biological_process	GO:0032989	cellular component morphogenesis	0.212033514	0.997	0.728374061
biological_process	GO:0043170	macromolecule metabolic process	0.213182652	0.998	0.728374061
biological_process	GO:0044281	small molecule metabolic process	0.228160739	0.998	0.763640025
biological_process	GO:0006928	movement of cell or subcellular component	0.243221961	0.998	0.767084645
biological_process	GO:0048870	cell motility	0.243221961	0.998	0.767084645
biological_process	GO:0051674	localization of cell	0.243221961	0.998	0.767084645
biological_process	GO:0006913	nucleocytoplasmic transport	0.293190212	0.998	0.890429532
biological_process	GO:0051169	nuclear transport	0.293190212	0.998	0.890429532
biological_process	GO:0051179	localization	0.308825213	0.998	0.920860636
biological_process	GO:0006810	transport	0.381950437	1	1
biological_process	GO:0051234	establishment of localization	0.381950437	1	1
biological_process	GO:0016043	cellular component organization	0.390362777	1	1
biological_process	GO:0006914	autophagy	0.409745256	1	1
biological_process	GO:0061919	process utilizing autophagic mechanism	0.409745256	1	1
biological_process	GO:0048646	anatomical structure formation involved in morphogenesis	0.425276384	1	1
biological_process	GO:0007005	mitochondrion organization	0.454216394	1	1
biological_process	GO:0055085	transmembrane transport	0.457817797	1	1
biological_process	GO:0061024	membrane organization	0.492973595	1	1
biological_process	GO:0071840	cellular component organization or biogenesis	0.510470477	1	1
biological_process	GO:0007267	cell-cell signaling	0.527526553	1	1
biological_process	GO:0051186	cofactor metabolic process	0.55945645	1	1
biological_process	GO:0006464	cellular protein modification process	0.562011557	1	1
biological_process	GO:0036211	protein modification process	0.562011557	1	1
biological_process	GO:0043412	macromolecule modification	0.562011557	1	1
biological_process	GO:0044237	cellular metabolic process	0.569729655	1	1
biological_process	GO:0046907	intracellular transport	0.59201324	1	1
biological_process	GO:0051641	cellular localization	0.59201324	1	1
biological_process	GO:0051649	establishment of localization in cell	0.59201324	1	1
biological_process	GO:0022607	cellular component assembly	0.601595032	1	1
biological_process	GO:0007049	cell cycle	0.65957998	1	1
biological_process	GO:0006996	organelle organization	0.677643051	1	1
biological_process	GO:0008283	cell proliferation	0.686775765	1	1
biological_process	GO:0044248	cellular catabolic process	0.694533821	1	1
biological_process	GO:0034641	cellular nitrogen compound metabolic process	0.695739754	1	1
biological_process	GO:0006082	organic acid metabolic process	0.699099749	1	1
biological_process	GO:0006520	cellular amino acid metabolic process	0.699099749	1	1
biological_process	GO:0019752	carboxylic acid metabolic process	0.699099749	1	1

ontology	node_id	node_name	raw_p_overrep	FWER_overrep	qvalue
biological_process	GO:0043436	oxoacid metabolic process	0.699099749	1	1
biological_process	GO:0006412	translation	0.723064429	1	1
biological_process	GO:0006518	peptide metabolic process	0.723064429	1	1
biological_process	GO:0009059	macromolecule biosynthetic process	0.723064429	1	1
biological_process	GO:0034645	cellular macromolecule biosynthetic process	0.723064429	1	1
biological_process	GO:0043043	peptide biosynthetic process	0.723064429	1	1
biological_process	GO:0043603	cellular amide metabolic process	0.723064429	1	1
biological_process	GO:0043604	amide biosynthetic process	0.723064429	1	1
biological_process	GO:0044249	cellular biosynthetic process	0.723064429	1	1
biological_process	GO:0044271	cellular nitrogen compound biosynthetic process	0.723064429	1	1
biological_process	GO:1901566	organonitrogen compound biosynthetic process	0.723064429	1	1
biological_process	GO:1901576	organic substance biosynthetic process	0.723064429	1	1
biological_process	GO:0008219	cell death	0.726531627	1	1
biological_process	GO:0044085	cellular component biogenesis	0.747897987	1	1
biological_process	GO:0044267	cellular protein metabolic process	0.767112945	1	1
biological_process	GO:0050877	nervous system process	0.770324023	1	1
biological_process	GO:0007010	cytoskeleton organization	0.782810667	1	1
biological_process	GO:0019538	protein metabolic process	0.788793063	1	1
biological_process	GO:0006807	nitrogen compound metabolic process	0.799448347	1	1
biological_process	GO:0032501	multicellular organismal process	0.80046482	1	1
biological_process	GO:0003008	system process	0.800835345	1	1
biological_process	GO:0043933	protein-containing complex subunit organization	0.801849627	1	1
biological_process	GO:0065003	protein-containing complex assembly	0.801849627	1	1
biological_process	GO:0009058	biosynthetic process	0.802577982	1	1
biological_process	GO:0007275	multicellular organism development	0.805360706	1	1
biological_process	GO:0009790	embryo development	0.805360706	1	1
biological_process	GO:0051276	chromosome organization	0.815066347	1	1
biological_process	GO:0016192	vesicle-mediated transport	0.835657729	1	1
biological_process	GO:1901564	organonitrogen compound metabolic process	0.844964072	1	1
biological_process	GO:0010467	gene expression	0.897551836	1	1
biological_process	GO:0008150	biological_process	1	1	1
biological_process	GO:0071941	nitrogen cycle metabolic process	1	1	1
biological_process	GO:0032196	transposition	1	1	1
biological_process	GO:0007034	vacuolar transport	1	1	1
biological_process	GO:0030198	extracellular matrix organization	1	1	1
biological_process	GO:0043062	extracellular structure organization	1	1	1
biological_process	GO:0003013	circulatory system process	1	1	1
biological_process	GO:0019748	secondary metabolic process	1	1	1
biological_process	GO:0030705	cytoskeleton-dependent intracellular transport	1	1	1
biological_process	GO:0051604	protein maturation	1	1	1
biological_process	GO:0043473	pigmentation	1	1	1
biological_process	GO:0006457	protein folding	1	1	1
biological_process	GO:0006605	protein targeting	1	1	1

ontology	node_id	node_name	raw_p_overrep	FWER_overrep	qvalue
biological_process	GO:0006886	intracellular protein transport	1	1	1
biological_process	GO:0008104	protein localization	1	1	1
biological_process	GO:0015031	protein transport	1	1	1
biological_process	GO:0015833	peptide transport	1	1	1
biological_process	GO:0033036	macromolecule localization	1	1	1
biological_process	GO:0034613	cellular protein localization	1	1	1
biological_process	GO:0042886	amide transport	1	1	1
biological_process	GO:0045184	establishment of protein localization	1	1	1
biological_process	GO:0070727	cellular macromolecule localization	1	1	1
biological_process	GO:0071702	organic substance transport	1	1	1
biological_process	GO:0071705	nitrogen compound transport	1	1	1
biological_process	GO:0022618	ribonucleoprotein complex assembly	1	1	1
biological_process	GO:0034622	cellular protein-containing complex assembly	1	1	1
biological_process	GO:0071826	ribonucleoprotein complex subunit organization	1	1	1
biological_process	GO:0006790	sulfur compound metabolic process	1	1	1
biological_process	GO:0006399	tRNA metabolic process	1	1	1
biological_process	GO:0034660	ncRNA metabolic process	1	1	1
biological_process	GO:0000280	nuclear division	1	1	1
biological_process	GO:0022402	cell cycle process	1	1	1
biological_process	GO:0048285	organelle fission	1	1	1
biological_process	GO:0140014	mitotic nuclear division	1	1	1
biological_process	GO:1903047	mitotic cell cycle process	1	1	1
biological_process	GO:0007059	chromosome segregation	1	1	1
biological_process	GO:0019439	aromatic compound catabolic process	1	1	1
biological_process	GO:0034655	nucleobase-containing compound catabolic process	1	1	1
biological_process	GO:0044270	cellular nitrogen compound catabolic process	1	1	1
biological_process	GO:0046700	heterocycle catabolic process	1	1	1
biological_process	GO:1901361	organic cyclic compound catabolic process	1	1	1
biological_process	GO:1901575	organic substance catabolic process	1	1	1
biological_process	GO:0006396	RNA processing	1	1	1
biological_process	GO:0006397	mRNA processing	1	1	1
biological_process	GO:0016071	mRNA metabolic process	1	1	1
biological_process	GO:0051301	cell division	1	1	1
biological_process	GO:0042254	ribosome biogenesis	1	1	1
biological_process	GO:0022613	ribonucleoprotein complex biogenesis	1	1	1
biological_process	GO:0016070	RNA metabolic process	1	1	1
biological_process	GO:0040007	growth	1	1	1
biological_process	GO:0000278	mitotic cell cycle	1	1	1

Table SI 12

Gene family loss in Demodex

ontology	node_id	node_name	raw_p_overrep	FWER_overrep	qvalue
biological_process	GO:0071840	**cellular component organization or biogenesis**	1.06E-05	0	0.001741165
biological_process	GO:0009987	**cellular process**	0.001053662	0.042	0.064736089
biological_process	GO:0022613	**ribonucleoprotein complex biogenesis**	0.001349684	0.048	0.064736089
biological_process	GO:0042254	**ribosome biogenesis**	0.001578929	0.06	0.064736089
biological_process	GO:0044085	**cellular component biogenesis**	0.00245798	0.095	0.080621753
biological_process	GO:0016043	**cellular component organization**	0.003418112	0.13	0.083263927
biological_process	GO:0000902	**cell morphogenesis**	0.00604121	0.221	0.083263927
biological_process	GO:0032989	**cellular component morphogenesis**	0.00604121	0.221	0.083263927
biological_process	GO:0006412	**translation**	0.009646431	0.335	0.083263927
biological_process	GO:0006518	**peptide metabolic process**	0.009646431	0.335	0.083263927
biological_process	GO:0009059	**macromolecule biosynthetic process**	0.009646431	0.335	0.083263927
biological_process	GO:0034645	**cellular macromolecule biosynthetic process**	0.009646431	0.335	0.083263927
biological_process	GO:0043043	**peptide biosynthetic process**	0.009646431	0.335	0.083263927
biological_process	GO:0043603	**cellular amide metabolic process**	0.009646431	0.335	0.083263927
biological_process	GO:0043604	**amide biosynthetic process**	0.009646431	0.335	0.083263927
biological_process	GO:0044249	**cellular biosynthetic process**	0.009646431	0.335	0.083263927
biological_process	GO:0044271	**cellular nitrogen compound biosynthetic process**	0.009646431	0.335	0.083263927
biological_process	GO:1901566	**organonitrogen compound biosynthetic process**	0.009646431	0.335	0.083263927
biological_process	GO:1901576	**organic substance biosynthetic process**	0.009646431	0.335	0.083263927
biological_process	GO:0009058	biosynthetic process	0.013939565	0.425	0.114304434
biological_process	GO:0010467	gene expression	0.014676231	0.456	0.114614376
biological_process	GO:0008152	metabolic process	0.024770389	0.609	0.179443035
biological_process	GO:0006790	sulfur compound metabolic process	0.025165791	0.62	0.179443035
biological_process	GO:1901564	organonitrogen compound metabolic process	0.043934443	0.771	0.300218694
biological_process	GO:0044281	small molecule metabolic process	0.053966828	0.816	0.354022393
biological_process	GO:0006810	transport	0.066392058	0.874	0.396157912
biological_process	GO:0051234	establishment of localization	0.066392058	0.874	0.396157912
biological_process	GO:0051186	cofactor metabolic process	0.069859781	0.891	0.396157912
biological_process	GO:0051179	localization	0.072176341	0.902	0.396157912
biological_process	GO:0044238	primary metabolic process	0.074883508	0.911	0.396157912
biological_process	GO:0071704	organic substance metabolic process	0.074883508	0.911	0.396157912
biological_process	GO:0044267	cellular protein metabolic process	0.095434595	0.952	0.4891023
biological_process	GO:0040011	locomotion	0.103200534	0.965	0.512875382
biological_process	GO:0009653	anatomical structure morphogenesis	0.12812015	0.984	0.574452123
biological_process	GO:0007267	cell-cell signaling	0.131590292	0.984	0.574452123
biological_process	GO:0019538	protein metabolic process	0.135767357	0.985	0.574452123
biological_process	GO:0006629	lipid metabolic process	0.152574468	0.992	0.574452123
biological_process	GO:0022607	cellular component assembly	0.159648252	0.992	0.574452123
biological_process	GO:0048869	cellular developmental process	0.168998263	0.993	0.574452123
biological_process	GO:0003013	circulatory system process	0.17122575	0.995	0.574452123

ontology	node_id	node_name	raw_p_overrep	FWER_overrep	qvalue
biological_process	GO:0005975	carbohydrate metabolic process	0.178265402	0.995	0.574452123
biological_process	GO:0030154	cell differentiation	0.180783508	0.995	0.574452123
biological_process	GO:0007010	cytoskeleton organization	0.190082851	0.995	0.574452123
biological_process	GO:0044237	cellular metabolic process	0.19362453	0.995	0.574452123
biological_process	GO:0006996	organelle organization	0.198360394	0.998	0.574452123
biological_process	GO:0034330	cell junction organization	0.201938732	0.999	0.574452123
biological_process	GO:0006807	nitrogen compound metabolic process	0.203797937	0.999	0.574452123
biological_process	GO:0006457	protein folding	0.212263833	0.999	0.574452123
biological_process	GO:0055085	transmembrane transport	0.224216734	0.999	0.574452123
biological_process	GO:0006091	generation of precursor metabolites and energy	0.228412211	0.999	0.574452123
biological_process	GO:0006082	organic acid metabolic process	0.234093385	0.999	0.574452123
biological_process	GO:0006520	cellular amino acid metabolic process	0.234093385	0.999	0.574452123
biological_process	GO:0019752	carboxylic acid metabolic process	0.234093385	0.999	0.574452123
biological_process	GO:0043436	oxoacid metabolic process	0.234093385	0.999	0.574452123
biological_process	GO:0007155	cell adhesion	0.234479675	0.999	0.574452123
biological_process	GO:0022610	biological adhesion	0.234479675	0.999	0.574452123
biological_process	GO:0006605	protein targeting	0.238187465	0.999	0.574452123
biological_process	GO:0006886	intracellular protein transport	0.238187465	0.999	0.574452123
biological_process	GO:0008104	protein localization	0.238187465	0.999	0.574452123
biological_process	GO:0015031	protein transport	0.238187465	0.999	0.574452123
biological_process	GO:0015833	peptide transport	0.238187465	0.999	0.574452123
biological_process	GO:0033036	macromolecule localization	0.238187465	0.999	0.574452123
biological_process	GO:0034613	cellular protein localization	0.238187465	0.999	0.574452123
biological_process	GO:0042886	amide transport	0.238187465	0.999	0.574452123
biological_process	GO:0045184	establishment of protein localization	0.238187465	0.999	0.574452123
biological_process	GO:0070727	cellular macromolecule localization	0.238187465	0.999	0.574452123
biological_process	GO:0071702	organic substance transport	0.238187465	0.999	0.574452123
biological_process	GO:0071705	nitrogen compound transport	0.238187465	0.999	0.574452123
biological_process	GO:0032502	developmental process	0.25451468	1	0.604933443
biological_process	GO:0048856	anatomical structure development	0.276000911	1	0.646630705
biological_process	GO:0034641	cellular nitrogen compound metabolic process	0.299472113	1	0.691738401
biological_process	GO:0040007	growth	0.308640182	1	0.703013748
biological_process	GO:0044260	cellular macromolecule metabolic process	0.315591782	1	0.709000715
biological_process	GO:0043170	macromolecule metabolic process	0.348286904	1	0.733597838
biological_process	GO:0043933	protein-containing complex subunit organization	0.352569703	1	0.733597838
biological_process	GO:0065003	protein-containing complex assembly	0.352569703	1	0.733597838
biological_process	GO:0006396	RNA processing	0.359854261	1	0.733597838
biological_process	GO:0006397	mRNA processing	0.359854261	1	0.733597838
biological_process	GO:0016071	mRNA metabolic process	0.359854261	1	0.733597838
biological_process	GO:0061024	membrane organization	0.361092246	1	0.733597838
biological_process	GO:0007568	aging	0.363030875	1	0.733597838
biological_process	GO:0071554	cell wall organization or biogenesis	0.366798919	1	0.733597838
biological_process	GO:0016192	vesicle-mediated transport	0.388735272	1	0.746392249
biological_process	GO:0007154	cell communication	0.39152288	1	0.746392249

ontology	node_id	node_name	raw_p_overrep	FWER_overrep	qvalue
biological_process	GO:0023052	signaling	0.39152288	1	0.746392249
biological_process	GO:0006464	cellular protein modification process	0.400503158	1	0.746392249
biological_process	GO:0036211	protein modification process	0.400503158	1	0.746392249
biological_process	GO:0043412	macromolecule modification	0.400503158	1	0.746392249
biological_process	GO:0042592	homeostatic process	0.428903071	1	0.777220411
biological_process	GO:0065008	regulation of biological quality	0.428903071	1	0.777220411
biological_process	GO:0030705	cytoskeleton-dependent intracellular transport	0.431262545	1	0.777220411
biological_process	GO:0007275	multicellular organism development	0.485004615	1	0.851787083
biological_process	GO:0009790	embryo development	0.485004615	1	0.851787083
biological_process	GO:0051604	protein maturation	0.511338541	1	0.851787083
biological_process	GO:0022618	ribonucleoprotein complex assembly	0.512766771	1	0.851787083
biological_process	GO:0034622	cellular protein-containing complex assembly	0.512766771	1	0.851787083
biological_process	GO:0071826	ribonucleoprotein complex subunit organization	0.512766771	1	0.851787083
biological_process	GO:0051276	chromosome organization	0.527423946	1	0.851787083
biological_process	GO:0006914	autophagy	0.528298514	1	0.851787083
biological_process	GO:0061919	process utilizing autophagic mechanism	0.528298514	1	0.851787083
biological_process	GO:0006928	movement of cell or subcellular component	0.534963839	1	0.851787083
biological_process	GO:0048870	cell motility	0.534963839	1	0.851787083
biological_process	GO:0051674	localization of cell	0.534963839	1	0.851787083
biological_process	GO:0051301	cell division	0.556106096	1	0.876936537
biological_process	GO:0006950	response to stress	0.616557136	1	0.924210792
biological_process	GO:0065007	biological regulation	0.619234404	1	0.924210792
biological_process	GO:0007034	vacuolar transport	0.620165163	1	0.924210792
biological_process	GO:0046907	intracellular transport	0.622360589	1	0.924210792
biological_process	GO:0051641	cellular localization	0.622360589	1	0.924210792
biological_process	GO:0051649	establishment of localization in cell	0.622360589	1	0.924210792
biological_process	GO:0016070	RNA metabolic process	0.633448753	1	0.924210792
biological_process	GO:0021700	developmental maturation	0.633665138	1	0.924210792
biological_process	GO:0007005	mitochondrion organization	0.636803778	1	0.924210792
biological_process	GO:0050896	response to stimulus	0.661702481	1	0.947510784
biological_process	GO:0043473	pigmentation	0.668893693	1	0.947510784
biological_process	GO:0030198	extracellular matrix organization	0.67596806	1	0.947510784
biological_process	GO:0043062	extracellular structure organization	0.67596806	1	0.947510784
biological_process	GO:0007165	signal transduction	0.715783728	1	0.970153151
biological_process	GO:0050789	regulation of biological process	0.715783728	1	0.970153151
biological_process	GO:0050794	regulation of cellular process	0.715783728	1	0.970153151
biological_process	GO:0051716	cellular response to stimulus	0.715783728	1	0.970153151
biological_process	GO:0006259	DNA metabolic process	0.803769892	1	1
biological_process	GO:0090304	nucleic acid metabolic process	0.815500516	1	1
biological_process	GO:0008219	cell death	0.81918556	1	1
biological_process	GO:0044403	symbiont process	0.825557148	1	1
biological_process	GO:0044419	interspecies interaction between organisms	0.825557148	1	1
biological_process	GO:0051704	multi-organism process	0.825557148	1	1
biological_process	GO:0000003	reproduction	0.847166808	1	1

ontology	node_id	node_name	raw_p_overrep	FWER_overrep	qvalue
biological_process	GO:0032501	multicellular organismal process	0.85244846	1	1
biological_process	GO:0002376	immune system process	0.863456662	1	1
biological_process	GO:0048646	anatomical structure formation involved in morphogenesis	0.904945927	1	1
biological_process	GO:0007059	chromosome segregation	0.912899052	1	1
biological_process	GO:0009056	catabolic process	0.928837298	1	1
biological_process	GO:0006139	nucleobase-containing compound metabolic process	0.946192676	1	1
biological_process	GO:0006725	cellular aromatic compound metabolic process	0.946192676	1	1
biological_process	GO:0046483	heterocycle metabolic process	0.946192676	1	1
biological_process	GO:1901360	organic cyclic compound metabolic process	0.946192676	1	1
biological_process	GO:0006399	tRNA metabolic process	0.949763075	1	1
biological_process	GO:0034660	ncRNA metabolic process	0.949763075	1	1
biological_process	GO:0007049	cell cycle	0.964901836	1	1
biological_process	GO:0044248	cellular catabolic process	0.971927658	1	1
biological_process	GO:0003008	system process	0.974387194	1	1
biological_process	GO:0008283	cell proliferation	0.990351413	1	1
biological_process	GO:0000278	mitotic cell cycle	0.996615052	1	1
biological_process	GO:0050877	nervous system process	0.998464955	1	1
biological_process	GO:0008150	biological_process	1	1	1
biological_process	GO:0071941	nitrogen cycle metabolic process	1	1	1
biological_process	GO:0032196	transposition	1	1	1
biological_process	GO:0007009	plasma membrane organization	1	1	1
biological_process	GO:0010256	endomembrane system organization	1	1	1
biological_process	GO:0019748	secondary metabolic process	1	1	1
biological_process	GO:0006913	nucleocytoplasmic transport	1	1	1
biological_process	GO:0051169	nuclear transport	1	1	1
biological_process	GO:0000280	nuclear division	1	1	1
biological_process	GO:0022402	cell cycle process	1	1	1
biological_process	GO:0048285	organelle fission	1	1	1
biological_process	GO:0140014	mitotic nuclear division	1	1	1
biological_process	GO:1903047	mitotic cell cycle process	1	1	1
biological_process	GO:0019439	aromatic compound catabolic process	1	1	1
biological_process	GO:0034655	nucleobase-containing compound catabolic process	1	1	1
biological_process	GO:0044270	cellular nitrogen compound catabolic process	1	1	1
biological_process	GO:0046700	heterocycle catabolic process	1	1	1
biological_process	GO:1901361	organic cyclic compound catabolic process	1	1	1
biological_process	GO:1901575	organic substance catabolic process	1	1	1

Table SI 13

Gene family loss in the Acariformes

ontology	node_id	node_name	raw_p_overrep	FWER_overrep	qvalue
biological_process	GO:0071941	nitrogen cycle metabolic process	0.004596994	0.19	0.38951373
biological_process	GO:0006259	DNA metabolic process	0.011963106	0.411	0.38951373
biological_process	GO:0044260	cellular macromolecule metabolic process	0.012425921	0.42	0.38951373
biological_process	GO:0006464	cellular protein modification process	0.014250502	0.462	0.38951373
biological_process	GO:0036211	protein modification process	0.014250502	0.462	0.38951373
biological_process	GO:0043412	macromolecule modification	0.014250502	0.462	0.38951373
biological_process	GO:0051276	chromosome organization	0.020695666	0.562	0.4848699
biological_process	GO:0009987	cellular process	0.026778698	0.643	0.52586716
biological_process	GO:0043170	macromolecule metabolic process	0.028858564	0.671	0.52586716
biological_process	GO:0044238	primary metabolic process	0.061039877	0.879	0.91004907
biological_process	GO:0071704	organic substance metabolic process	0.061039877	0.879	0.91004907
biological_process	GO:0019538	protein metabolic process	0.082110029	0.943	0.94424454
biological_process	GO:1901564	organonitrogen compound metabolic process	0.099534568	0.963	0.94424454
biological_process	GO:0044267	cellular protein metabolic process	0.105159341	0.975	0.94424454
biological_process	GO:0000280	nuclear division	0.114319842	0.979	0.94424454
biological_process	GO:0022402	cell cycle process	0.114319842	0.979	0.94424454
biological_process	GO:0048285	organelle fission	0.114319842	0.979	0.94424454
biological_process	GO:0140014	mitotic nuclear division	0.114319842	0.979	0.94424454
biological_process	GO:1903047	mitotic cell cycle process	0.114319842	0.979	0.94424454
biological_process	GO:0006807	nitrogen compound metabolic process	0.119146254	0.987	0.94424454
biological_process	GO:0007059	chromosome segregation	0.120909362	0.988	0.94424454
biological_process	GO:0008152	metabolic process	0.183138471	0.997	1
biological_process	GO:0044237	cellular metabolic process	0.270845193	1	1
biological_process	GO:0009056	catabolic process	0.291524051	1	1
biological_process	GO:0090304	nucleic acid metabolic process	0.304837486	1	1
biological_process	GO:0022618	ribonucleoprotein complex assembly	0.308203039	1	1
biological_process	GO:0034622	cellular protein-containing complex assembly	0.308203039	1	1
biological_process	GO:0071826	ribonucleoprotein complex subunit organization	0.308203039	1	1
biological_process	GO:0006996	organelle organization	0.323962456	1	1
biological_process	GO:0006139	nucleobase-containing compound metabolic process	0.32488387	1	1
biological_process	GO:0006725	cellular aromatic compound metabolic process	0.32488387	1	1
biological_process	GO:0046483	heterocycle metabolic process	0.32488387	1	1
biological_process	GO:1901360	organic cyclic compound metabolic process	0.32488387	1	1
biological_process	GO:0016043	cellular component organization	0.330666898	1	1
biological_process	GO:0006790	sulfur compound metabolic process	0.344946803	1	1
biological_process	GO:0050896	response to stimulus	0.375493445	1	1
biological_process	GO:0000278	mitotic cell cycle	0.390477332	1	1
biological_process	GO:0007049	cell cycle	0.390640683	1	1
biological_process	GO:0071840	cellular component organization or biogenesis	0.391468641	1	1
biological_process	GO:0007009	plasma membrane organization	0.420109841	1	1

ontology	node_id	node_name	raw_p_overrep	FWER_overrep	qvalue
biological_process	GO:0010256	endomembrane system organization	0.420109841	1	1
biological_process	GO:0007155	cell adhesion	0.426379461	1	1
biological_process	GO:0022610	biological adhesion	0.426379461	1	1
biological_process	GO:0006457	protein folding	0.443671225	1	1
biological_process	GO:0034330	cell junction organization	0.477563339	1	1
biological_process	GO:0051604	protein maturation	0.528783914	1	1
biological_process	GO:0019439	aromatic compound catabolic process	0.543377088	1	1
biological_process	GO:0034655	nucleobase-containing compound catabolic process	0.543377088	1	1
biological_process	GO:0044270	cellular nitrogen compound catabolic process	0.543377088	1	1
biological_process	GO:0046700	heterocycle catabolic process	0.543377088	1	1
biological_process	GO:1901361	organic cyclic compound catabolic process	0.543377088	1	1
biological_process	GO:1901575	organic substance catabolic process	0.543377088	1	1
biological_process	GO:0000902	cell morphogenesis	0.544063053	1	1
biological_process	GO:0032989	cellular component morphogenesis	0.544063053	1	1
biological_process	GO:0016192	vesicle-mediated transport	0.551638793	1	1
biological_process	GO:0065007	biological regulation	0.570837378	1	1
biological_process	GO:0002376	immune system process	0.58218119	1	1
biological_process	GO:0006950	response to stress	0.600011394	1	1
biological_process	GO:0022613	ribonucleoprotein complex biogenesis	0.611317973	1	1
biological_process	GO:0008283	cell proliferation	0.623329303	1	1
biological_process	GO:0007034	vacuolar transport	0.632202729	1	1
biological_process	GO:0048646	anatomical structure formation involved in morphogenesis	0.645575671	1	1
biological_process	GO:0044248	cellular catabolic process	0.646753605	1	1
biological_process	GO:0007165	signal transduction	0.654188707	1	1
biological_process	GO:0050789	regulation of biological process	0.654188707	1	1
biological_process	GO:0050794	regulation of cellular process	0.654188707	1	1
biological_process	GO:0051716	cellular response to stimulus	0.654188707	1	1
biological_process	GO:0009653	anatomical structure morphogenesis	0.673885968	1	1
biological_process	GO:0007154	cell communication	0.691186605	1	1
biological_process	GO:0023052	signaling	0.691186605	1	1
biological_process	GO:0051186	cofactor metabolic process	0.729432783	1	1
biological_process	GO:0006913	nucleocytoplasmic transport	0.735289987	1	1
biological_process	GO:0051169	nuclear transport	0.735289987	1	1
biological_process	GO:0030154	cell differentiation	0.735695164	1	1
biological_process	GO:0008219	cell death	0.739428246	1	1
biological_process	GO:0043933	protein-containing complex subunit organization	0.747869356	1	1
biological_process	GO:0065003	protein-containing complex assembly	0.747869356	1	1
biological_process	GO:0006914	autophagy	0.755431047	1	1
biological_process	GO:0061919	process utilizing autophagic mechanism	0.755431047	1	1
biological_process	GO:0003013	circulatory system process	0.775346593	1	1
biological_process	GO:0048869	cellular developmental process	0.778557326	1	1
biological_process	GO:0042254	ribosome biogenesis	0.782503945	1	1
biological_process	GO:0007010	cytoskeleton organization	0.789624631	1	1

ontology	node_id	node_name	raw_p_overrep	FWER_overrep	qvalue
biological_process	GO:0006399	tRNA metabolic process	0.811500514	1	1
biological_process	GO:0034660	ncRNA metabolic process	0.811500514	1	1
biological_process	GO:0042592	homeostatic process	0.825763053	1	1
biological_process	GO:0065008	regulation of biological quality	0.825763053	1	1
biological_process	GO:0007568	aging	0.826622932	1	1
biological_process	GO:0021700	developmental maturation	0.829596269	1	1
biological_process	GO:0007005	mitochondrion organization	0.831632086	1	1
biological_process	GO:0040007	growth	0.840847148	1	1
biological_process	GO:0034641	cellular nitrogen compound metabolic process	0.855455086	1	1
biological_process	GO:0061024	membrane organization	0.883703358	1	1
biological_process	GO:0022607	cellular component assembly	0.890133706	1	1
biological_process	GO:0032502	developmental process	0.898877734	1	1
biological_process	GO:0006810	transport	0.905196188	1	1
biological_process	GO:0051234	establishment of localization	0.905196188	1	1
biological_process	GO:0003008	system process	0.90769941	1	1
biological_process	GO:0048856	anatomical structure development	0.913257154	1	1
biological_process	GO:0050877	nervous system process	0.917869581	1	1
biological_process	GO:0005975	carbohydrate metabolic process	0.921327947	1	1
biological_process	GO:0044085	cellular component biogenesis	0.921764357	1	1
biological_process	GO:0000003	reproduction	0.922863826	1	1
biological_process	GO:0009058	biosynthetic process	0.925139377	1	1
biological_process	GO:0006629	lipid metabolic process	0.949040387	1	1
biological_process	GO:0040011	locomotion	0.953150599	1	1
biological_process	GO:0007267	cell-cell signaling	0.955444497	1	1
biological_process	GO:0007275	multicellular organism development	0.957872507	1	1
biological_process	GO:0009790	embryo development	0.957872507	1	1
biological_process	GO:0006412	translation	0.962000821	1	1
biological_process	GO:0006518	peptide metabolic process	0.962000821	1	1
biological_process	GO:0009059	macromolecule biosynthetic process	0.962000821	1	1
biological_process	GO:0034645	cellular macromolecule biosynthetic process	0.962000821	1	1
biological_process	GO:0043043	peptide biosynthetic process	0.962000821	1	1
biological_process	GO:0043603	cellular amide metabolic process	0.962000821	1	1
biological_process	GO:0043604	amide biosynthetic process	0.962000821	1	1
biological_process	GO:0044249	cellular biosynthetic process	0.962000821	1	1
biological_process	GO:0044271	cellular nitrogen compound biosynthetic process	0.962000821	1	1
biological_process	GO:1901566	organonitrogen compound biosynthetic process	0.962000821	1	1
biological_process	GO:1901576	organic substance biosynthetic process	0.962000821	1	1
biological_process	GO:0051179	localization	0.966860941	1	1
biological_process	GO:0044281	small molecule metabolic process	0.970255898	1	1
biological_process	GO:0032501	multicellular organismal process	0.975433312	1	1
biological_process	GO:0006082	organic acid metabolic process	0.978816461	1	1
biological_process	GO:0006520	cellular amino acid metabolic process	0.978816461	1	1
biological_process	GO:0019752	carboxylic acid metabolic process	0.978816461	1	1
biological_process	GO:0043436	oxoacid metabolic process	0.978816461	1	1

ontology	node_id	node_name	raw_p_overrep	FWER_overrep	qvalue
biological_process	GO:0051301	cell division	0.986811841	1	1
biological_process	GO:0046907	intracellular transport	0.991070368	1	1
biological_process	GO:0051641	cellular localization	0.991070368	1	1
biological_process	GO:0051649	establishment of localization in cell	0.991070368	1	1
biological_process	GO:0010467	gene expression	0.994152793	1	1
biological_process	GO:0006928	movement of cell or subcellular component	0.994270882	1	1
biological_process	GO:0048870	cell motility	0.994270882	1	1
biological_process	GO:0051674	localization of cell	0.994270882	1	1
biological_process	GO:0016070	RNA metabolic process	0.994305797	1	1
biological_process	GO:0055085	transmembrane transport	0.99744064	1	1
biological_process	GO:0006396	RNA processing	0.997842852	1	1
biological_process	GO:0006397	mRNA processing	0.997842852	1	1
biological_process	GO:0016071	mRNA metabolic process	0.997842852	1	1
biological_process	GO:0008150	biological_process	1	1	1
biological_process	GO:0032196	transposition	1	1	1
biological_process	GO:0071554	cell wall organization or biogenesis	1	1	1
biological_process	GO:0030198	extracellular matrix organization	1	1	1
biological_process	GO:0043062	extracellular structure organization	1	1	1
biological_process	GO:0019748	secondary metabolic process	1	1	1
biological_process	GO:0030705	cytoskeleton-dependent intracellular transport	1	1	1
biological_process	GO:0044403	symbiont process	1	1	1
biological_process	GO:0044419	interspecies interaction between organisms	1	1	1
biological_process	GO:0051704	multi-organism process	1	1	1
biological_process	GO:0043473	pigmentation	1	1	1
biological_process	GO:0006605	protein targeting	1	1	1
biological_process	GO:0006886	intracellular protein transport	1	1	1
biological_process	GO:0008104	protein localization	1	1	1
biological_process	GO:0015031	protein transport	1	1	1
biological_process	GO:0015833	peptide transport	1	1	1
biological_process	GO:0033036	macromolecule localization	1	1	1
biological_process	GO:0034613	cellular protein localization	1	1	1
biological_process	GO:0042886	amide transport	1	1	1
biological_process	GO:0045184	establishment of protein localization	1	1	1
biological_process	GO:0070727	cellular macromolecule localization	1	1	1
biological_process	GO:0071702	organic substance transport	1	1	1
biological_process	GO:0071705	nitrogen compound transport	1	1	1
biological_process	GO:0006091	generation of precursor metabolites and energy	1	1	1

Table SI 14

RELAX test significant orthogroups

Orthogroup	No. codons	omega spiders/scorpions	omega Acariformes	RELAX null log-likelihood	RELAX alternative log-likelihood	RELAX K parameter	*p*-value	SGoF adjusted *p*-value
OG0002034	428	0.017	0.1167	-13259.34	-13249.87	0.2	0	0.0016
OG0002407	218	0.0119	0.015	-4187.74	-4161.53	7.06	0	0.0016
OG0003444	788	0.0052	0.0085	-17427.94	-17419.58	0.61	0	0.0016
OG0004159	314	0.0217	0.0095	-7561.59	-7552.82	1.06	0	0.0016
OG0004254	309	0.0098	0.0539	-6969.44	-6958	0.18	0	0.0016
OG0004266	327	0.0478	0.0565	-8694.06	-8572.65	0.83	0	0.0016
OG0004288	430	0.0156	0.0136	-11033.26	-11003.13	1.04	0	0.0016
OG0004689	1081	0.0076	0.0264	-24156.75	-24139.02	0.23	0	0.0016
OG0004728	321	0.0376	0.0319	-10692.35	-10594.26	0.8	0	0.0016
OG0005109	377	0.0114	0.0363	-15384.38	-15147.02	0.94	0	0.0016
OG0005307	424	0.034	0.0499	-12364.88	-12345.49	0.89	0	0.0016
OG0005333	150	0.0519	0.0095	-4480.29	-4466	0.95	0	0.0016
OG0005379	2046	0.0029	0.0133	-43386.9	-43362.39	0.12	0	0.0016
OG0005399	1044	0.0065	0.0348	-24099.55	-24072.93	0.59	0	0.0016
OG0005576	215	0.029	0.0378	-5198.53	-5185.13	1.3	0	0.0016
OG0004832	286	4.00E-04	0.0163	-6682.25	-6674.83	0.28	1.00E-04	0.0022
OG0004978	351	0.0153	0.0332	-9854.15	-9846.65	0.19	1.00E-04	0.0022
OG0005329	277	0.0081	0.0449	-6422.48	-6414.41	0.33	1.00E-04	0.0022
OG0003260	167	0.0171	0.0631	-6243.69	-6236.97	0.98	2.00E-04	0.0027
OG0005149	142	0.0018	0.0399	-3207.27	-3200.3	0.11	2.00E-04	0.0027
OG0005207	453	0.0036	0.0367	-13138.53	-13132.09	0.26	3.00E-04	0.0029
OG0004223	567	0.0572	0.0851	-17248.81	-17242.59	0.57	4.00E-04	0.003
OG0005045	188	0.0221	0.0989	-6137.99	-6132.18	0.37	6.00E-04	0.0038
OG0005391	371	0.0113	0.0463	-9145.95	-9140.01	0.17	6.00E-04	0.0038
OG0005680	88	0.0546	0.1453	-3014.85	-3009.11	0.27	7.00E-04	0.0041

Orthogroup	No. codons	omega spiders/scorpions	omega Acariformes	RELAX null log-likelihood	RELAX alternative log-likelihood	RELAX K parameter	*p*-value	SGoF adjusted *p*-value
OG0002809	149	0	0.0366	-2594.95	-2589.38	0.3	8.00E-04	0.0046
OG0004780	100	0.0925	0.0369	-3582.18	-3576.68	7.82	9.00E-04	0.0046
OG0004758	330	0.0271	0.0695	-10269.7	-10264.39	0.42	0.0011	0.0054
OG0006117	163	0.044	0.109	-5221.8	-5216.49	0.35	0.0011	0.0054
OG0005638	350	0.0402	0.0956	-12133.98	-12128.93	0.51	0.0015	0.006
OG0004759	259	0.0396	0.1112	-8633.59	-8628.62	0.28	0.0016	0.0061
OG0003950	61	0.0153	0.0751	-1353.06	-1348.12	0	0.0017	0.0062
OG0004588	469	0.0436	0.0713	-15374.31	-15369.55	0.49	0.002	0.007
OG0005213	561	0.0107	0.0281	-14909.26	-14904.47	0.53	0.002	0.007
OG0004162	173	0.041	0.0341	-5110.09	-5105.41	0.87	0.0022	0.008
OG0004440	412	0.0638	0.0198	-14186.21	-14181.71	1.39	0.0027	0.0093
OG0005766	134	0.0053	0.0516	-2915.81	-2911.31	0.59	0.0027	0.0093
OG0004091	192	0.3278	0.0372	-7325.68	-7321.24	1.71	0.0029	0.0096
OG0004892	224	0.0147	0.0782	-6261.06	-6256.67	0.3	0.003	0.01
OG0003839	391	0.0372	0.0207	-10347.54	-10343.18	1.56	0.0031	0.01
OG0005016	407	0.0576	0.0659	-12817.98	-12813.72	0.52	0.0035	0.0101
OG0005234	190	0.0108	0.0426	-4713.81	-4709.62	0.07	0.0038	0.0115
OG0004861	364	0.0607	0.0299	-13706.89	-13702.76	1.62	0.0041	0.0115
OG0004043	415	0.0643	0.1034	-11885.11	-11881.08	0.44	0.0046	0.0126
OG0004413	334	0.019	0.0313	-9566.34	-9562.33	0.56	0.0046	0.0126
OG0004641	233	0.0499	0.0169	-7040.75	-7036.74	1.98	0.0046	0.0126
OG0005182	271	0.0313	0.0954	-8352.15	-8348.24	0.09	0.0051	0.0137
OG0004547	172	0.016	0.029	-3908.68	-3904.81	0.9	0.0054	0.014
OG0002024	654	0.0375	0.0088	-16432.61	-16428.84	1.33	0.006	0.0169
OG0003505	467	0.0166	0.0419	-10210.75	-10206.99	0.82	0.0061	0.0193
OG0005256	1094	0.0251	0.0353	-31883	-31879.26	0.57	0.0062	0.0204
OG0004635	476	0.0542	0.0117	-16775.59	-16771.94	1.62	0.0069	0.0206
OG0004378	173	0.0256	0.1063	-4524.58	-4520.95	0	0.007	0.022

Orthogroup	No. codons	omega spiders/scorpions	omega Acariformes	RELAX null log-likelihood	RELAX alternative log-likelihood	RELAX K parameter	*p*-value	SGoF adjusted *p*-value
OG0005676	179	0.0098	0.1094	-6101.36	-6097.73	0.58	0.007	0.022
OG0005186	378	0.0495	0.044	-15037.93	-15034.41	1.45	0.008	0.0223
OG0004017	291	0.0229	0.0677	-7364.79	-7361.34	0.76	0.0086	0.0224
OG0004305	429	0.1203	0.0438	-14437.74	-14434.36	2.42	0.0093	0.0239
OG0003924	506	0.0206	0.0127	-12512.16	-12508.78	0.98	0.0094	0.026
OG0004300	145	0.0135	0.0217	-3740.42	-3737.07	0.61	0.0096	0.0265
OG0004837	313	0.0399	0.0669	-8870.3	-8866.98	0.19	0.01	0.0265
OG0005898	552	0.0138	0.0324	-13351.42	-13348.1	0.84	0.01	0.0265
OG0005894	470	0.0199	0.0275	-15476.05	-15472.74	0.26	0.0101	0.0272
OG0005904	74	0.0989	0.0082	-2537.15	-2533.92	3.2	0.011	0.0272
OG0005073	311	0.023	0.0559	-9021.01	-9017.82	0.35	0.0115	0.0367
OG0005740	275	0.0309	0.017	-8375.01	-8371.81	0.49	0.0115	0.0367
OG0004516	162	0.005	0.0309	-4862.79	-4859.65	0.62	0.0121	0.0426
OG0006208	300	0.0523	0.0107	-9970.54	-9967.39	1.99	0.0121	0.0426
OG0005140	95	0.0108	0.02	-2004.77	-2001.65	0.12	0.0126	0.0438
OG0005335	306	0.0245	0.0602	-10288.85	-10285.77	0.3	0.0131	0.0446
OG0002859	541	0.0375	0.0728	-15211.26	-15208.22	0.55	0.0137	0.0455
OG0004385	151	0.0016	0.0375	-3452.5	-3449.48	0.27	0.014	0.0458
OG0004517	206	0.0083	0.0263	-4885.33	-4882.48	0.51	0.0168	0.0458
OG0004679	254	0.0069	0.038	-6848.1	-6845.25	0.3	0.0169	0.0469
OG0003752	407	0.0146	0.0258	-11245.13	-11242.37	0.67	0.0187	0.0483
OG0004023	272	0.0524	0.0115	-8241.39	-8238.66	1.55	0.0193	0.0552
OG0004820	176	0.0043	0.0151	-4046.9	-4044.21	0.55	0.0204	0.0559
OG0004904	348	0.0281	0.0191	-12900.59	-12897.91	1.58	0.0206	0.0575
OG0004476	161	0.0071	0.0348	-4747.48	-4744.8	0.41	0.0207	0.0579
OG0003594	266	0.0663	0.0509	-8150.71	-8148.05	1.56	0.021	0.059
OG0005293	303	0.033	0.0475	-9740.18	-9737.55	0.48	0.022	0.0598
OG0003623	429	0.0359	0.0605	-12515.04	-12512.43	0.39	0.0223	0.06

Orthogroup	No. codons	omega spiders/scorpions	omega Acariformes	RELAX null log-likelihood	RELAX alternative log-likelihood	RELAX K parameter	*p*-value	SGoF adjusted *p*-value
OG0004344	251	0.0421	0.0642	-7706.79	-7704.18	0.49	0.0224	0.0624
OG0004032	166	0.0092	0.0205	-4373.66	-4371.09	0.69	0.0236	0.0644
OG0005080	222	0.0971	0.0548	-7666.12	-7663.57	1.85	0.0239	0.0644
OG0004875	203	0.0467	0.0296	-7117.15	-7114.64	1.02	0.025	0.0646
OG0003328	303	0.0065	0.0332	-8417.42	-8414.94	0.59	0.026	0.0667
OG0004207	308	0.0287	0.0554	-8614.88	-8612.42	0.43	0.0265	0.0738
OG0004610	160	0.0321	0.0148	-4714.14	-4711.68	1.67	0.0265	0.0738
OG0005091	665	0.0296	0.0478	-22186.84	-22184.38	0.67	0.0265	0.0738
OG0005220	82	0.008	0.0286	-1989.11	-1986.66	0.64	0.0271	0.074
OG0004808	202	0.0258	0.0211	-7541.21	-7538.77	0.92	0.0272	0.0767
OG0005302	162	0.0482	0.1364	-5435.11	-5432.73	0.5	0.029	0.0771
OG0004568	240	0.0519	0.0126	-8080.54	-8078.22	1.44	0.0312	0.0771
OG0004646	194	0.0316	0.0657	-5612.66	-5610.42	0.52	0.0342	0.0775
OG0003453	555	0.0226	0.0461	-16930.38	-16928.17	0.52	0.0355	0.078
OG0003205	609	0.0134	0.0319	-13237.18	-13234.98	0.73	0.0358	0.0822
OG0004080	248	0.0265	0.068	-8774.73	-8772.55	0.53	0.0367	0.083
OG0005101	231	0.0483	0.0253	-8426.44	-8424.26	1.35	0.0372	0.0848

Table SI 15

Relaxed selection enrichment

ontology	node_id	node_name	raw_p_overrep	FWER_ overrep	qvalue
biological_process	GO:0006396	**RNA processing**	0.000296311	0.008	0.009493355
biological_process	GO:0006397	**mRNA processing**	0.000296311	0.008	0.009493355
biological_process	GO:0016071	**mRNA metabolic process**	0.000296311	0.008	0.009493355
biological_process	GO:0006464	**cellular protein modification process**	0.00035379	0.012	0.009493355
biological_process	GO:0036211	**protein modification process**	0.00035379	0.012	0.009493355
biological_process	GO:0043412	**macromolecule modification**	0.00035379	0.012	0.009493355
biological_process	GO:0044238	**primary metabolic process**	0.001296222	0.058	0.024522172
biological_process	GO:0071704	**organic substance metabolic process**	0.001296222	0.058	0.024522172
biological_process	GO:0016070	**RNA metabolic process**	0.00183788	0.076	0.024522172
biological_process	GO:0006139	**nucleobase-containing compound metabolic process**	0.001980051	0.083	0.024522172
biological_process	GO:0006725	**cellular aromatic compound metabolic process**	0.001980051	0.083	0.024522172
biological_process	GO:0046483	**heterocycle metabolic process**	0.001980051	0.083	0.024522172
biological_process	GO:1901360	**organic cyclic compound metabolic process**	0.001980051	0.083	0.024522172
biological_process	GO:0043170	**macromolecule metabolic process**	0.003465608	0.143	0.039754306
biological_process	GO:0090304	**nucleic acid metabolic process**	0.003703817	0.173	0.039754306
biological_process	GO:1901564	**organonitrogen compound metabolic process**	0.007359904	0.26	0.074059033
biological_process	GO:0006082	**organic acid metabolic process**	0.010752663	0.332	0.08655894
biological_process	GO:0006520	**cellular amino acid metabolic process**	0.010752663	0.332	0.08655894
biological_process	GO:0019752	**carboxylic acid metabolic process**	0.010752663	0.332	0.08655894
biological_process	GO:0043436	**oxoacid metabolic process**	0.010752663	0.332	0.08655894
biological_process	GO:0044267	**cellular protein metabolic process**	0.012801893	0.36	0.098147845
biological_process	GO:0044237	cellular metabolic process	0.020337019	0.528	0.144624503
biological_process	GO:0006807	nitrogen compound metabolic process	0.020660643	0.529	0.144624503
biological_process	GO:0019538	protein metabolic process	0.024635519	0.555	0.16526327
biological_process	GO:0008152	metabolic process	0.040286324	0.76	0.259443924
biological_process	GO:0044260	cellular macromolecule metabolic process	0.048049138	0.807	0.297535048
biological_process	GO:0032502	developmental process	0.06846904	0.896	0.408278352
biological_process	GO:0010467	gene expression	0.083934988	0.932	0.482626179
biological_process	GO:0048856	anatomical structure development	0.094648458	0.943	0.507946725
biological_process	GO:0050896	response to stimulus	0.094648458	0.943	0.507946725
biological_process	GO:0019439	aromatic compound catabolic process	0.121575745	0.98	0.518187958
biological_process	GO:0034655	nucleobase-containing compound catabolic process	0.121575745	0.98	0.518187958
biological_process	GO:0044270	cellular nitrogen compound catabolic process	0.121575745	0.98	0.518187958
biological_process	GO:0046700	heterocycle catabolic process	0.121575745	0.98	0.518187958
biological_process	GO:1901361	organic cyclic compound catabolic process	0.121575745	0.98	0.518187958
biological_process	GO:1901575	organic substance catabolic process	0.121575745	0.98	0.518187958
biological_process	GO:0007154	cell communication	0.12264475	0.98	0.518187958
biological_process	GO:0023052	signaling	0.12264475	0.98	0.518187958
biological_process	GO:0046907	intracellular transport	0.132893482	0.986	0.518187958
biological_process	GO:0051641	cellular localization	0.132893482	0.986	0.518187958

ontology	node_id	node_name	raw_p_overrep	FWER_ overrep	qvalue
biological_process	GO:0051649	establishment of localization in cell	0.132893482	0.986	0.518187958
biological_process	GO:0003008	system process	0.138398026	0.989	0.518187958
biological_process	GO:0050877	nervous system process	0.138398026	0.989	0.518187958
biological_process	GO:0003013	circulatory system process	0.16	0.996	0.51995079
biological_process	GO:0006629	lipid metabolic process	0.168673263	0.996	0.51995079
biological_process	GO:0007165	signal transduction	0.169675612	0.996	0.51995079
biological_process	GO:0022607	cellular component assembly	0.169675612	0.996	0.51995079
biological_process	GO:0050789	regulation of biological process	0.169675612	0.996	0.51995079
biological_process	GO:0050794	regulation of cellular process	0.169675612	0.996	0.51995079
biological_process	GO:0051716	cellular response to stimulus	0.169675612	0.996	0.51995079
biological_process	GO:0007267	cell-cell signaling	0.169762992	0.996	0.51995079
biological_process	GO:0006913	nucleocytoplasmic transport	0.171163925	0.996	0.51995079
biological_process	GO:0051169	nuclear transport	0.171163925	0.996	0.51995079
biological_process	GO:0009056	catabolic process	0.208281416	0.998	0.617204168
biological_process	GO:0009987	cellular process	0.218865963	0.999	0.617204168
biological_process	GO:0022618	ribonucleoprotein complex assembly	0.222346843	0.999	0.617204168
biological_process	GO:0034622	cellular protein-containing complex assembly	0.222346843	0.999	0.617204168
biological_process	GO:0071826	ribonucleoprotein complex subunit organization	0.222346843	0.999	0.617204168
biological_process	GO:0006950	response to stress	0.229712924	0.999	0.626843742
biological_process	GO:0006259	DNA metabolic process	0.24781934	1	0.664981896
biological_process	GO:0032196	transposition	0.294699332	1	0.690037679
biological_process	GO:0021700	developmental maturation	0.298026763	1	0.690037679
biological_process	GO:0008219	cell death	0.311665952	1	0.690037679
biological_process	GO:0006605	protein targeting	0.321446124	1	0.690037679
biological_process	GO:0006886	intracellular protein transport	0.321446124	1	0.690037679
biological_process	GO:0008104	protein localization	0.321446124	1	0.690037679
biological_process	GO:0015031	protein transport	0.321446124	1	0.690037679
biological_process	GO:0015833	peptide transport	0.321446124	1	0.690037679
biological_process	GO:0033036	macromolecule localization	0.321446124	1	0.690037679
biological_process	GO:0034613	cellular protein localization	0.321446124	1	0.690037679
biological_process	GO:0042886	amide transport	0.321446124	1	0.690037679
biological_process	GO:0045184	establishment of protein localization	0.321446124	1	0.690037679
biological_process	GO:0070727	cellular macromolecule localization	0.321446124	1	0.690037679
biological_process	GO:0071702	organic substance transport	0.321446124	1	0.690037679
biological_process	GO:0071705	nitrogen compound transport	0.321446124	1	0.690037679
biological_process	GO:0007010	cytoskeleton organization	0.342899315	1	0.704966797
biological_process	GO:0006928	movement of cell or subcellular component	0.345915385	1	0.704966797
biological_process	GO:0048870	cell motility	0.345915385	1	0.704966797
biological_process	GO:0051674	localization of cell	0.345915385	1	0.704966797
biological_process	GO:0016043	cellular component organization	0.37086114	1	0.735241846
biological_process	GO:0044248	cellular catabolic process	0.378971723	1	0.735241846
biological_process	GO:0043933	protein-containing complex subunit organization	0.379037722	1	0.735241846
biological_process	GO:0065003	protein-containing complex assembly	0.379037722	1	0.735241846
biological_process	GO:0034330	cell junction organization	0.408051225	1	0.779464522

ontology	node_id	node_name	raw_p_overrep	FWER_ overrep	qvalue
biological_process	GO:0065007	biological regulation	0.411518536	1	0.779464522
biological_process	GO:0030154	cell differentiation	0.431082164	1	0.807025911
biological_process	GO:0044281	small molecule metabolic process	0.454207348	1	0.840544633
biological_process	GO:0002376	immune system process	0.47947758	1	0.855877206
biological_process	GO:0009058	biosynthetic process	0.484012399	1	0.855877206
biological_process	GO:0048869	cellular developmental process	0.48998348	1	0.855877206
biological_process	GO:0034641	cellular nitrogen compound metabolic process	0.491698637	1	0.855877206
biological_process	GO:0032501	multicellular organismal process	0.493778689	1	0.855877206
biological_process	GO:0051186	cofactor metabolic process	0.500302061	1	0.855877206
biological_process	GO:0051179	localization	0.506067352	1	0.855877206
biological_process	GO:0006810	transport	0.510336719	1	0.855877206
biological_process	GO:0051234	establishment of localization	0.510336719	1	0.855877206
biological_process	GO:0008283	cell proliferation	0.540928159	1	0.897829212
biological_process	GO:0005975	carbohydrate metabolic process	0.571096925	1	0.928753585
biological_process	GO:0040011	locomotion	0.571096925	1	0.928753585
biological_process	GO:0006790	sulfur compound metabolic process	0.597672708	1	0.952725801
biological_process	GO:0051604	protein maturation	0.597672708	1	0.952725801
biological_process	GO:0007049	cell cycle	0.605884081	1	0.954201271
biological_process	GO:0044085	cellular component biogenesis	0.616658575	1	0.954201271
biological_process	GO:0000902	cell morphogenesis	0.622305176	1	0.954201271
biological_process	GO:0032989	cellular component morphogenesis	0.622305176	1	0.954201271
biological_process	GO:0030705	cytoskeleton-dependent intracellular transport	0.650945344	1	0.979459817
biological_process	GO:0043473	pigmentation	0.650945344	1	0.979459817
biological_process	GO:0006457	protein folding	0.685092082	1	0.989918624
biological_process	GO:0044403	symbiont process	0.685092082	1	0.989918624
biological_process	GO:0044419	interspecies interaction between organisms	0.685092082	1	0.989918624
biological_process	GO:0051704	multi-organism process	0.685092082	1	0.989918624
biological_process	GO:0000278	mitotic cell cycle	0.688639043	1	0.989918624
biological_process	GO:0006091	generation of precursor metabolites and energy	0.714111747	1	1
biological_process	GO:0071840	cellular component organization or biogenesis	0.726857231	1	1
biological_process	GO:0007275	multicellular organism development	0.738620981	1	1
biological_process	GO:0009790	embryo development	0.738620981	1	1
biological_process	GO:0040007	growth	0.743213786	1	1
biological_process	GO:0061024	membrane organization	0.743213786	1	1
biological_process	GO:0007155	cell adhesion	0.755080374	1	1
biological_process	GO:0022610	biological adhesion	0.755080374	1	1
biological_process	GO:0006996	organelle organization	0.78198211	1	1
biological_process	GO:0007568	aging	0.794976783	1	1
biological_process	GO:0009653	anatomical structure morphogenesis	0.798641316	1	1
biological_process	GO:0022613	ribonucleoprotein complex biogenesis	0.81826044	1	1
biological_process	GO:0016192	vesicle-mediated transport	0.830541998	1	1
biological_process	GO:0051301	cell division	0.856452885	1	1
biological_process	GO:0006399	tRNA metabolic process	0.856521786	1	1
biological_process	GO:0034660	ncRNA metabolic process	0.856521786	1	1

ontology	node_id	node_name	raw_p_overrep	FWER_ overrep	qvalue
biological_process	GO:0000003	reproduction	0.856817158	1	1
biological_process	GO:0048646	anatomical structure formation involved in morphogenesis	0.875159595	1	1
biological_process	GO:0006914	autophagy	0.893953811	1	1
biological_process	GO:0007005	mitochondrion organization	0.893953811	1	1
biological_process	GO:0061919	process utilizing autophagic mechanism	0.893953811	1	1
biological_process	GO:0006412	translation	0.917917395	1	1
biological_process	GO:0006518	peptide metabolic process	0.917917395	1	1
biological_process	GO:0009059	macromolecule biosynthetic process	0.917917395	1	1
biological_process	GO:0034645	cellular macromolecule biosynthetic process	0.917917395	1	1
biological_process	GO:0043043	peptide biosynthetic process	0.917917395	1	1
biological_process	GO:0043603	cellular amide metabolic process	0.917917395	1	1
biological_process	GO:0043604	amide biosynthetic process	0.917917395	1	1
biological_process	GO:0044249	cellular biosynthetic process	0.917917395	1	1
biological_process	GO:0044271	cellular nitrogen compound biosynthetic process	0.917917395	1	1
biological_process	GO:1901566	organonitrogen compound biosynthetic process	0.917917395	1	1
biological_process	GO:1901576	organic substance biosynthetic process	0.917917395	1	1
biological_process	GO:0000280	nuclear division	0.930109073	1	1
biological_process	GO:0022402	cell cycle process	0.930109073	1	1
biological_process	GO:0048285	organelle fission	0.930109073	1	1
biological_process	GO:0140014	mitotic nuclear division	0.930109073	1	1
biological_process	GO:1903047	mitotic cell cycle process	0.930109073	1	1
biological_process	GO:0051276	chromosome organization	0.938812565	1	1
biological_process	GO:0055085	transmembrane transport	0.976561662	1	1
biological_process	GO:0042254	ribosome biogenesis	0.979812934	1	1
biological_process	GO:0042592	homeostatic process	0.990690553	1	1
biological_process	GO:0065008	regulation of biological quality	0.990690553	1	1
biological_process	GO:0008150	biological_process	1	1	1
biological_process	GO:0007009	plasma membrane organization	1	1	1
biological_process	GO:0010256	endomembrane system organization	1	1	1
biological_process	GO:0030198	extracellular matrix organization	1	1	1
biological_process	GO:0043062	extracellular structure organization	1	1	1
biological_process	GO:0007034	vacuolar transport	1	1	1
biological_process	GO:0007059	chromosome segregation	1	1	1

Table SI 16

Intensified selection enrichment

ontology	node_id	node_name	raw_p_overrep	FWER_overrep	qvalue
biological_process	GO:0044281	small molecule metabolic process	0.011067621	0.254	1
biological_process	GO:0006629	lipid metabolic process	0.027846065	0.514	1
biological_process	GO:0019439	aromatic compound catabolic process	0.09492738	0.904	1
biological_process	GO:0034655	nucleobase-containing compound catabolic process	0.09492738	0.904	1
biological_process	GO:0044270	cellular nitrogen compound catabolic process	0.09492738	0.904	1
biological_process	GO:0046700	heterocycle catabolic process	0.09492738	0.904	1
biological_process	GO:1901361	organic cyclic compound catabolic process	0.09492738	0.904	1
biological_process	GO:1901575	organic substance catabolic process	0.09492738	0.904	1
biological_process	GO:0006091	generation of precursor metabolites and energy	0.101687021	0.931	1
biological_process	GO:0042592	homeostatic process	0.140164105	0.982	1
biological_process	GO:0065008	regulation of biological quality	0.140164105	0.982	1
biological_process	GO:0044248	cellular catabolic process	0.167118394	0.993	1
biological_process	GO:0048856	anatomical structure development	0.187533085	0.995	1
biological_process	GO:0005975	carbohydrate metabolic process	0.232907102	0.998	1
biological_process	GO:0032502	developmental process	0.242998753	0.998	1
biological_process	GO:0009058	biosynthetic process	0.259914194	0.998	1
biological_process	GO:0051186	cofactor metabolic process	0.261725275	0.998	1
biological_process	GO:0040007	growth	0.331309947	1	1
biological_process	GO:0007267	cell-cell signaling	0.3353463	1	1
biological_process	GO:0009056	catabolic process	0.344198198	1	1
biological_process	GO:0007034	vacuolar transport	0.344986113	1	1
biological_process	GO:0042254	ribosome biogenesis	0.367968325	1	1
biological_process	GO:0007568	aging	0.379070546	1	1
biological_process	GO:0051276	chromosome organization	0.379934885	1	1
biological_process	GO:0006790	sulfur compound metabolic process	0.471442619	1	1
biological_process	GO:0006412	translation	0.505179796	1	1
biological_process	GO:0006518	peptide metabolic process	0.505179796	1	1
biological_process	GO:0009059	macromolecule biosynthetic process	0.505179796	1	1
biological_process	GO:0034645	cellular macromolecule biosynthetic process	0.505179796	1	1
biological_process	GO:0043043	peptide biosynthetic process	0.505179796	1	1
biological_process	GO:0043603	cellular amide metabolic process	0.505179796	1	1
biological_process	GO:0043604	amide biosynthetic process	0.505179796	1	1
biological_process	GO:0044249	cellular biosynthetic process	0.505179796	1	1
biological_process	GO:0044271	cellular nitrogen compound biosynthetic process	0.505179796	1	1
biological_process	GO:1901566	organonitrogen compound biosynthetic process	0.505179796	1	1
biological_process	GO:1901576	organic substance biosynthetic process	0.505179796	1	1
biological_process	GO:0006082	organic acid metabolic process	0.532810481	1	1
biological_process	GO:0006520	cellular amino acid metabolic process	0.532810481	1	1
biological_process	GO:0019752	carboxylic acid metabolic process	0.532810481	1	1
biological_process	GO:0043436	oxoacid metabolic process	0.532810481	1	1

ontology	node_id	node_name	raw_p_overrep	FWER_overrep	qvalue
biological_process	GO:0022613	ribonucleoprotein complex biogenesis	0.564343647	1	1
biological_process	GO:0048646	anatomical structure formation involved in morphogenesis	0.580974516	1	1
biological_process	GO:0008152	metabolic process	0.595379374	1	1
biological_process	GO:0040011	locomotion	0.618315427	1	1
biological_process	GO:0000003	reproduction	0.672156468	1	1
biological_process	GO:0055085	transmembrane transport	0.676217767	1	1
biological_process	GO:0034641	cellular nitrogen compound metabolic process	0.678136068	1	1
biological_process	GO:0002376	immune system process	0.693576721	1	1
biological_process	GO:0006914	autophagy	0.693576721	1	1
biological_process	GO:0007005	mitochondrion organization	0.693576721	1	1
biological_process	GO:0061919	process utilizing autophagic mechanism	0.693576721	1	1
biological_process	GO:0061024	membrane organization	0.710043392	1	1
biological_process	GO:0044085	cellular component biogenesis	0.737513994	1	1
biological_process	GO:0044237	cellular metabolic process	0.743553821	1	1
biological_process	GO:0006139	nucleobase-containing compound metabolic process	0.784949908	1	1
biological_process	GO:0006725	cellular aromatic compound metabolic process	0.784949908	1	1
biological_process	GO:0046483	heterocycle metabolic process	0.784949908	1	1
biological_process	GO:1901360	organic cyclic compound metabolic process	0.784949908	1	1
biological_process	GO:0009653	anatomical structure morphogenesis	0.789069148	1	1
biological_process	GO:0030154	cell differentiation	0.792339264	1	1
biological_process	GO:0007275	multicellular organism development	0.803764023	1	1
biological_process	GO:0009790	embryo development	0.803764023	1	1
biological_process	GO:0016192	vesicle-mediated transport	0.806760907	1	1
biological_process	GO:0048869	cellular developmental process	0.813402776	1	1
biological_process	GO:0006810	transport	0.839111243	1	1
biological_process	GO:0051234	establishment of localization	0.839111243	1	1
biological_process	GO:0006996	organelle organization	0.856528298	1	1
biological_process	GO:1901564	organonitrogen compound metabolic process	0.863971013	1	1
biological_process	GO:0006950	response to stress	0.86799866	1	1
biological_process	GO:0051179	localization	0.870925271	1	1
biological_process	GO:0044238	primary metabolic process	0.872496499	1	1
biological_process	GO:0071704	organic substance metabolic process	0.872496499	1	1
biological_process	GO:0044267	cellular protein metabolic process	0.873150409	1	1
biological_process	GO:0006259	DNA metabolic process	0.87543095	1	1
biological_process	GO:0071840	cellular component organization or biogenesis	0.875920169	1	1
biological_process	GO:0043933	protein-containing complex subunit organization	0.883303182	1	1
biological_process	GO:0065003	protein-containing complex assembly	0.883303182	1	1
biological_process	GO:0006807	nitrogen compound metabolic process	0.88591263	1	1
biological_process	GO:0044260	cellular macromolecule metabolic process	0.89079428	1	1
biological_process	GO:0032501	multicellular organismal process	0.895195592	1	1
biological_process	GO:0019538	protein metabolic process	0.898939637	1	1
biological_process	GO:0065007	biological regulation	0.914633112	1	1
biological_process	GO:0010467	gene expression	0.9153289	1	1

ontology	node_id	node_name	raw_p_overrep	FWER_overrep	qvalue
biological_process	GO:0007154	cell communication	0.949016686	1	1
biological_process	GO:0023052	signaling	0.949016686	1	1
biological_process	GO:0022607	cellular component assembly	0.952593999	1	1
biological_process	GO:0006464	cellular protein modification process	0.963660717	1	1
biological_process	GO:0036211	protein modification process	0.963660717	1	1
biological_process	GO:0043412	macromolecule modification	0.963660717	1	1
biological_process	GO:0016043	cellular component organization	0.978677687	1	1
biological_process	GO:0043170	macromolecule metabolic process	0.979157815	1	1
biological_process	GO:0009987	cellular process	0.988392955	1	1
biological_process	GO:0050896	response to stimulus	0.993527421	1	1
biological_process	GO:0090304	nucleic acid metabolic process	0.99378897	1	1
biological_process	GO:0008150	biological_process	1	1	1
biological_process	GO:0003013	circulatory system process	1	1	1
biological_process	GO:0007009	plasma membrane organization	1	1	1
biological_process	GO:0010256	endomembrane system organization	1	1	1
biological_process	GO:0030198	extracellular matrix organization	1	1	1
biological_process	GO:0032196	transposition	1	1	1
biological_process	GO:0043062	extracellular structure organization	1	1	1
biological_process	GO:0034330	cell junction organization	1	1	1
biological_process	GO:0030705	cytoskeleton-dependent intracellular transport	1	1	1
biological_process	GO:0043473	pigmentation	1	1	1
biological_process	GO:0007155	cell adhesion	1	1	1
biological_process	GO:0022610	biological adhesion	1	1	1
biological_process	GO:0006399	tRNA metabolic process	1	1	1
biological_process	GO:0034660	ncRNA metabolic process	1	1	1
biological_process	GO:0021700	developmental maturation	1	1	1
biological_process	GO:0051604	protein maturation	1	1	1
biological_process	GO:0003008	system process	1	1	1
biological_process	GO:0006928	movement of cell or subcellular component	1	1	1
biological_process	GO:0048870	cell motility	1	1	1
biological_process	GO:0050877	nervous system process	1	1	1
biological_process	GO:0051674	localization of cell	1	1	1
biological_process	GO:0006457	protein folding	1	1	1
biological_process	GO:0006913	nucleocytoplasmic transport	1	1	1
biological_process	GO:0044403	symbiont process	1	1	1
biological_process	GO:0044419	interspecies interaction between organisms	1	1	1
biological_process	GO:0051169	nuclear transport	1	1	1
biological_process	GO:0051704	multi-organism process	1	1	1
biological_process	GO:0000280	nuclear division	1	1	1
biological_process	GO:0022402	cell cycle process	1	1	1
biological_process	GO:0048285	organelle fission	1	1	1
biological_process	GO:0140014	mitotic nuclear division	1	1	1
biological_process	GO:1903047	mitotic cell cycle process	1	1	1
biological_process	GO:0007059	chromosome segregation	1	1	1
biological_process	GO:0006605	protein targeting	1	1	1

ontology	node_id	node_name	raw_p_overrep	FWER_overrep	qvalue
biological_process	GO:0006886	intracellular protein transport	1	1	1
biological_process	GO:0008104	protein localization	1	1	1
biological_process	GO:0015031	protein transport	1	1	1
biological_process	GO:0015833	peptide transport	1	1	1
biological_process	GO:0033036	macromolecule localization	1	1	1
biological_process	GO:0034613	cellular protein localization	1	1	1
biological_process	GO:0042886	amide transport	1	1	1
biological_process	GO:0045184	establishment of protein localization	1	1	1
biological_process	GO:0070727	cellular macromolecule localization	1	1	1
biological_process	GO:0071702	organic substance transport	1	1	1
biological_process	GO:0071705	nitrogen compound transport	1	1	1
biological_process	GO:0000902	cell morphogenesis	1	1	1
biological_process	GO:0022618	ribonucleoprotein complex assembly	1	1	1
biological_process	GO:0032989	cellular component morphogenesis	1	1	1
biological_process	GO:0034622	cellular protein-containing complex assembly	1	1	1
biological_process	GO:0071826	ribonucleoprotein complex subunit organization	1	1	1
biological_process	GO:0051301	cell division	1	1	1
biological_process	GO:0008219	cell death	1	1	1
biological_process	GO:0007010	cytoskeleton organization	1	1	1
biological_process	GO:0008283	cell proliferation	1	1	1
biological_process	GO:0000278	mitotic cell cycle	1	1	1
biological_process	GO:0046907	intracellular transport	1	1	1
biological_process	GO:0051641	cellular localization	1	1	1
biological_process	GO:0051649	establishment of localization in cell	1	1	1
biological_process	GO:0006396	RNA processing	1	1	1
biological_process	GO:0006397	mRNA processing	1	1	1
biological_process	GO:0016071	mRNA metabolic process	1	1	1
biological_process	GO:0016070	RNA metabolic process	1	1	1
biological_process	GO:0007049	cell cycle	1	1	1
biological_process	GO:0007165	signal transduction	1	1	1
biological_process	GO:0050789	regulation of biological process	1	1	1
biological_process	GO:0050794	regulation of cellular process	1	1	1
biological_process	GO:0051716	cellular response to stimulus	1	1	1

Table SI 17

Counting of nuclei in *Drosophila melanogaster*

Developmental stage	Estimated number of cells
Stage 13 embryo	~ 107,912
Stage 16 embryo/begin 1st instar larval stage	≥ 140,000
End 1st instar larval stage	≥ 200,000
End 2nd instar larval stage	≥ 370,000
End 3rd instar larval stage	≥ 400,000

Cell number estimates based on manual counting nuclei in 24 confocal planes of half a body.

Table SI 18

Histidine pathway genes

Species	*hutH* OG0005325	*hutU* OG0004955	*hutI* OG0006789	*FTCD* OG0005056
Demodex folliculorum	**0**	**0**	**0**	**0**
Dermatophagoides farinae	1	2	1	1
Ixodes scapularis	1	1	2	2
Metaseiulus occidentalis	1	1	1	1
Sarcoptes scabiei	1	1	1	1
Tetranychus urticae	1	0	0	0
Drosophila melanogaster	0	0	0	0
Parasteatoda tepidariorum	1	1	1	1
Limulus polyphemus	2	2	1	3
Stegodyphus mimosarum	2	1	1	1
Daphnia pulex	1	1	1	1
Tribolium castaneum	1	0	0	0
Tropilaelaps mercedesae	1	2	0	1
Centruroides sculpturatus	1	2	1	2
Varroa jacobsoni	1	1	1	1

References

Arribas P, Andújar C, Moraza ML, Linard B, Emerson BC, Vogler AP. 2020. Mitochondrial metagenomics reveals the ancient origin and phylodiversity of soil mites and provides a phylogeny of the acari. *Mol Biol Evol*. 37:683–694.

De Bie T, Cristianini N, Demuth JP, Hahn MW. 2006. CAFE: A computational tool for the study of gene family evolution. *Bioinformatics*. 22:1269–1271.

Greenhalgh R, Dermauw W, Glas JJ, Rombauts S, Wybouw N, Thomas J, Alba JM, Pritham EJ, Legarrea S, Feyereisen R, et al. 2020. Genome streamlining in a minute herbivore that manipulates its host plant. *eLife*. 9:e56689.

Klimov PB, OConnor BM, Chetverikov PE, Bolton SJ, Pepato AR, Mortazavi AL, Tolstikov AV, Bauchan GR, Ochoa R. 2018. Comprehensive phylogeny of acariform mites (Acariformes) provides insights on the origin of the four-legged mites (Eriophyoidea), a long branch. *Mol Phylogenet Evol*. 119:105-117.

Li F, Zhao X, Li M, He K, Huang C, Zhou Y, Li Z, Walters JR. 2019. Insect genomes: Progress and challenges. *Insect Mol Biol*. 28:739-758.

McKenna DD, Scully ED, Pauchet Y, Hoover K, Kirsch R, Geib SM, Mitchell RF, Waterhouse RM, Ahn S-J, Arsala D, et al. 2016. Genome of the Asian longhorned beetle (*Anoplophora glabripennis*), a globally significant invasive species, reveals key functional and evolutionary innovations at the beetle–plant interface. *Genome Biol*. 17:e227.

Sharma PP, Kaluziak ST, Pérez-Porro AR, González VL, Hormiga G, Wheeler WC, Giribet G. 2014. Phylogenomic interrogation of arachnida reveals systemic conflicts in phylogenetic signal. *Mol Biol Evol*. 31:2963–2984.
